# Cascade trifluoromethylthiolation and cyclization of *N*-[(3-aryl)propioloyl]indoles

**DOI:** 10.3762/bjoc.16.62

**Published:** 2020-04-08

**Authors:** Ming-Xi Bi, Shuai Liu, Yangen Huang, Xiu-Hua Xu, Feng-Ling Qing

**Affiliations:** 1 Key Laboratory of Science and Technology of Eco-Textiles, Ministry of Education, College of Chemistry, Chemical Engineering and Biotechnology, Donghua University, 2999 North Renmin Lu, Shanghai 201620, China; 2 Key Laboratory of Organofluorine Chemistry, Center for Excellence in Molecular Synthesis, Shanghai Institute of Organic Chemistry, University of Chinese Academy of Science, Chinese Academy of Science, 345 Lingling Lu, Shanghai 200032, China

**Keywords:** cyclization, indole derivatives, oxidation, radical reaction, trifluoromethylthiolation

## Abstract

A cascade oxidative trifluoromethylthiolation and cyclization of *N*-[(3-aryl)propioloyl]indoles with AgSCF_3_ is described. This protocol allows for the synthesis of novel bis(trifluoromethylthiolated) or trifluoromethylthiolated pyrrolo[1,2-*a*]indol-3-ones in moderate to good yields. Mechanistic investigations indicated that radical processes were probably involved in these transformations.

## Introduction

The trifluoromethylthio (SCF_3_) group could significantly improve the lipophilicity of organic molecules as shown by its high Hansch constant (π = 1.44 for SCF_3_, 0.88 for CF_3_, and 0.61 for SMe) [1] that helps permeation across biological membranes. Furthermore, the strong electron-withdrawing properties of the SCF_3_ group (Hammett constants: σ_p_ = 0.50, σ_m_ = 0.40) [2] with respect to metabolic stability have attracted considerable interest in pharmaceutical and agrochemical industries [3–5]. Traditional methods to access these compounds mainly include halogen–fluorine exchange of halomethyl sulfides and trifluoromethylation of sulfur-containing compounds [6–8]. Over the last decade, tremendous efforts have been triggered to develop methods for the direct incorporation of the SCF_3_ group into organic compounds [9–16], such as alkynes, alkenes, arenes, and alkanes. Despite these impressive advances, there is a continued strong demand for new methods that enable the efficient synthesis of SCF_3_-containing compounds, especially those featuring medicinally promising scaffolds.

**Figure 1:**
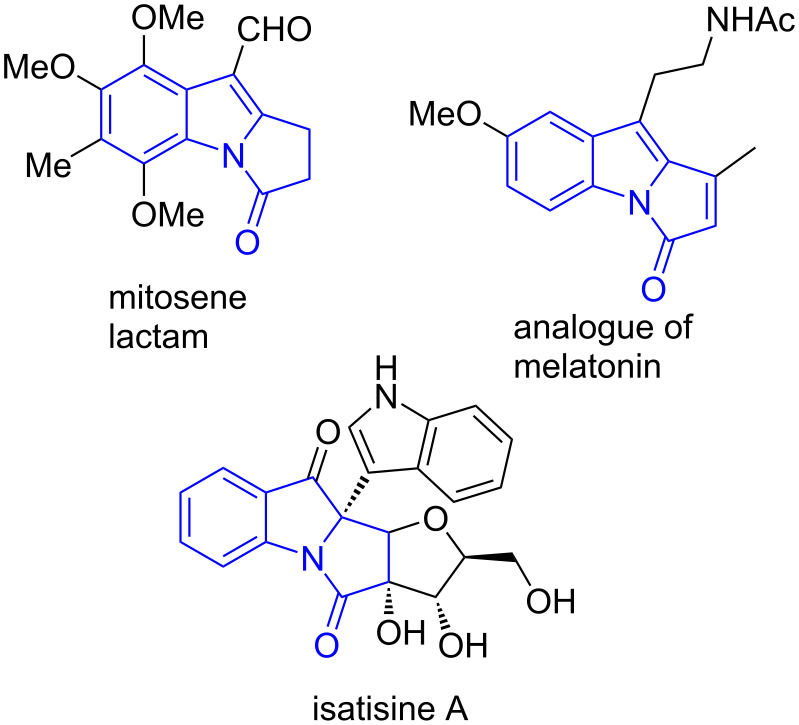
Representative examples of biologically active pyrrolo[1,2-*a*]indol-3-one derivatives.

Pyrrolo[1,2-*a*]indol-3-ones are prevalent scaffolds that widely exist in many bioactive compounds and natural products [17–20]. Representative examples of biologically active pyrrolo[1,2-*a*]indol-3-one derivatives are shown in Figure 1. Recently, the development of efficient methods for the synthesis of pyrrolo[1,2-*a*]indol-3-one derivatives has attracted considerable attention. For instance, Song [21] and Liang [22] reported the one-pot synthesis of novel phosphorylated and sulfonylated pyrrolo[1,2-*a*]indol-3-ones from *N*-[(3-phenyl)propioloyl]indole and *N*-propargylindoles, respectively. Inspired by these elegant results, we became interested in the preparation of SCF_3_-substituted pyrrolo[1,2-*a*]indol-3-ones, which might be potentially useful in medicinal chemistry.

**Scheme 1:**
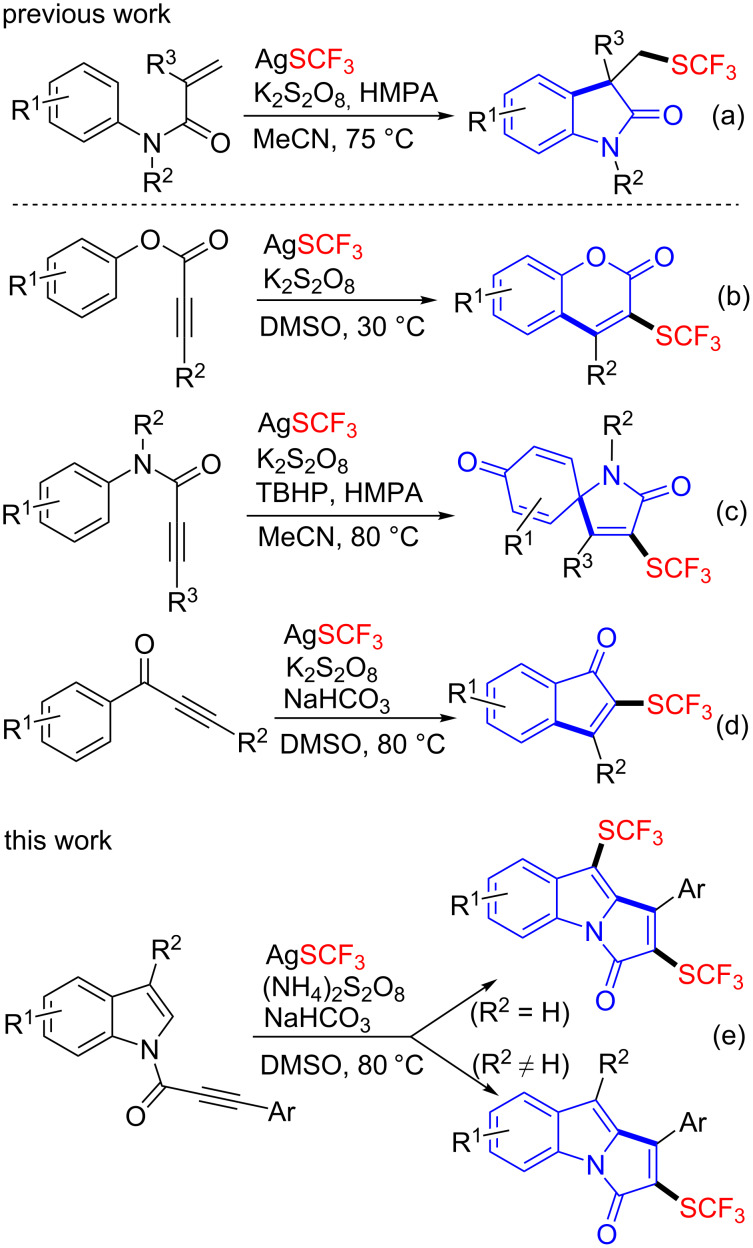
Radical cascade trifluoromethylthiolation and cyclization reactions.

Radical cascade reactions constitute highly efficient strategies for the construction of compounds with structural diversity and complexity. In 2014, Wang reported the first radical cascade trifluoromethylthiolation and cyclization of activated alkenes (Scheme 1a) [23]. Afterward, Nevado [24], Hopkinson and Glorius [25], Dagousset and Magnier [26], as well as Fu [27] applied this strategy in the synthesis of a series of CH_2_SCF_3_-substituted heterocycles. For the construction of SCF_3_-substituted cyclic compounds, normally proper alkynes are chosen as the substrates for cascade reactions [28–32]. In 2015, Wang developed an oxidative radical cyclization of aryl alkynoate esters with AgSCF_3_ for the synthesis of trifluoromethylthiolated coumarins (Scheme 1b) [28]. In 2016, Liu exploited the tandem trifluoromethylthiolation/cyclization of *N*-arylpropiolamides to construct the SCF_3_-substituted spiro[4,5]trienones (Scheme 1c) [29]. In the same year, Zhang and Chen disclosed the transformation of arylpropynones to SCF_3_-substituted indenones through the tandem trifluoromethylthiolation/cyclization processes (Scheme 1d) [30]. As part of our continuing research interest in radical trifluoromethylthiolation reactions [33–38], herein we disclose a cascade trifluoromethylthiolation and cyclization of *N*-[(3-aryl)propioloyl]indoles to access SCF_3_-substituted pyrrolo[1,2-*a*]indol-3-ones (Scheme 1e).

## Results and Discussion

On the outset, 1-(1*H*-indol-1-yl)-3-phenylprop-2-yn-1-one (**1a**) was chosen as the model substrate for optimization of the reaction conditions (Table 1). To our surprise, the reaction of **1a** and AgSCF_3_ in the presence of K_2_S_2_O_8_ and KHCO_3_ in DMSO at 80 °C gave bis(trifluoromethylthiolated) product **2a** in 28% yield (Table 1, entry 1). Only trace of mono(trifluoromethylthiolated) product was detected, and most of the substrate **1a** was not converted. To the best of our knowledge, the combination of bis(trifluoromethylthiolation) [36,39–41] and cascade cyclization reactions has not been reported before. Thus, the amounts of AgSCF_3_ and K_2_S_2_O_8_ were increased to deliver **2a** in 52% yield (Table 1, entry 2). Other oxidants including Na_2_S_2_O_8_ and (NH_4_)_2_S_2_O_8_ afforded **2a** in slightly higher yields, respectively (Table 1, entries 3 and 4). Switching KHCO_3_ to NaHCO_3_ could enhance the yield (Table 1, entry 6), whereas K_2_CO_3_ and DBU reduced the reaction efficiency (Table 1, entries 5 and 7). Subsequent evaluation of solvents revealed that MeCN and DMF were inferior to DMSO (Table 1, entries 8 and 9). Gratifyingly, the yield was improved to 80% by reducing the amount of base to 1.0 equivalent (Table 1, entry 10).

**Table 1:** Optimization of the reaction conditions^a^.

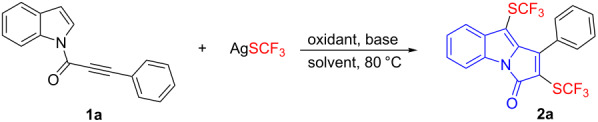				

entry	oxidant	base	solvent	yield (%)^b^

1	K_2_S_2_O_8_	KHCO_3_	DMSO	28
2^c^	K_2_S_2_O_8_	KHCO_3_	DMSO	52
3^c^	Na_2_S_2_O_8_	KHCO_3_	DMSO	55
4^c^	(NH_4_)_2_S_2_O_8_	KHCO_3_	DMSO	58
5^c^	(NH_4_)_2_S_2_O_8_	K_2_CO_3_	DMSO	40
6^c^	(NH_4_)_2_S_2_O_8_	NaHCO_3_	DMSO	72
7^c^	(NH_4_)_2_S_2_O_8_	DBU	DMSO	34
8^c^	(NH_4_)_2_S_2_O_8_	NaHCO_3_	MeCN	8
9^c^	(NH_4_)_2_S_2_O_8_	NaHCO_3_	DMF	trace
10^d^	(NH_4_)_2_S_2_O_8_	NaHCO_3_	DMSO	80

^a^Reaction conditions: **1a** (0.1 mmol), AgSCF_3_ (0.15 mmol), oxidant (0.2 mmol), base (0.15 mmol), solvent (2.0 mL), 80 °C, 12 h. ^b^Yield was determined by ^19^F NMR using trifluorotoluene as an internal standard. ^c^AgSCF_3_ (0.3 mmol), oxidant (0.3 mmol). ^d^AgSCF_3_ (0.3 mmol), oxidant (0.3 mmol), base (0.1 mmol).

**Scheme 2:**
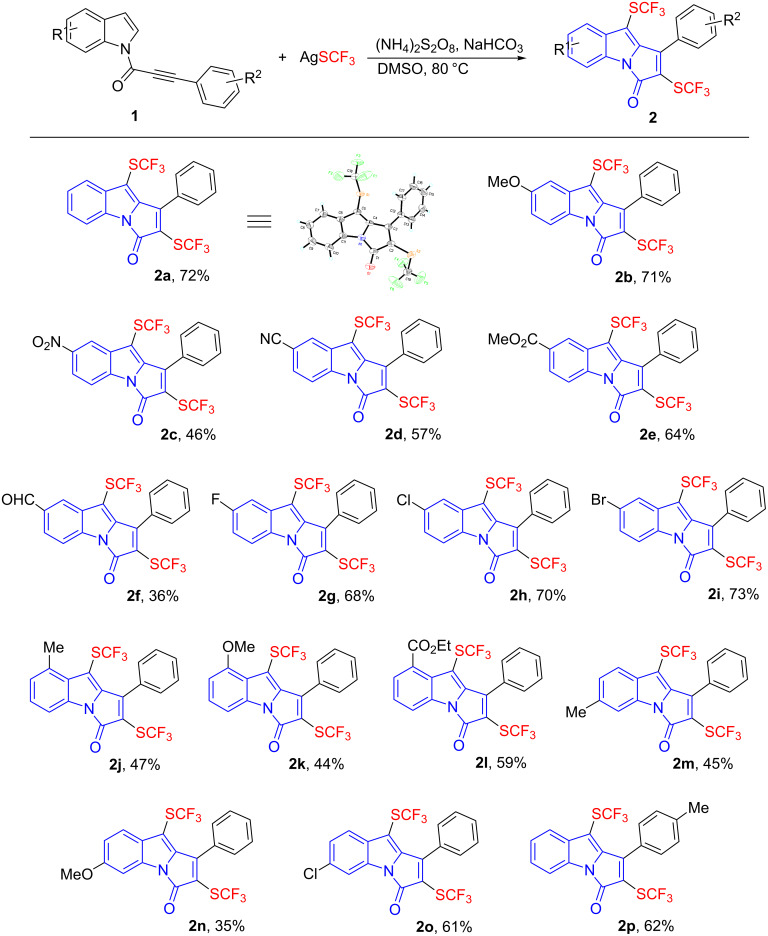
Cascade bis(trifluoromethylthiolation) and cyclization of *N*-[(3-aryl)propioloyl]indoles **1**. Reaction conditions*:* **1** (0.25 mmol), AgSCF_3_ (0.75 mmol), (NH_4_)_2_S_2_O_8_ (0.75 mmol), NaHCO_3_ (0.25 mmol), DMSO (5.0 mL), 80 °C, 12 h, isolated yields.

With the optimized reaction conditions in hand, we then set out to explore the substrate scope of *N*-[(3-aryl)propioloyl]indoles (Scheme 2). First, we explored the effect of the substitution on the indole ring. Both electron-donating and withdrawing groups at different positions of the indole ring produced the bis(trifluoromethylthiolated) products **2a**–**o** in moderate to good yields. A wide range of functionalities such as alkyl, alkoxy, nitro, nitrile, ester, aldehyde, fluoro, chloro, and bromo were well-tolerated and compatible under the mild reaction conditions. Substrate **1p** containing a methyl substituent on the phenyl ring could also participate in the reaction and furnish the desired product in moderate yield. However, attempts to prepare the substrates bearing an alkyl or electron-deficient aryl substituent on the alkynone were not successful. The structure of product **2a** was unambiguously identified by single-crystal X-ray analysis.

**Scheme 3:**
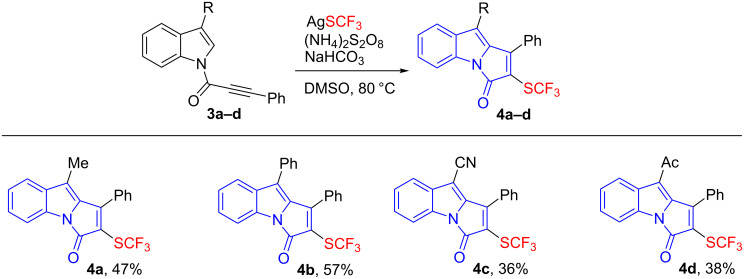
Cascade trifluoromethylthiolation and cyclization of *N*-[(3-aryl)propioloyl]indoles **3**. Reaction conditions: **3** (0.25 mmol), AgSCF_3_ (0.75 mmol), (NH_4_)_2_S_2_O_8_ (0.75 mmol), NaHCO_3_ (0.25 mmol), DMSO (5.0 mL), 80 °C, 12 h, isolated yields.

When the *N*-[(3-aryl)propioloyl]indole substrates (**3a**–**d**) with different substituents at the 3-position of the indole ring were subjected to the standard conditions, the cascade trifluoromethylthiolation and cyclization occurred to yield trifluoromethylthiolated pyrrolo[1,2-*a*]indol-3-ones (**4a**–**d**) in moderate yields (Scheme 3). The functionalities including alkyl, aryl, nitrile, and acyl were also well tolerated in this reaction.

**Scheme 4:**
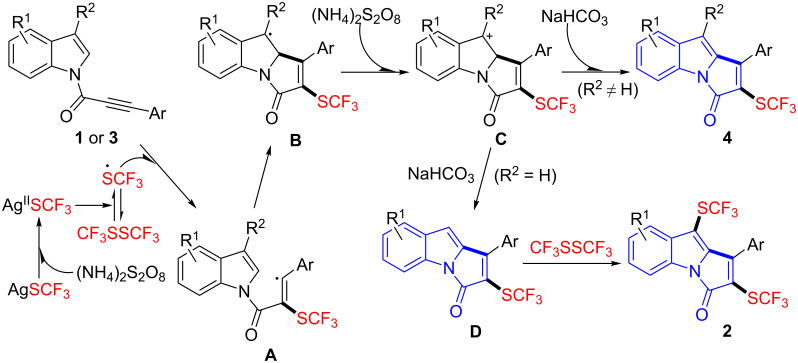
Proposed reaction mechanism.

In order to gain insight into the reaction mechanism, the radical scavenger 2,2,6,6-tetramethylpiperidin-1-oxyl (TEMPO) was added to the standard reactions of **1a** and **3b**, respectively. The desired product **2a** was not formed and only trace of **4b** was detected (see the Supporting Information File 1), which suggested that the radical process was probably involved in these transformations. Notably, no TEMPO-trapped product was detected by ^19^F NMR spectra of the crude reaction mixtures. On the basis of these results and literature studies [21,23,42–47], a plausible reaction mechanism was proposed in Scheme 4. First, oxidation of AgSCF_3_ by (NH_4_)_2_S_2_O_8_ generates Ag^II^SCF_3_, which could be further transformed to the CF_3_S radical or CF_3_SSCF_3_ [23,36]. Then, the addition of a CF_3_S radical to the alkyne function of substrates **1** or **3** afforded intermediate **A**. Subsequently, cyclization of intermediate **A**, followed by oxidation with (NH_4_)_2_S_2_O_8_, gave intermediate **C** [21,42–47]. Finally, deprotonation of intermediate **C** (R^2^ ≠ H) with NaHCO_3_ delivered the aromatized product **4**. In the case of intermediate **C** (R^2^ = H), intermediate **D** was probably formed, and further underwent electrophilic trifluoromethylthiolation with CF_3_SSCF_3_ [48–49] to furnish the bis(trifluoromethylthiolated) product **2**.

## Conclusion

We have reported the cascade trifluoromethylthiolation and cyclization reactions for the preparation of novel and potentially useful SCF_3_-containing pyrrolo[1,2-*a*]indol-3-ones. Oxidative trifluoromethylthiolation of *N*-[(3-aryl)propioloyl]indoles without substituent at the 3-position of the indole ring with AgSCF_3_ afforded the bis(trifluoromethylthiolated) products in moderate to good yields, whereas the substrates with a substituent at the 3-position of the indole ring were converted to the mono(trifluoromethylthiolated) products in moderate yields. Further studies on applying radical cascade reactions to the construction of fluorine-containing heterocyclic scaffolds are in progress in our laboratory.

## Supporting Information

File 1 Experimental procedures, spectroscopic and X-ray data (CCDC 1968129 for compound **2a**) and copies of NMR spectra.
