# Supplementary material for: Cascade trifluoromethylthiolation and cyclization of N-[(3-aryl)propioloyl]indoles
Source: Beilstein J Org Chem. 2020 Apr 8;16:657–62. doi: 10.3762/bjoc.16.62 (PMC7155893; doi:10.3762/bjoc.16.62)

BEILSTEIN JOURNAL OF ORGANIC CHEMISTRY

Supporting Information

for

Cascade trifluoromethylthiolation and cyclization of *N*-[(3-aryl)propioloyl]indoles

Ming-Xi Bi, Shuai Liu, Yangen Huang, Xiu-Hua Xu and Feng-Ling Qing

Beilstein J. Org. Chem. **2020,** *16,* 657–662. doi:10.3762/bjoc.16.62

Experimental procedures, spectroscopic and X-ray data (CCDC 1968129 for compound 2a) and copies of NMR spectra

License and Terms: This is a supporting information file under the terms of the Creative Commons Attribution License (http://creativecommons.org/licenses/by/4.0). Please note that the reuse, redistribution and reproduction in particular requires that the authors and source are credited.
The license is subject to the *Beilstein Journal of Organic Chemistry* terms and conditions: (https://www.beilstein-journals.org/bjoc)

Table of contents

1. General information……………………………………………………..…..…………S2

2. Preparation of substrates……………………………………………...………S2

3. General procedure for trifluoromethylthiolation and cyclization of *N*-[(3-aryl)propioloyl]indoles……………………………………...……………….S11

4. Mechanistic experiments……………………………………………………..…S20

5. References………………………………………………………...……..….…S22

6. ORTEP drawing of the X-ray crystallographic structure of 2a……………..S22

7. Copies of ^{1}H, ^{19}F, and ^{13}C NMR spectra for the products…………….……..…S23

1. General information

^1H and ^{19}F NMR ($CFCl_3$ as external standard and low field is positive) spectra were recorded on a Bruker AM 400 spectrometer. ^{13}C NMR spectra were recorded on a Bruker AM 400 or Bruker 600 spectrometers. Chemical shifts (δ) were reported in ppm, and coupling constants (*J*) were in Hertz (Hz). The following abbreviations were used to explain the multiplicities: s = singlet, d = doublet, t = triplet, q = quartet, m = multiplet, br = broad. The NMR yield was determined by ^{19}F NMR using (trifluoromethyl)benzene as an internal standard before working up the reaction. High resolution mass spectra (HRMS) were performed using a GC/MS TOF high-resolution mass spectrometer equipped with a liquid chromatography system or as electron spray ionization (ESI/HRMS) using a Thermo Fischer Scientific LTQ FT Ultra instrument in DART-positive mode.

Materials: Unless otherwise noted, all reagents were obtained commercially and used without further purification. Reactions were performed under an atmosphere of N_2 using glassware that flame-dried under vacuum. $AgSCF_3$ was prepared following the literature.

2. Preparation of substrates

General procedure for the preparation of *N*-[(3-phenyl)propioloyl]indoles [1]

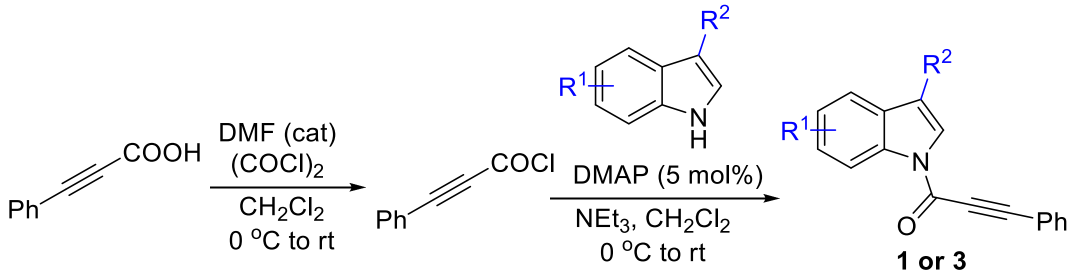

A solution of 3-phenylpropiolic acid (1.46 g, 10.0 mmol, 1.0 equiv) and DMF (4 drops) in CH_2Cl_2 (25 mL) was prepared and cooled to 0 °C. A bubbler was attached to the vessel and $(COCl)_2$ (1.69 mL, 20.0 mmol, 2.0 equiv) was added dropwise. After 5 minutes, the reaction was allowed to warm to room temperature and stirred for 1 hour. The acyl chloride was concentrated in vacuo and redissolved in CH_2Cl_2. A solution of the indoles (10.0 mmol, 1.0 equiv), DMAP (61.1 mg, 0.5 mmol, 0.05 equiv) and NEt_3 (2.78 mL, 20.0 mmol, 2.0 equiv) in CH_2Cl_2 (20 mL) was prepared and cooled to 0 °C. The acyl chloride solution was added dropwise into the vessel containing indoles. After

5 minutes, the reation was allowed to warm to room temperature and was stirred overnight. The reaction was quenched with a saturated $NaHCO_3$ solution and extracted with EtOAc. The combined organic layers were washed with brine, dried over Na_2SO_4 and concentrated in vacuo. The reaction was purified by a silica gel column chromatography to give product.

1-(1*H*-Indol-1-yl)-3-phenylprop-2-yn-1-one (1a)

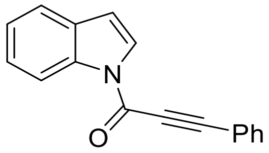

Yield: 593.6 mg, 24%; Yellow solid; m.p. 88-90 ºC (lit. [1]: 87–88 °C); ^{1}H NMR (400 MHz, $CDCl_3$) δ ppm 8.39 (d, *J* = 8.1 Hz, 1H), 7.74 (d, *J* = 3.8 Hz, 1H), 7.60-7.55 (m, 2H), 7.51-7.44 (m, 1H), 7.42-7.36 (m, 1H), 7.32 (t, *J* = 7.4 Hz, 2H), 7.29-7.24 (m, 1H), 7.25-7.16 (m, 1H), 6.58 (d, *J* = 3.8 Hz, 1H); ^{13}C NMR (101 MHz, $CDCl_3$) δ ppm 149.6, 134.0, 131.8, 130.1, 129.9, 127.7, 125.4, 124.1, 123.3, 119.9, 118.3, 115.6, 108.8, 90.8, 80.7. HRMS (ESI-TOF): *m/z* Calculated for $C_{17}H_{12}NO$ $[M+H]^+$: 246.0913; Found: 246.0914. Note: the IR data are compatible with the data reported in ref. [1].

1-(5-Methoxy-1*H*-indol-1-yl)-3-phenylprop-2-yn-1-one (1b)

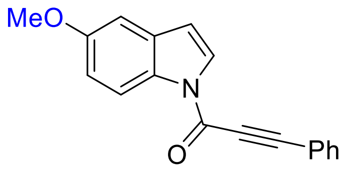

Yield: 605.2 mg, 22%; Yellow solid; m.p. 104-106 ºC; ^{1}H NMR (400 MHz, $CDCl_3$) δ ppm 8.29 (d, *J* = 8.9 Hz, 1H), 7.73 (d, *J* = 3.8 Hz, 1H), 7.63-7.56 (m, 2H), 7.45-7.39 (m, 1H), 7.39-7.35 (m, 2H), 6.96 (d, *J* = 2.5 Hz, 1H), 6.89 (dd, *J* = 9.0, 2.5 Hz, 1H), 6.54 (d, *J* = 3.8 Hz, 1H), 3.79 (s, 3H). ^{13}C NMR (101 MHz, $CDCl_3$) δ ppm 156.0, 131.8, 131.2, 129.9, 128.8, 127.7, 126.1, 118.4, 116.4, 112.3, 108.7, 103.0, 90.9, 80.7, 54.6. IR (thin film) *ν* 3065, 2923, 2830, 2204, 1657, 1581, 1476, 1382, 1279, 1092 cm^{-1}. HRMS (ESI-TOF): *m/z* Calculated for $C_{18}H_{14}NO_2$ $[M+H]^+$: 276.1019; Found: 276.1020.

1-(5-Nitro-1*H*-indol-1-yl)-3-phenylprop-2-yn-1-one (1c)

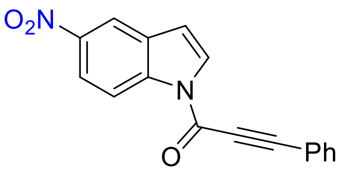

Yield: 498.6 mg, 17%; Yellow solid; m.p. 120-122 °C. ^{1}H NMR (400 MHz, $CDCl_3$) δ ppm 8.51 (d, *J* = 9.1 Hz, 1H), 8.42 (d, *J* = 2.2 Hz, 1H), 8.18 (dd, *J* = 9.1, 2.3 Hz, 1H), 7.94 (d, *J* = 3.8 Hz, 1H), 7.69-7.56 (m, 2H), 7.52-7.44 (m, 1H), 7.42-7.32 (m, 2H), 6.76 (d, *J* = 3.7 Hz, 1H). ^{13}C NMR (101 MHz, $CDCl_3$) δ ppm 149.5, 143.6, 137.0, 132.0, 130.5, 130.0, 128.2, 127.8, 119.4, 117.7, 116.1, 115.5, 108.8, 92.5, 80.1. IR (thin film) *v* 3123, 2951, 2261, 2207, 1971, 1675, 1518, 1445, 1324, 1194 cm^{-1}. HRMS (ESI-TOF): *m/z* Calculated for $C_{17}H_{11}N_2O_3$ $[M+H]^+$: 291.0764; Found: 291.0766.

1-(3-Phenylpropioloyl)-1*H*-indole-5-carbonitrile (1d)

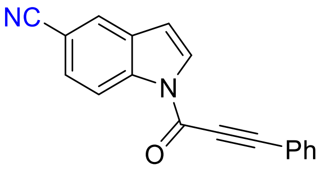

Yield: 830.7 mg, 31%; Yellow solid; m.p. 112-114 °C. ^{1}H NMR (400 MHz, $CDCl_3$) δ ppm 8.47 (d, *J* = 8.5 Hz, 1H), 7.89 (d, *J* = 3.8 Hz, 1H), 7.81 (s, 1H), 7.63-7.57 (m, 2H), 7.52 (dd, *J* = 8.6, 1.6 Hz, 1H), 7.48-7.42 (m, 1H), 7.41-7.33 (m, 2H), 6.66 (d, *J* = 3.8 Hz, 1H). ^{13}C NMR (101 MHz, $CDCl_3$) δ ppm 149.5, 135.8, 131.9, 130.4, 130.1, 127.8, 127.5, 127.3, 124.6, 118.3, 117.8, 116.2, 108.0, 106.7, 92.2, 80.2. IR (thin film) *v* 3159, 3144, 2243, 2231, 1663, 1612, 1488, 1463, 1364, 1191 cm^{-1}. HRMS (ESI-TOF): *m/z* Calculated for $C_{18}H_{11}N_2O$ $[M+H]^+$: 271.0866; Found: 271.0867.

Methyl 1-(3-phenylpropioloyl)-1*H*-indole-5-carboxylate (1e)

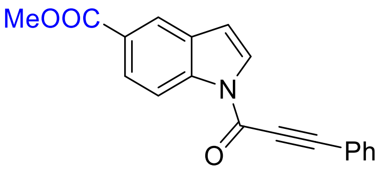

Yield: 1.03 g, 34%; Yellow solid; m.p. 110-112 °C. ^{1}H NMR (400 MHz, $CDCl_3$) δ ppm 8.41 (d, *J* = 8.7 Hz, 1H), 8.21 (d, *J* = 1.7 Hz, 1H), 7.97 (dd, *J* = 8.7, 1.7 Hz, 1H), 7.81 (d, *J* = 3.8 Hz, 1H), 7.60 (d, *J* = 6.9 Hz, 2H), 7.43 (t, *J* = 7.5 Hz, 1H), 7.35 (t, *J* = 7.4 Hz, 2H), 6.66 (d, *J* = 3.8 Hz, 1H), 3.86 (s, 3H). ^{13}C NMR (101 MHz, $CDCl_3$) δ 166.1,

149.6, 136.6, 131.9, 130.2, 129.9, 127.8, 126.6, 125.5, 125.2, 122.1, 118.0, 115.1, 109.0, 91.6, 80.5, 51.1. IR (thin film) *v* 3150, 3072, 2948, 2207, 1778, 1714, 1672, 1436, 1291, 1197 cm^{-1}. HRMS (ESI-TOF): *m/z* Calculated for $C_{19}H_{14}NO_3$ $[M+H]^+$: 304.0968; Found: 304.0969.

1-(3-Phenylpropioloyl)-1*H*-indole-5-carbaldehyde (1f)

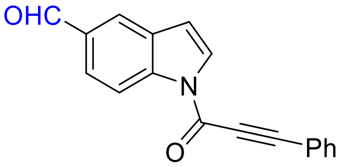

Yield: 818.4 mg, 30%; Yellow solid; m.p. 90-92 °C. **^{1}H NMR** (400 MHz, $CDCl_3$) δ ppm 9.97 (s, 1H), 8.49 (d, *J* = 8.5 Hz, 1H), 8.00 (s, 1H), 7.84 (d, *J* = 3.8 Hz, 1H), 7.79 (dd, *J* = 8.6, 1.6 Hz, 1H), 7.61-7.56 (m, 2H), 7.46-7.40 (m, 1H), 7.38-7.32 (m, 2H), 6.70 (d, *J* = 3.8 Hz, 1H). **^{13}C NMR** (101 MHz, $CDCl_3$) δ ppm 190.8, 149.6, 137.4, 131.9, 130.3, 127.8, 127.1, 125.4, 122.6, 117.9, 115.8, 108.9, 91.9, 80.4. **IR** (thin film) *v* 3395, 3371, 3147, 2745, 2216, 1702, 1669, 1436, 1185, 1061. **HRMS** (ESI-TOF): *m/z* Calculated for $C_{18}H_{12}NO_2$ $[M+H]^+$: 274.0863; Found: 274.0863.

1-(5-Fluoro-1*H*-indol-1-yl)-3-phenylprop-2-yn-1-one (1g)

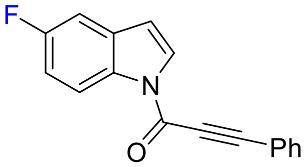

Yield: 1.05 g, 40%; Yellow solid; m.p. 95-97 °C; ^{1}H NMR (400 MHz, $CDCl_3$) δ ppm 8.30 (dd, *J* = 9.0, 4.7 Hz, 1H), 7.74 (d, *J* = 3.8 Hz, 1H), 7.62-7.47 (m, 2H), 7.42-7.34 (m, 1H), 7.34-7.24 (m, 2H), 7.10 (dd, *J* = 8.7, 2.5 Hz, 1H), 6.96 (td, *J* = 9.1, 2.6 Hz, 1H), 6.51 (d, *J* = 4.3 Hz, 1H). ^{19}F NMR (377 MHz, $CDCl_3$) δ ppm -117.86 (s, 1F). ^{13}C NMR (101 MHz, $CDCl_3$) δ 158.9 (d, *J* = 242.4 Hz), 149.4, 131.8, 131.1 (d, *J* = 10.1 Hz), 130.3, 130.0, 127.6, 126.9, 118.1, 116.5, 111.7 (d, *J* = 24.9 Hz), 108.3 (d, *J* = 3.8 Hz), 105.7 (d, *J* = 24.1 Hz), 90.9, 80.3. IR (thin film) *v* 3150, 3144, 3052, 2237, 2156, 1663, 1615, 1445, 1370, 1185 cm^{-1}. HRMS (ESI-TOF): *m/z* Calculated for $C_{17}H_{11}FNO$ $[M+H]^+$: 264.0819; Found: 264.0820.

1-(5-Chloro-1*H*-indol-1-yl)-3-phenylprop-2-yn-1-one (1h)

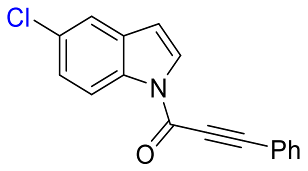

Yield: 390.9 mg, 14%; Yellow solid; m.p. 100-102 °C; ^{1}H NMR (400 MHz, $CDCl_3$) δ ppm 8.28 (d, *J* = 8.7 Hz, 1H), 7.74 (d, *J* = 3.8 Hz, 1H), 7.56 (d, *J* = 6.9 Hz, 2H), 7.44-7.37 (m, 2H), 7.34-7.31 (m, 2H), 7.20 (dd, *J* = 8.8, 2.1 Hz, 1H), 6.50 (d, *J* = 3.7 Hz, 1H). ^{13}C NMR (101 MHz, $CDCl_3$) δ ppm 149.4, 132.3, 131.8, 131.3, 130.1, 128.8, 127.7, 126.6, 124.2, 119.6, 118.1, 116.4, 107.9, 91.1, 80.4. IR (thin film) *v* 3117, 3069, 2234, 2195, 1663, 1448, 1358, 1201, 1080, 726 cm^{-1}. HRMS (ESI-TOF): *m/z* Calculated for $C_{17}H_{11}ClNO$ $[M+H]^+$: 280.0524; Found: 280.0521.

1-(5-Bromo-1*H*-indol-1-yl)-3-phenylprop-2-yn-1-one (1i)

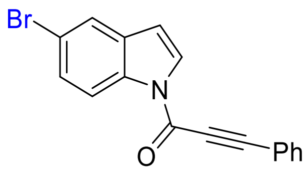

Yield: 450.8 mg, 14%; Yellow solid; m.p. 116-118 °C. ^{1}H NMR (400 MHz, $CDCl_3$) δ ppm 8.27 (d, *J* = 8.7 Hz, 1H), 7.77 (d, *J* = 3.8 Hz, 1H), 7.65-7.57 (m, 3H), 7.48-7.41 (m, 1H), 7.41-7.33 (m, 3H), 6.55 (d, *J* = 3.8 Hz, 1H). ^{13}C NMR (101 MHz, $CDCl_3$) δ ppm 149.6, 134.3, 131.8, 130.6, 130.1, 127.7, 126.2, 125.9, 125.1, 118.0, 114.4, 113.6, 108.3, 91.4, 80.4. IR (thin film) *v* 3102, 3059, 2207, 2165, 1666, 1530, 1436, 1185, 1080, 729 cm^{-1}. HRMS (ESI-TOF): *m/z* Calculated for $C_{17}H_{11}BrNO$ $[M+H]^+$: 324.0019; Found: 324.0019.

1-(4-Methyl-1*H*-indol-1-yl)-3-phenylprop-2-yn-1-one (1j)

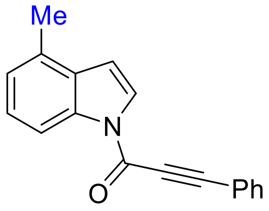

Yield: 414.2 mg, 16%; Yellow solid; m.p. 85-87 °C. ^{1}H NMR (400 MHz, $CDCl_3$) δ ppm 8.21 (d, *J* = 8.2 Hz, 1H), 7.70 (d, *J* = 3.9 Hz, 1H), 7.58-7.49 (m, 2H), 7.40-7.34 (m, 1H), 7.34-7.25 (m, 2H), 7.15 (t, *J* = 7.8 Hz, 1H), 6.99 (d, *J* = 7.3 Hz, 1H), 6.59 (d, *J* = 3.8 Hz, 1H), 2.41 (s, 3H). ^{13}C NMR (101 MHz, $CDCl_3$) δ ppm 149.7, 133.8, 131.8, 129.9,

129.6, 129.4, 127.6, 124.8, 124.2, 123.8, 118.3, 113.0, 107.1, 90.6, 80.8, 17.3. IR (thin film) *v* 3108, 3062, 2213, 1292, 1654, 1536, 1330, 1249, 1222, 1149 cm^{-1}. HRMS (ESI-TOF): *m/z* Calculated for $C_{18}H_{14}NO$ $[M+H]^+$: 260.1070; Found: 260.1070.

1-(4-Methoxy-1*H*-indol-1-yl)-3-phenylprop-2-yn-1-one (1k)

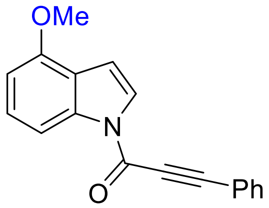

Yield: 358.9 mg, 13%; Yellow solid; m.p. 98-100 ºC. ^1H NMR (400 MHz, $CDCl_3$) δ ppm 8.00 (d, *J* = 8.3 Hz, 1H), 7.66 (d, *J* = 3.8 Hz, 1H), 7.59 (d, *J* = 6.9 Hz, 2H), 7.44-7.38 (m, 1H), 7.38-7.29 (m, 2H), 7.22 (t, *J* = 8.2 Hz, 1H), 6.73 (d, *J* = 3.8 Hz, 1H), 6.67 (d, *J* = 8.0 Hz, 1H), 3.86 (s, 3H). ^{13}C NMR (101 MHz, $CDCl_3$) δ ppm 151.7, 149.8, 135.2, 131.8, 129.9, 127.7, 125.2, 123.8, 120.1, 118.4, 108.6, 105.8, 103.9, 90.7, 80.8, 54.4. IR (thin film) *v* 3335, 3117, 3008, 2839, 2204, 1666, 1488, 1346, 1170, 1064 cm^{-1}. HRMS (ESI-TOF): *m/z* Calculated for $C_{18}H_{14}NO_2$ $[M+H]^+$: 276.1019; Found: 276.1019.

Ethyl 1-(3-phenylpropioloyl)-1*H*-indole-4-carboxylate (1l)

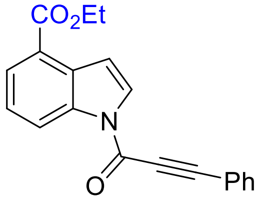

Yield: 1.04 g, 33%; Yellow solid; m.p. 120-122 ºC. ^1H NMR (400 MHz, $CDCl_3$) δ ppm 8.60 (d, *J* = 8.2 Hz, 1H), 7.95 (d, *J* = 1.0 Hz, 1H), 7.85 (d, *J* = 3.8 Hz, 1H), 7.63-7.53 (m, 2H), 7.47-7.38 (m, 1H), 7.37-7.25 (m, 4H), 4.35 (q, *J* = 7.1 Hz, 2H), 1.36 (t, *J* = 7.1 Hz, 3H). ^{13}C NMR (101 MHz, $CDCl_3$) δ ppm 165.4, 149.7, 134.7, 131.9, 130.2, 130.1, 127.7, 127.1, 125.8, 123.6, 121.4, 119.8, 118.1, 109.3, 91.2, 80.5, 59.8, 13.3. IR (thin film) *v* 2981, 2908, 2207, 1720, 1693, 1596, 1518, 1433, 1337, 1182 cm^{-1}. HRMS (ESI-TOF): *m/z* Calculated for $C_{20}H_{16}NO_3$ $[M+H]^+$: 318.1125; Found: 318.1126.

1-(6-Methyl-1*H*-indol-1-yl)-3-phenylprop-2-yn-1-one (1m)

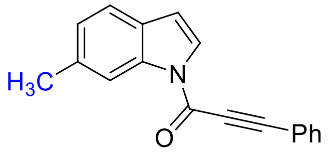

Yield: 316.9 mg, 12%; Yellow solid; m.p. 98-100 ºC. ^{1}H NMR (400 MHz, $CDCl_3$) δ ppm 8.25 (s, 1H), 7.71 (d, J = 3.8 Hz, 1H), 7.64-7.59 (m, 2H), 7.47-7.40 (m, 1H), 7.37 (t, J = 7.4 Hz, 3H), 7.07 (d, J = 7.9 Hz, 1H), 6.56 (d, J = 3.8 Hz, 1H), 2.43 (s, 3H). ^{13}C NMR (101 MHz, $CDCl_3$) δ ppm 149.8, 134.5, 134.4, 131.8, 129.9, 127.8, 127.7, 124.8, 119.5, 118.4, 116.1, 108.8, 90.5, 80.9, 20.8. IR (thin film) v 2972, 2920, 2216, 2023, 1965, 1711, 1663, 1330, 1197, 1110 cm^{-1}. HRMS (ESI-TOF): m/z Calculated for $C_{18}H_{14}NO$ [M+H]$^{+}$: 260.1070; Found: 260.1069.

1-(6-Methoxy-1*H*-indol-1-yl)-3-phenylprop-2-yn-1-one (1n)

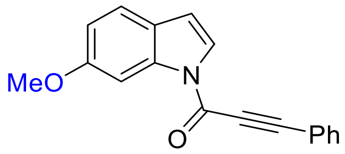

Yield: 528.6 mg, 19%; Yellow solid; m.p. 102-104 ºC. ^{1}H NMR (400 MHz, $CDCl_3$) δ 8.00 ppm (d, J = 2.3 Hz, 1H), 7.67 (d, J = 3.8 Hz, 1H), 7.61 (dt, J = 7.0, 1.4 Hz, 2H), 7.47-7.41 (m, 1H), 7.40-7.33 (m, 3H), 6.87 (dd, J = 8.5, 2.4 Hz, 1H), 6.54 (d, J = 3.7 Hz, 1H), 3.82 (s, 3H). ^{13}C NMR (101 MHz, $CDCl_3$) δ ppm 157.3, 149.9, 135.1, 131.8, 129.9, 127.7, 124.2, 123.6, 120.3, 118.4, 112.5, 108.6, 99.8, 90.5, 80.9, 54.7. IR (thin film) v 3055, 2922, 2830, 2220, 1657, 1555, 1476, 1330, 1250, 1092 cm^{-1}. HRMS (ESI-TOF): m/z Calculated for $C_{18}H_{14}NO_2$ [M+H]$^{+}$: 276.1019; Found: 276.1020.

1-(6-Chloro-1*H*-indol-1-yl)-3-phenylprop-2-yn-1-one (1o)

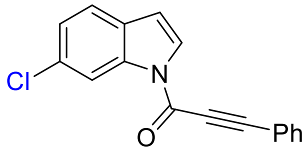

Yield: 893.8 mg, 32%; Yellow solid; m.p. 108-110 ºC. ^{1}H NMR (400 MHz, $CDCl_3$) δ ppm 8.46 (s, 1H), 7.77 (d, J = 3.8 Hz, 1H), 7.62 (d, J = 7.1 Hz, 2H), 7.50-7.31 (m, 4H), 7.22 (dd, J = 8.4, 1.9 Hz, 1H), 6.59 (d, J = 3.8 Hz, 1H). ^{13}C NMR (101 MHz, $CDCl_3$) δ ppm 149.6, 136.6, 134.4, 131.9, 130.2, 128.6, 127.8, 126.0, 123.9, 120.7, 118.2, 115.8, 108.3, 92.4, 80.1. IR (thin film) v 3156, 3065, 2189, 2204, 1666, 1533, 1324, 1185, 1074, 759 cm^{-1}. HRMS (ESI-TOF): m/z Calculated for $C_{17}H_{11}ClNO$ [M+H]$^{+}$: 280.0524; Found: 280.0525.

1-(3-Methyl-1*H*-indol-1-yl)-3-phenylprop-2-yn-1-one (3a)

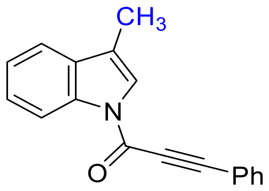

Yield: 621.5 mg, 24%; Yellow liquid. ^{1}H NMR (400 MHz, $CDCl_3$) δ ppm 8.45 (d, *J* = 6.8 Hz, 1H), 7.68-7.63 (m, 2H), 7.56 (s, 1H), 7.47 (t, *J* = 7.5 Hz, 2H), 7.44-7.37 (m, 2H), 7.38-7.26 (m, 2H), 2.28 (s, 3H). ^{13}C NMR (101 MHz, $CDCl_3$) δ ppm 150.5, 135.5, 132.9, 132.3, 131.0, 128.8, 125.3, 124.3, 123.3, 119.6, 119.3, 119.1, 116.8, 91.3, 81.9, 9.8. IR (thin film) *ν* 2210, 2005, 1651, 1500, 1388, 1352, 1188, 1074, 898, 735 cm^{-1}. HRMS (ESI-TOF): *m/z* Calculated for $C_{18}H_{14}NO$ $[M+H]^+$: 260.1070; Found: 260.1071.

3-Phenyl-1-(3-phenyl-1*H*-indol-1-yl)prop-2-yn-1-one (3b)

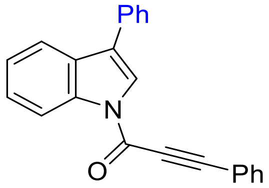

Yield: 867.4 mg, 27%; Yellow solid; m.p. 110-112 °C. ^{1}H NMR (400 MHz, $CDCl_3$) δ ppm 8.49 (d, *J* = 8.1 Hz, 1H), 7.85 (s, 1H), 7.73 (d, *J* = 7.8 Hz, 1H), 7.60 (t, *J* = 6.5 Hz, 4H), 7.44-7.27 (m, 8H). ^{13}C NMR (101 MHz, $CDCl_3$) δ ppm 149.6, 134.8, 132.1, 131.8, 130.0, 128.8, 127.9, 127.7, 127.0, 126.6, 124.6, 123.7, 123.6, 122.0, 119.0, 118.3, 115.9, 90.9, 80.8. IR (thin film) *ν* 3075, 2216, 1726, 1678, 1609, 1494, 1460, 1396, 1276, 1210 cm^{-1}. HRMS (ESI-TOF): *m/z* Calculated for $C_{23}H_{16}NO$ $[M+H]^+$: 322.1226; Found: 322.1227.

1-(3-Phenylpropioloyl)-1*H*-indole-3-carbonitrile (3c)

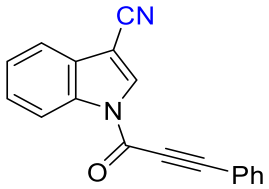

Yield: 643.3 mg, 24%; Yellow solid; m.p. 100-102 °C. ^{1}H NMR (400 MHz, $CDCl_3$) δ ppm 8.42 (d, *J* = 8.3 Hz, 1H), 8.31 (s, 1H), 7.72-7.61 (m, 3H), 7.54-7.45 (m, 1H), 7.45-7.36 (m, 4H). ^{13}C NMR (101 MHz, $CDCl_3$) δ ppm 150.4, 135.4, 132.8, 132.2, 130.9, 128.7, 125.3, 124.2, 123.2, 119.6, 119.2, 119.0, 116.8, 91.2, 81.9, 29.7. IR (thin film) *ν*

3150, 3062, 2249, 2198, 1687, 1606, 1442, 1361, 1182, 1040 cm^{-1}. HRMS (ESI-TOF): *m/z* Calculated for $C_{18}H_{11}N_2O$ $[M+H]^+$: 271.0866; Found: 271.0867.

1-(3-Acetyl-1*H*-indol-1-yl)-3-phenylprop-2-yn-1-one (3d)

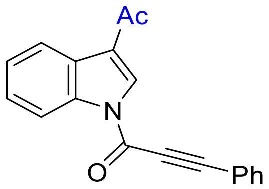

Yield: 918.1 mg, 32%; Yellow solid; m.p. 104-106 °C. ^{1}H NMR (400 MHz, $CDCl_3$) δ ppm 8.44-8.22 (m, 3H), 7.63 (d, *J* = 7.5 Hz, 2H), 7.47 (t, *J* = 7.5 Hz, 1H), 7.43-7.28 (m, 4H), 2.53 (s, 3H). ^{13}C NMR (101 MHz, $CDCl_3$) δ ppm 192.5, 149.6, 134.5, 131.9, 131.1, 130.5, 127.8, 126.8, 125.3, 124.7, 121.7, 121.3, 117.7, 115.0, 92.4, 80.1, 26.8. IR (thin film) *v* 2954, 2225, 2138, 1720, 1684, 1503, 1435, 1197, 1083, 956 cm^{-1}. HRMS (ESI-TOF): *m/z* Calculated for $C_{19}H_{14}NO_2$ $[M+H]^+$: 288.1019; Found: 288.1020.

1-(1*H*-Indol-1-yl)-3-(*p*-tolyl)prop-2-yn-1-one (1p) [2]

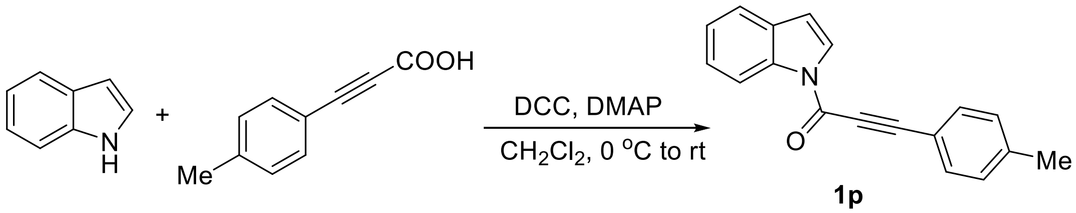

To a solution of indole (292.8 mg, 2.5 mmol, 1.0 equiv) in CH_2Cl_2 (15 mL) was added 3-*p*-tolylpropiolic acid (440.6 mg, 2.75 mmol, 1.1 equiv.) at 0 °C, then a mixture of DCC (773.6 mg, 3.75 mmol, 1.5 equiv) and DMAP (30.6 mg, 0.25 mmol, 0.1 equiv) in CH_2Cl_2 (10 mL) was added dropwise. The mixture was stirred at room temperature for 12 hours. Then, the crude was filtered and washed with CH_2Cl_2 and concentrated. The residue was purified by silica gel chromatography (hexane: EtOAc = 5:1) to give **1p** (194.5 mg, 30%) as a yellow solid. m.p. 98-100 °C; ^{1}H NMR (400 MHz, $CDCl_3$) δ ppm 8.52 (d, *J* = 8.2 Hz, 1H), 7.89 (s, 1H), 7.61 (d, *J* = 7.7 Hz, 3H), 7.41 (t, *J* = 7.8 Hz, 1H), 7.34 (t, *J* = 7.6 Hz, 1H), 7.31-7.24 (m, 3H), 2.44 (s, 3H). ^{13}C NMR (101 MHz, $CDCl_3$) δ ppm 150.9, 141.8, 135.1, 132.9, 131.1, 129.5, 126.6, 125.2, 124.3, 121.0, 116.7, 116.3, 109.7, 92.3, 81.5, 21.8. IR (thin film) *v* 2917, 2204, 1748, 1669, 1590, 1497, 1442, 1346, 1089, 747 cm^{-1}. HRMS (ESI-TOF): *m/z* Calculated for $C_{18}H_{14}NO$ $[M+H]^+$: 260.1070; Found: 260.1068.

3. **General procedure for trifluoromethylthiolation and cyclization of *N*-[(3-aryl)propioloyl]indoles**

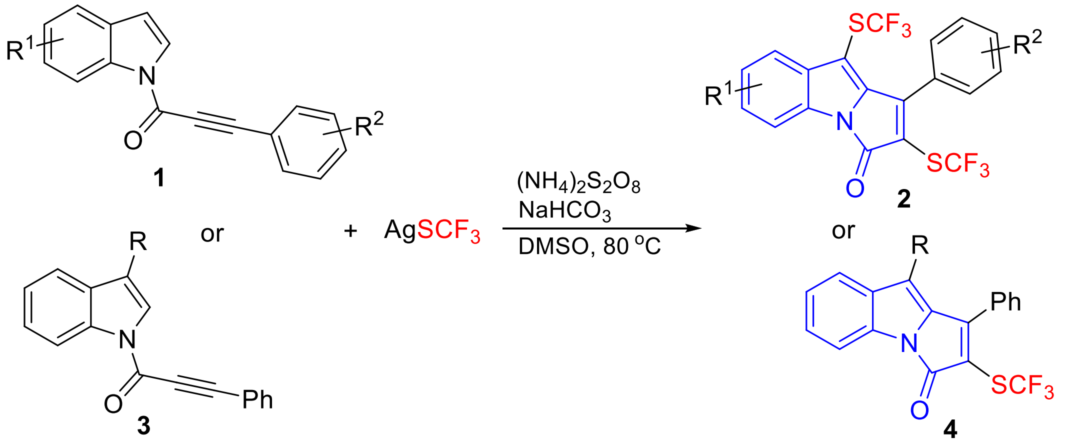

A 25 mL Schlenk tube equipped with a magnetic stir bar was charged with **1** or **3** (0.25 mmol, 1.0 equiv), $AgSCF_3$ (156.7 mg, 0.75 mmol, 3.0 equiv), $(NH_4)_2S_2O_8$ (171.2 mg, 0.75 mmol, 3.0 equiv) and $NaHCO_3$ (21.0 mg, 0.25 mmol, 1.0 equiv). The tube was sealed with a septum, evacuated and backfilled with nitrogen three times. Then DMSO (5.0 mL) was added by a syringe. The mixture was stirred at 80 °C for 12 h. A saturated ammonium chloride aqueous solution (5.0 mL) was added. The resulting mixture was filtered by Celite, eluted with ethyl acetate. The organic phase was washed with brine, dried over Na_2SO_4, filtered, and concentrated under reduced vacuum. The residue purified with silica gel column chromatography to provide the pure product.

1-Phenyl-2,9-bis(trifluoromethylthio)-3*H*-pyrrolo[1,2-*a*]indol-3-one (2a)

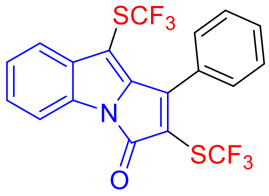

Yield: 80.1 mg, 72%; Red solid; m.p. 118-120 °C. ^{1}H NMR (400 MHz, $CDCl_3$) δ ppm 7.79 (d, *J* = 8.1 Hz, 1H), 7.58 (d, *J* = 7.9 Hz, 1H), 7.52-7.46 (m, 5H), 7.39 (t, *J* = 7.8 Hz, 1H), 7.22 (d, *J* = 7.9 Hz, 1H). ^{19}F NMR (377 MHz, $CDCl_3$) δ ppm -39.99 (s, 3F), -40.88 (s, 3F). ^{13}C NMR (101 MHz, CDCl3) δ ppm 160.1, 157.3, 142.1, 133.6, 133.2, 130.2, 128.7, 128.0, 127.4, 127.3 (q, *J* = 314 Hz), 127.2 (q, *J* = 313 Hz), 127.0, 123.8, 121.2, 118.6, 112.1, 105.3 (q, *J* = 2.6 Hz). IR (thin film) *ν* 2957, 2933, 2917, 2011, 1748, 1606, 1542, 1442, 1264, 1080 cm^{-1}. HRMS (ESI-TOF): *m/z* Calculated for $C_{19}H_{10}F_6NOS_2$ $[M+H]^+$: 446.0103; Found: 446.0130.

7-Methoxy-1-phenyl-2,9-bis(trifluoromethylthio)-3*H*-pyrrolo[1,2-*a*]indol-3-one (2b)

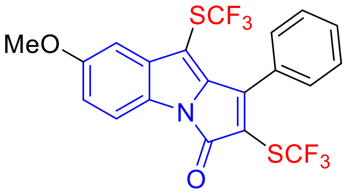

Yield: 84.3 mg, 71%; Red solid; m.p. 110-112 °C. ^{1}H NMR (400 MHz, $CDCl_3$) δ ppm 7.70 (d, *J* = 8.8 Hz, 1H), 7.62-7.49 (m, 5H), 7.07 (s, 1H), 7.03 (d, *J* = 8.8 Hz, 1H), 3.84 (s, 3H). ^{19}F NMR (377 MHz, $CDCl_3$) δ ppm -40.11 (s, 3F), -40.94 (s, 3F). ^{13}C NMR (101 MHz, $CDCl_3$) δ ppm 161.0, 158.5, 157.4, 143.7, 135.8, 131.2, 129.1, 128.8, 128.4, 128.4 (q, *J* = 313.1 Hz), 128.1, 119.2, 118.4, 113.8, 105.8 (q, *J* = 2.7 Hz), 104.7, 55.7. IR (thin film) *v* 3078, 2951, 2917, 2210, 2077, 2008, 1745, 1167, 1128, 1077 cm^{-1}. HRMS (ESI-TOF): *m/z* Calculated for $C_{20}H_{12}F_6NO_2S_2$ $[M+H]^+$: 476.0208; Found: 476.0207.

7-Nitro-1-phenyl-2,9-bis(trifluoromethylthio)-3*H*-pyrrolo[1,2-a]indol-3-one (2c)

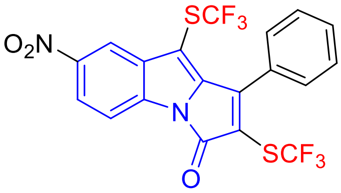

Yield: 56.6 mg, 46%; Red solid; m.p. 116-118 °C. ^{1}H NMR (400 MHz, $CDCl_3$) δ ppm 8.51 (s, 1H), 8.32 (d, *J* = 10.8 Hz, 1H), 7.94 (d, *J* = 8.9 Hz, 1H), 7.59-7.47 (m, 5H). ^{19}F NMR (377 MHz, $CDCl_3$) δ ppm -39.39 (s, 3F), -40.69 (s, 3F). ^{13}C NMR (101 MHz, $CDCl_3$) δ ppm 159.5, 157.4, 144.4, 144.3, 135.7, 134.0, 130.7, 128.0, 127.6, 127.1 (q, *J* = 314.1 Hz), 127.0 (q, *J* = 313.1Hz), 126.3, 123.9, 120.3, 117.4, 112.3, 105.1 (q, *J* = 2.6 Hz). IR (thin film) *v* 2929, 2144, 1736, 1612, 1533, 1334, 1167, 1140, 1089, 1007, 741 cm^{-1}. HRMS (ESI-TOF): *m/z* Calculated for $C_{19}H_9F_6N_2O_3S_2$ $[M+H]^+$: 490.9953; Found: 490.9950.

3-Oxo-1-phenyl-2,9-bis(trifluoromethylthio)-3*H*-pyrrolo[1,2-*a*]indole-7-carbonitrile (2d)

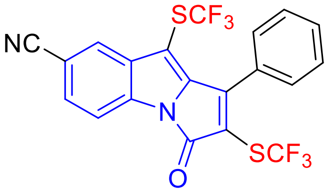

Yield: 66.8 mg, 57%; Red solid; m.p. 108-110 °C. ^{1}H NMR (400 MHz, $CDCl_3$) δ ppm 7.97-7.87 (m, 2H), 7.67 (d, J = 8.5 Hz, 1H), 7.58-7.42 (m, 5H). ^{19}F NMR (377 MHz, $CDCl_3$) δ ppm -39.47 (s, 3F), -40.70 (s, 3F). ^{13}C NMR (101 MHz, $CDCl_3$) δ ppm 159.6, 157.3, 143.8, 134.6, 133.9, 131.7, 130.7, 128.0, 127.6, 127.1 (q, J = 313.1 Hz), 127.0 (q, J = 313.1 Hz), 126.4, 125.7, 120.1 (d, J = 2.4 Hz), 117.3, 112.9, 107.7, 104.3 (q, J = 2.7 Hz). IR (thin film) v 1078, 2963, 2869, 2192, 2014, 1736, 1615, 1346, 1077, 1019 cm^{-1}. HRMS (ESI-TOF): m/z Calculated for $C_{20}H_9F_6NOS_2$ $[M+H]^+$: 471.0055; Found: 471.0054.

Methyl 3-oxo-1-phenyl-2,9-bis(trifluoromethylthio)-3*H*-pyrrolo[1,2-*a*]indole-7-carboxylate (2e)

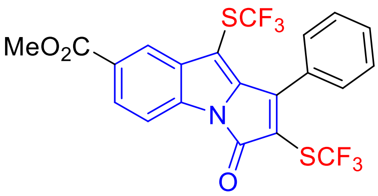

Yield: 80.9 mg, 64%; Red solid; m.p. 128-130 °C. ^{1}H NMR (400 MHz, $CDCl_3$) δ ppm 8.29 (s, 1H), 8.10 (dd, J = 8.4, 1.6 Hz, 1H), 7.82 (d, J = 8.4 Hz, 1H), 7.55-7.44 (m, 5H), 3.87 (s, 3H). ^{19}F NMR (377 MHz, $CDCl_3$) δ ppm -39.73 (s, 3F), -40.83 (s, 3F). ^{13}C NMR (101 MHz, $CDCl_3$) δ ppm 165.2, 159.8, 157.4, 143.1, 135.4, 133.7, 130.4, 130.0, 128.0, 127.2 (q, J = 314.1 Hz), 127.1 (q, J = 313.1 Hz), 127.5, 126.7, 126.0, 123.1, 119.3, 111.8, 105.4 (q, J = 2.6 Hz), 51.3. IR (thin film) v 2960, 2201, 2153, 1760, 1621, 1533, 1439, 1294, 1089 cm^{-1}. HRMS (ESI-TOF): m/z Calculated for $C_{21}H_{12}F_6NO_3S_2$ $[M+H]^+$: 504.0157; Found: 504.0153.

3-Oxo-1-phenyl-2,9-bis(trifluoromethylthio)-3*H*-pyrrolo[1,2-*a*]indole-7-carbaldehyde (2f)

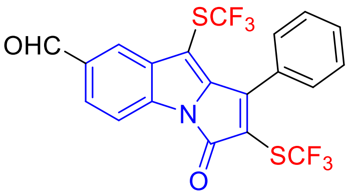

Yield: 42.7 mg, 36%; Red solid; m.p. 122-124 °C. ^{1}H NMR (400 MHz, $CDCl_3$) δ ppm 9.99 (s, 1H), 8.13 (s, 1H), 7.96 (s, 2H), 7.57-7.45 (m, 5H). ^{19}F NMR (377 MHz, $CDCl_3$) δ ppm -39.60 (s, 3F), -40.72 (s, 3F). **^{13}C NMR** (101 MHz, $CDCl_3$) δ ppm 189.7, 159.8, 157.4, 143.4, 136.2, 134.2, 132.4, 130.5, 129.7, 128.0, 127.5, 127.2 (q, J = 314.1 Hz), 127.1 (q, J = 313.1 Hz), 126.6, 123.6, 119.7 (q, J = 2.4 Hz), 112.6, 105.4. IR (thin film)

ν 2842, 2796, 2159, 1754, 1736, 1702, 1603, 1527, 1307, 1128, 1086, 916 cm^{-1}. **HRMS** (ESI-TOF): *m/z* Calculated for $C_{20}H_{10}F_6NO_2S_2$ $[M+H]^+$: 474.0052; Found: 474.0048.

7-Fluoro-1-phenyl-2,9-bis(trifluoromethylthio)-3*H*-pyrrolo[1,2-*a*]indol-3-one (2g)

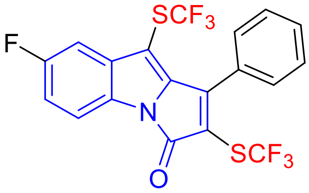

Yield: 78.4 mg, 68%; Red solid; m.p. 120-122 °C. ^1H NMR (400 MHz, $CDCl_3$) δ ppm 7.75 (dd, *J* = 8.8, 4.2 Hz, 1H), 7.54-7.44 (m, 5H), 7.27 (d, *J* = 6.0 Hz, 1H), 7.13 (td, *J* = 8.9, 2.5 Hz, 1H). ^{19}F NMR (377 MHz, $CDCl_3$) δ ppm -39.84 (s, 3F), -40.90 (s, 3F), -116.24 to -116.29 (m, 1F). ^{13}C NMR (101 MHz, $CDCl_3$) δ ppm 159.9, 159.2 (d, *J* = 243.4 Hz), 157.4, 143.5, 135.0 (d, *J* = 10.1 Hz), 130.4, 129.5, 128.0, 127.5, 127.2 (q, *J* = 313.1 Hz), 127.1 (q, *J* = 313.1 Hz), 126.8, 118.9, 116.5 (d, *J* = 25.7 Hz), 113.0 (d, *J* = 9.1 Hz), 107.3 (d, *J* = 25.6 Hz), 104.6 (q, *J* = 2.6 Hz). IR (thin film) ν 2969, 2290, 2356, 2174, 1757, 1578, 1557, 1470, 1349, 1258, 1149, 1083 cm^{-1}. HRMS (ESI-TOF): *m/z* Calculated for $C_{19}H_9F_7NOS_2$ $[M+H]^+$: 464.0008; Found: 464.0006.

7-Chloro-1-phenyl-2,9-bis(trifluoromethylthio)-3*H*-pyrrolo[1,2-*a*]indol-3-one (2h)

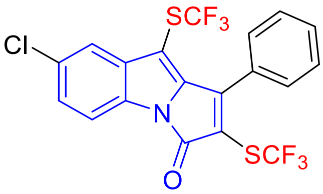

Yield: 83.5 mg, 70%; Red solid; m.p. 115-117 °C. ^1H NMR (400 MHz, $CDCl_3$) δ ppm 7.72 (d, *J* = 8.5 Hz, 1H), 7.56 (s, 1H), 7.53-7.44 (m, 5H), 7.36 (d, *J* = 8.5 Hz, 1H). ^{19}F NMR (377 MHz, $CDCl_3$) δ -39.77 (s, 3F), -40.87 (s, 3F). ^{13}C NMR (151 MHz, $CDCl_3$) δ ppm 160.9, 158.3, 144.2, 135.9, 132.4, 131.4, 130.7, 129.8, 129.0, 128.5, 128.2 (q, *J* = 314.0 Hz), 128.1 (q, *J* = 312.5 Hz), 127.7, 121.9, 120.2, 114.0, 105.2 (q, *J* = 2.7 Hz). IR (thin film) ν 2969, 2815, 2358, 1739, 1442, 1258, 1092, 1001, 795 cm^{-1}. HRMS (ESI-TOF): *m/z* Calculated for $C_{19}H_9ClF_6NOS_2$ $[M+H]^+$: 479.9713; Found: 479.9710.

7-Bromo-1-phenyl-2,9-bis(trifluoromethylthio)-3*H*-pyrrolo[1,2-*a*]indol-3-one (2i)

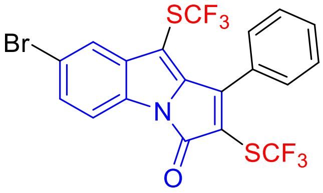

Yield: 95.6 mg, 73%; Red solid; m.p. 124-126 °C. ^{1}H NMR (400 MHz, $CDCl_3$) δ ppm 7.72 (s, 1H), 7.67 (d, *J* = 8.5 Hz, 1H), 7.51-7.47 (m, 6H). ^{19}F NMR (377 MHz, $CDCl_3$) δ ppm -39.75 (s, 3F), -40.86 (s, 3F). ^{13}C NMR (151 MHz, $CDCl_3$) δ ppm 160.9, 158.3, 144.0, 136.3, 132.8, 132.5, 131.4, 129.0, 128.5, 128.2 (q, *J* = 312.5 Hz), 128.1 (q, *J* = 312.5 Hz), 127.7, 124.9, 120.5, 118.1, 114.4, 105.1 (q, *J* = 2.6 Hz). IR (thin film) ν 2954, 2908, 1739, 1606, 1554, 1439, 1352, 1167, 1089, 1001 cm^{-1}. HRMS (ESI-TOF): *m/z* Calculated for $C_{19}H_9BrF_6NOS_2$ [M+H]$^+$: 523.9208; Found: 523.9206.

8-Methyl-1-phenyl-2,9-bis(trifluoromethylthio)-3*H*-pyrrolo[1,2-*a*]indol-3-one (2j)

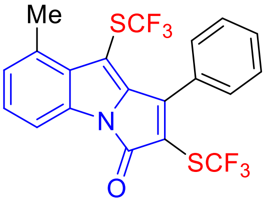

Yield: 54.3 mg, 47%; Red solid; m.p. 118-120 °C. ^{1}H NMR (400 MHz, $CDCl_3$) δ ppm 7.68 (d, *J* = 8.1 Hz, 1H), 7.52-7.35 (m, 5H), 7.24 (t, *J* = 7.8 Hz, 1H), 6.92 (d, *J* = 7.6 Hz, 1H), 2.64 (s, 3H). ^{19}F NMR (377 MHz, $CDCl_3$) δ ppm -40.07 (s, 3F), -42.61 (s, 3F). ^{13}C NMR (151 MHz, $CDCl_3$) δ ppm 161.1, 158.9, 143.8, 135.1, 134.7, 131.7, 130.8, 129.5, 129.0, 128.34, 128.33 (q, *J* = 327.7 Hz), 128.27, 128.0 (q, *J* = 321.1 Hz), 127.5, 119.9, 111.1, 106.1 (q, *J* = 3.0 Hz), 19.2. IR (thin film) *v* 2966, 2923, 2017, 1742, 1624, 1524, 1479, 1355, 1122, 1019 cm^{-1}. HRMS (ESI-TOF): *m/z* Calculated for $C_{20}H_{12}F_6NOS_2$ [M+H]$^+$: 460.0259; Found: 460.0258.

8-Methoxy-1-phenyl-2,9-bis(trifluoromethylthio)-3*H*-pyrrolo[1,2-*a*]indol-3-one (2k)

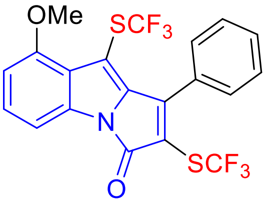

2k

Yield: 52.6 mg, 44%; Red solid; m.p. 130-132 °C. ^{1}H NMR (400 MHz, $CDCl_3$) δ 7.49-7.42 (m, 6H), 7.31 (d, *J* = 2.3 Hz, 1H), 6.79 (dd, *J* = 8.8, 2.3 Hz, 1H), 3.83 (s, 3H). ^{19}F

NMR (377 MHz, $CDCl_3$) δ -40.24 (s, 3F), -41.96 (s, 3F). ^{13}C NMR (101 MHz, $CDCl_3$) δ 160.4, 157.8, 155.3, 141.0, 134.7, 130.3, 129.9, 128.1, 127.4 (q, J = 313.1 Hz), 127.32 (q, J = 312.0 Hz), 127.27, 121.9, 118.0 (q, J = 2.2 Hz), 105.4, 105.1 (q, J = 2.6 Hz), 104.9, 54.5. **HRMS** (ESI-TOF): *m/z* Calculated for $C_{20}H_{12}F_6NO_2S_2$ $[M+H]^+$: 476.0208; Found: 476.0207.

Ethyl 3-oxo-1-phenyl-2,9-bis(trifluoromethylthio)-3*H*-pyrrolo[1,2-*a*]indole-8-carboxylate (2l)

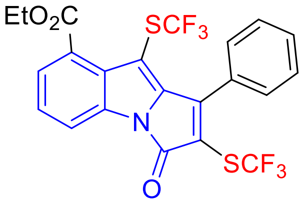

2l

Yield: 74.7 mg, 59%; Red solid; m.p. 92-94 ºC. **^{1}H NMR** (400 MHz, $CDCl_3$) δ ppm 7.96 (d, J = 5.7 Hz, 1H), 7.54–7.38 (m, 7H), 4.34 (q, J = 7.1 Hz, 2H), 1.31 (t, J = 7.2 Hz, 3H). ^{19}F NMR (377 MHz, $CDCl_3$) δ ppm -39.81 (s, 3F), -42.26 (s, 3F). **^{13}C NMR** (101 MHz, $CDCl_3$) δ ppm 165.4, 160.0, 157.9, 143.8, 133.6, 130.2, 130.0, 128.0, 127.9, 127.7, 127.4, 127.2 (q, J = 314.1 Hz), 127.1 (q, J = 312.0 Hz), 126.9, 124.5, 119.5, 114.5, 104.3 (q, J = 2.5 Hz), 60.7, 13.0. IR (thin film) *v* 3059, 2951, 2923, 1745, 1618, 1530, 1485, 1349, 1176, 1113 cm^{-1}. HRMS (ESI-TOF): *m/z* Calculated for $C_{22}H_{14}F_6NO_3S_2$ $[M+H]^+$: 518.0314; Found: 518.0315.

6-Methyl-1-phenyl-2,9-bis(trifluoromethylthio)-3*H*-pyrrolo[1,2-*a*]indol-3-one (2m)

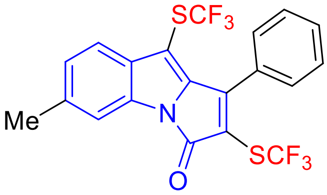

2m

Yield: 52.0 mg, 45%; Red solid; m.p. 84-86 ºC. ^{1}H NMR (400 MHz, $CDCl_3$) δ ppm 7.61 (s, 1H), 7.49-7.42 (m, 6H), 7.01 (d, J = 8.2 Hz, 1H), 2.40 (s, 3H). **^{19}F NMR** (377 MHz, $CDCl_3$) δ ppm -40.16 (s, 3F), -40.90 (s, 3F). **^{13}C NMR** (151 MHz, $CDCl_3$) δ ppm 161.3, 158.5, 142.4, 141.1, 134.6, 132.5, 131.1, 129.1, 128.42, 128.39 (q, J = 312.5 Hz), 128.35 (q, J = 312.5 Hz), 128.2, 126.2, 121.9, 118.9, 113.4, 106.6 (q, J = 2.5 Hz), 22.0. IR (thin film) *v* 2972, 2923, 2881, 2023, 1729, 1612, 1445, 1346, 1167, 1104 cm^{-1}. HRMS (ESI-TOF): *m/z* Calculated for $C_{20}H_{12}F_6NOS_2$ $[M+H]^+$: 460.0259; Found: 460.0258.

6-Methoxy-1-phenyl-2,9-bis(trifluoromethylthio)-3*H*-pyrrolo[1,2-*a*]indol-3-one (2n)

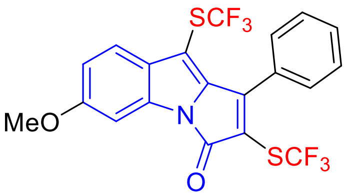

2n

Yield: 41.8 mg, 35%; Red solid; m.p. 98-100 ºC. ^{1}H NMR (400 MHz, $CDCl_3$) δ ppm 7.50-7.45 (m, 5H), 7.43 (d, *J* = 8.7 Hz, 1H), 7.30 (d, *J* = 2.3 Hz, 1H), 6.78 (dd, *J* = 8.8, 2.3 Hz, 1H), 3.83 (s, 3H). ^{19}F NMR (377 MHz, $CDCl_3$) δ ppm -40.40 (s, 3F), -40.86 (s, 3F). ^{13}C NMR (101 MHz, $CDCl_3$) δ ppm 162.4, 161.7, 158.8, 141.5, 135.9, 131.2, 129.1, 128.5 (q, *J* = 318.2 Hz), 128.4, 128.33 (q, *J* = 313.1Hz), 128.3, 128.2, 123.2, 117.5, 114.1, 107.1 (q, *J* = 2.6 Hz), 97.0, 56.0. IR (thin film) *v* 2936, 2367, 2017, 1757, 1615, 1491, 1349, 1270, 1092, 1034 cm^{-1}. HRMS (ESI-TOF): *m/z* Calculated for $C_{20}H_{12}F_6NO_2S_2$ $[M+H]^+$: 476.0208; Found: 476.0210.

6-Chloro-1-phenyl-2,9-bis(trifluoromethylthio)-3*H*-pyrrolo[1,2-*a*]indol-3-one (2o)

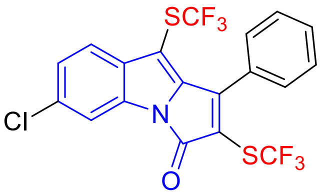

2o

Yield: 72.9 mg, 61%; Red solid; m.p. 108-110 ºC. ^{1}H NMR (400 MHz, $CDCl_3$) δ ppm 7.82 (d, *J* = 1.8 Hz, 1H), 7.54-7.43 (m, 6H), 7.20 (d, *J* = 1.9 Hz, 1H). ^{19}F NMR (377 MHz, $CDCl_3$) δ ppm -39.89 (s, 3F), -40.84 (s, 3F). ^{13}C NMR (101 MHz, $CDCl_3$) δ ppm 159.9, 157.4, 142.3, 135.1, 133.3, 132.2, 130.3, 128.0, 127.5, 127.2 (q, *J* = 314.1 Hz), 127.1 (q, *J* = 312.0 Hz), 126.8, 124.5, 122.0, 118.7, 112.5, 104.9 (q, *J* = 2.6 Hz). IR (thin film) *v* 3084, 2975, 2923, 2201, 2153, 1729, 1618, 1170, 1110, 1089 cm^{-1}. HRMS (ESI-TOF): *m/z* Calculated for $C_{19}H_9ClF_6NOS_2$ $[M+H]^+$: 479.9713; Found: 479.9710.

1-(*P*-Tolyl)-2,9-bis(trifluoromethylthio)-3*H*-pyrrolo[1,2-*a*]indol-3-one (2p)

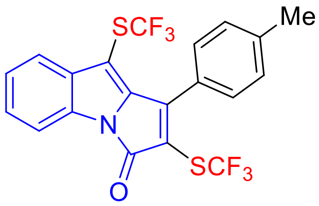

2p

Yield: 70.8 mg, 62%; Red solid; m.p. 106-108 °C. ^{1}H NMR (400 MHz, $CDCl_3$) δ ppm 7.90 (d, *J* = 8.0 Hz, 1H), 7.70 (d, *J* = 8.0 Hz, 1H), 7.51-7.48 (m, 3H), 7.38 (d, *J* = 7.8 Hz, 2H), 7.33 (d, *J* = 7.8 Hz, 1H), 2.51 (s, 3H). ^{19}F NMR (377 MHz, $CDCl_3$) δ ppm -40.09 (s, 3F), -40.80 (s, 3F). ^{13}C NMR (101 MHz, $CDCl_3$) δ ppm 161.4, 158.7, 143.2, 142.0, 134.8, 134.2, 129.7, 129.21, 129.18, 128.4 (q, *J* = 313.1 Hz), 126.9, 125.2, 124.8, 122.2, 118.8 (q, *J* = 2.4 Hz), 113.1, 106.2 (q, *J* = 2.6 Hz), 21.7. IR (thin film) *v* 2159, 1998, 1766, 1603, 1488, 1442, 1131, 1074, 1013, 750 cm^{-1}. HRMS (ESI-TOF): *m/z* Calculated for $C_{20}H_{12}F_6NOS_2$ $[M+H]^+$: 460.0259; Found: 460.0260.

9-Methyl-1-phenyl-2-(trifluoromethylthio)-3*H*-pyrrolo[1,2-*a*]indol-3-one (4a)

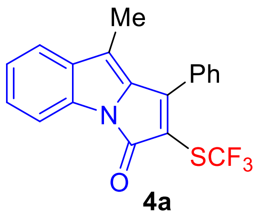

4a

Yield: 42.5 mg, 47%; Red solid; ^{1}H NMR (400 MHz, $CDCl_3$) δ ppm 7.71 (d, *J* = 8.0 Hz, 1H), 7.48 (s, 5H), 7.38-7.24 (m, 2H), 7.08 (t, *J* = 7.6 Hz, 1H), 2.04 (s, 3H). ^{19}F NMR (377 MHz, $CDCl_3$) δ ppm -41.00 (s, 3F). ^{13}C NMR (101 MHz, $CDCl_3$) δ ppm 160.6, 157.6, 134.2, 134.1, 133.5, 129.7, 128.8, 128.2, 127.73, 127.66, 127.6 (q, *J* = 313.1), 123.6, 122.6, 120.5, 114.6, 111.9, 9.0. IR (thin film) *v* 2966, 2917, 2364, 2186, 1953, 1726, 1587, 1536, 1137, 1092, 711 cm^{-1}. HRMS (ESI-TOF): *m/z* Calculated for $C_{19}H_{13}F_3NOS$ $[M+H]^+$: 360.0664; Found: 360.0665.

1,9-Diphenyl-2-(trifluoromethylthio)-3*H*-pyrrolo[1,2-*a*]indol-3-one (4b)

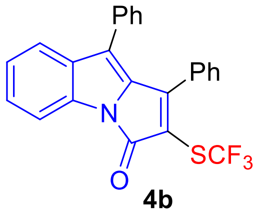

4b

Yield: 59.7 mg, 57%; Red solid; m.p. 132-134 °C. ^{1}H NMR (400 MHz, $CDCl_3$) δ 7.82 (d, *J* = 8.0 Hz, 1H), 7.41 (d, *J* = 7.9 Hz, 1H), 7.35 (t, *J* = 7.6 Hz, 1H), 7.25 (t, *J* = 7.3 Hz, 1H), 7.18 (s, 2H), 7.13 (s, 1H), 7.12-7.03 (m, 7H). ^{19}F NMR (377 MHz, $CDCl_3$) δ -40.75 (s, 3F). ^{13}C NMR (101 MHz, $CDCl_3$) δ 160.8, 157.7, 134.1, 133.3, 132.0, 129.2, 128.7, 128.3, 128.2, 127.7, 127.5, 127.2, 127.2, 126.6 (q, *J* = 313.1Hz), 123.0, 121.8, 115.1, 112.1. HRMS (ESI-TOF): *m/z* Calculated for $C_{24}H_{15}F_3NOS$ $[M+H]^+$: 422.0821; Found: 422.0821.

3-Oxo-1-phenyl-2-(trifluoromethylthio)-3*H*-pyrrolo[1,2-*a*]indole-9-carbonitrile (4c)

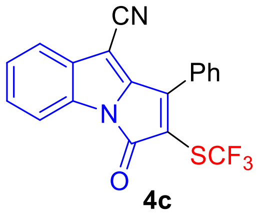

4c

Yield: 33.1 mg, 36%; Red solid; m.p. 124-126 ºC. ^{1}H NMR (400 MHz, $CDCl_3$) δ ppm 7.79 (d, *J* = 8.1 Hz, 1H), 7.70 (d, *J* = 7.1 Hz, 2H), 7.58-7.53 (m, 4H), 7.43 (t, *J* = 7.8 Hz, 1H), 7.25 (t, *J* = 7.7 Hz, 1H). ^{19}F NMR (377 MHz, $CDCl_3$) δ ppm -39.64 (s, 3H). ^{13}C NMR (101 MHz, $CDCl_3$) δ ppm 160.0, 155.1, 141.9, 132.4, 131.6, 130.5, 129.1, 128.0, 127.2 (q, *J* = 314.1 Hz), 126.3, 124.3, 120.8, 117.2 (d, *J* = 2.0 Hz), 112.3, 111.2, 92.8. IR (thin film) *v* 2960, 2917, 2848, 2234, 2086, 1736, 1612, 1545, 1173, 1128, 1002 cm^{-1}. HRMS (ESI-TOF): *m/z* Calculated for $C_{19}H_{10}F_3N_2OS$ [M+H]$^+$: 371.0460; Found: 371.0461.

9-Acetyl-1-phenyl-2-(trifluoromethylthio)-3*H*-pyrrolo[1,2-*a*]indol-3-one (4d)

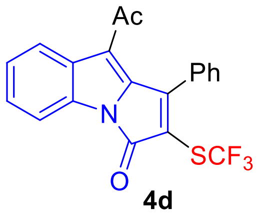

4d

Yield: 36.9 mg, 38%; Red solid; m.p. 128-130 ºC. ^{1}H NMR (400 MHz, $CDCl_3$) δ 7.72 (d, *J* = 8.1 Hz, 1H), 7.66 (d, *J* = 8.0 Hz, 1H), 7.54-7.38 (m, 5H), 7.32 (t, *J* = 7.8 Hz, 1H), 7.13 (t, *J* = 7.7 Hz, 1H), 1.78 (s, 3H). ^{19}F NMR (377 MHz, $CDCl_3$) δ -40.04 (s, 3F). ^{13}C NMR (101 MHz, $CDCl_3$) δ 194.8, 160.2, 157.3, 136.8, 133.1, 130.2, 129.7, 129.3, 128.4, 128.1, 127.4, 127.3 (q, *J*= 313.1Hz), 123.8, 122.7, 122.5, 118.3, 111.7, 30.4. IR (thin film) *v* 3072, 2364, 2032, 1968, 1729, 1672, 1606, 1557, 1473, 1448, 1110, 904 cm^{-1}. HRMS (ESI-TOF): *m/z* Calculated for $C_{20}H_{13}F_3NO_2S$ [M+H]$^+$: 388.0614; Found: 388.0611.

4. Mechanistic experiments

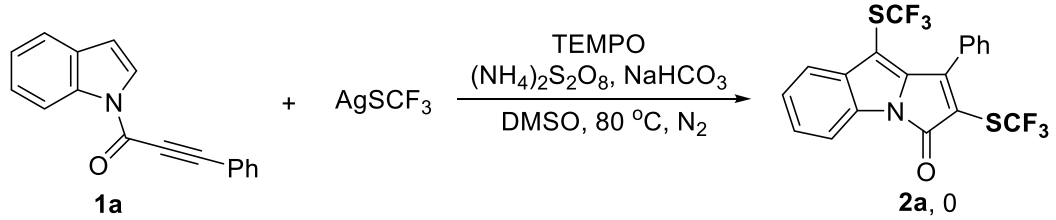

A 25 mL Schlenk tube equipped with a magnetic stir bar was charged with **1a** (61.3 mg, 0.25 mmol, 1.0 equiv), $AgSCF_3$ (156.7 mg, 0.75 mmol, 3.0 equiv), $(NH_4)_2S_2O_8$ (171.2 mg, 0.75 mmol, 3.0 equiv), $NaHCO_3$ (21.0 mg, 0.25 mmol, 1.0 equiv), and TEMPO (195.3 mg, 1.25 mmol, 5.0 equiv). The tube was sealed with a septum, evacuated and backfilled with nitrogen three times. Then DMSO (5.0 mL) was added by a syringe. The mixture was stirred at 80 °C for 12 h. The internal standard $PhCF_3$ (61.4 μL, 0.5 mmol) was added, and the solution was then analyzed by ^{19}F NMR spectroscopy. The desired product **2a** was not formed.

^{19}F NMR spectrum of the reaction mixture

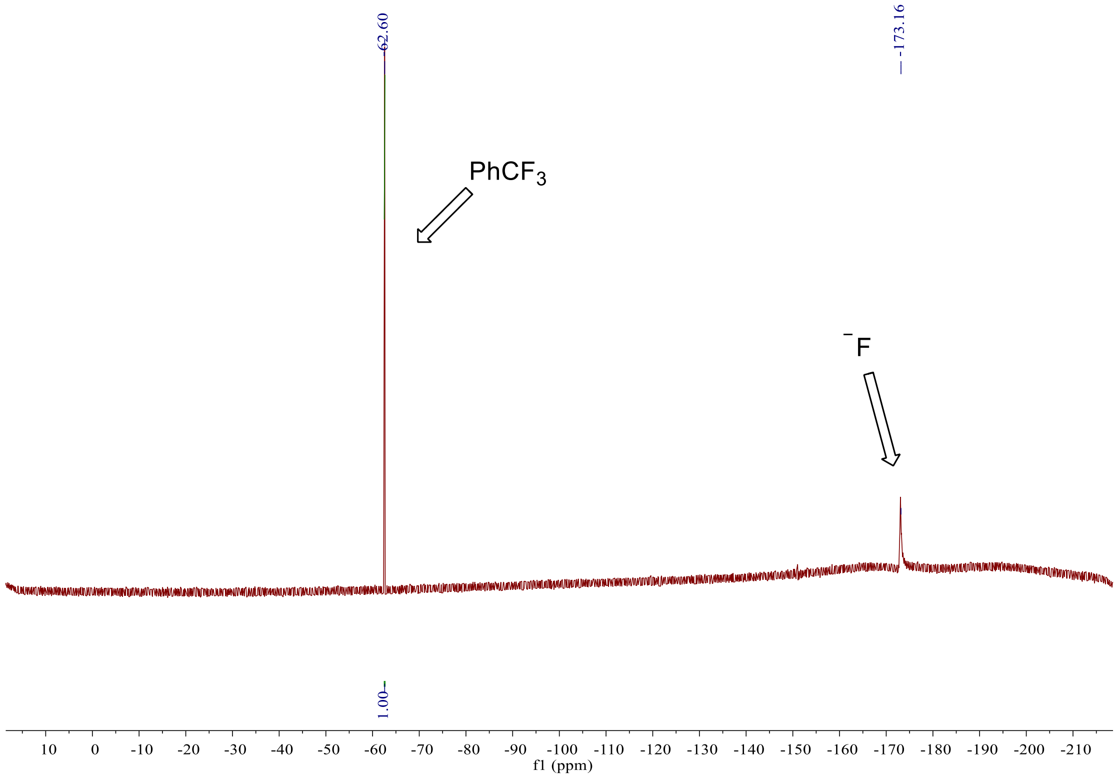

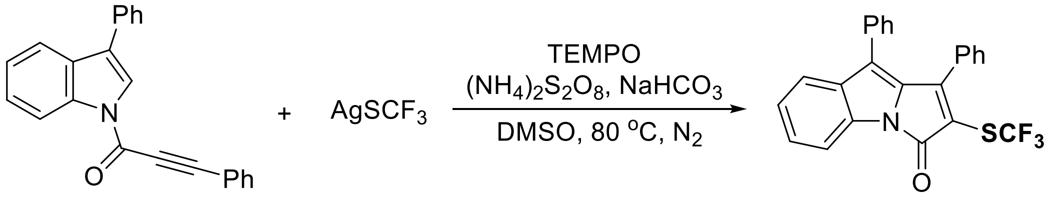

3b **4b**, trace

A 25 mL Schlenk tube equipped with a magnetic stir bar was charged with **3b** (80.4 mg, 0.25 mmol, 1.0 equiv), $AgSCF_3$ (156.7 mg, 0.75 mmol, 3.0 equiv), $(NH_4)_2S_2O_8$ (171.2 mg, 0.75 mmol, 3.0 equiv), $NaHCO_3$ (21.0 mg, 0.25 mmol, 1.0 equiv), and TEMPO (195.3 mg, 1.25 mmol, 5.0 equiv). The tube was sealed with a septum, evacuated and backfilled with nitrogen three times. Then DMSO (5.0 mL) was added by a syringe. The mixture was stirred at 80 °C for 12 h. The internal standard $PhCF_3$ (30.7 μL, 0.25 mmol) was added, and the solution was then analyzed by ^{19}F NMR spectroscopy. Only trace of the desired product **4b** was detected.

^{19}F NMR spectrum of the reaction mixture

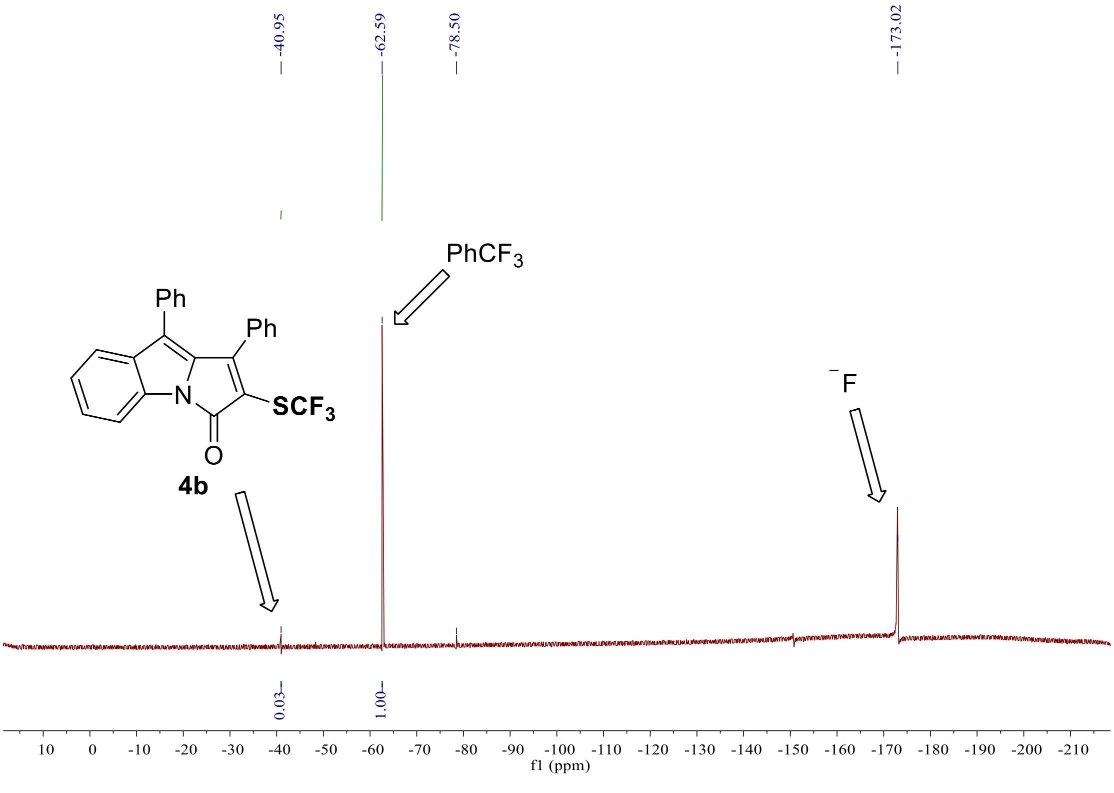

5. References

1. Yoon, H.; Rölz, M.; Landau, F.; Lautens, M. *Angew. Chem. Int. Ed.* **2017**, *56*, 10920-10923.

2. Wang, C.-S.; Roisnel, T.; Dixneuf, P. H.; Soulé, J.-F. *Adv. Synth. Catal.* **2019**, *361*, 445-405.

6. ORTEP Drawing of the X-Ray Crystallographic Structure of Compound 2a

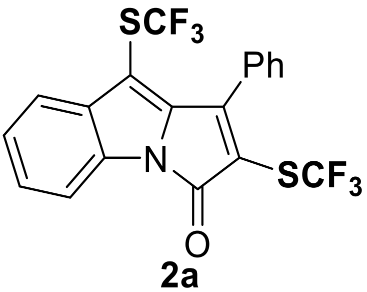

2a

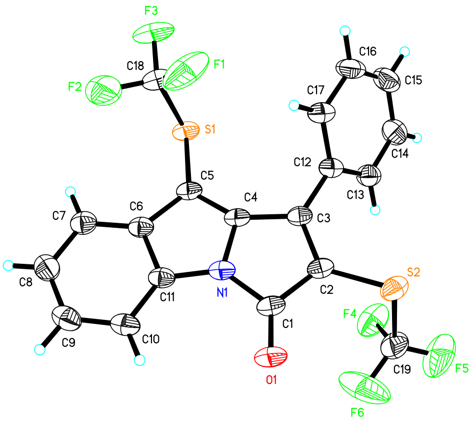

The crystal structure has been deposited at the Cambridge Crystallographic Date Center and allocated the deposition numbers CCDC 1968129.

This data can be obtained free of charge from the Cambridge Crystallographic Date Center via www.ccdc.cam.ac.uk/data_request/cif

7. Copies of ^{1}H and ^{13}C NMR spectra for the products

^{1}**H NMR** spectrum of **1a** in CDCl3

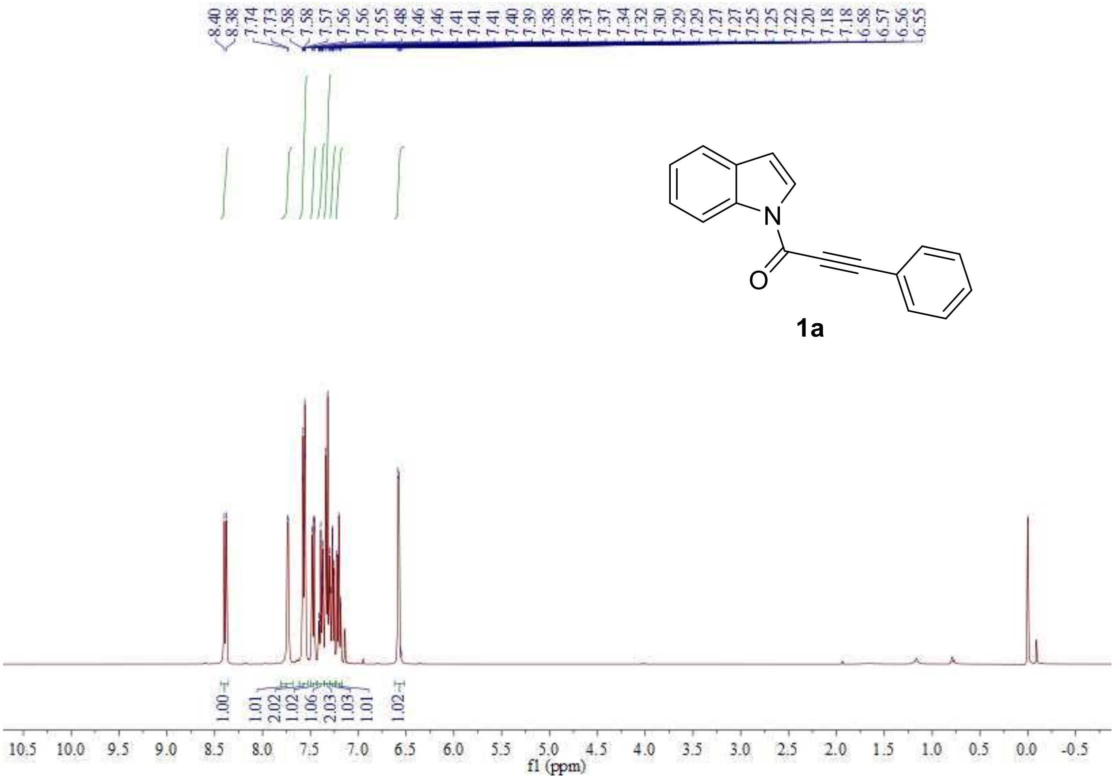

^{13}**C NMR** spectrum of **1a** in CDCl3

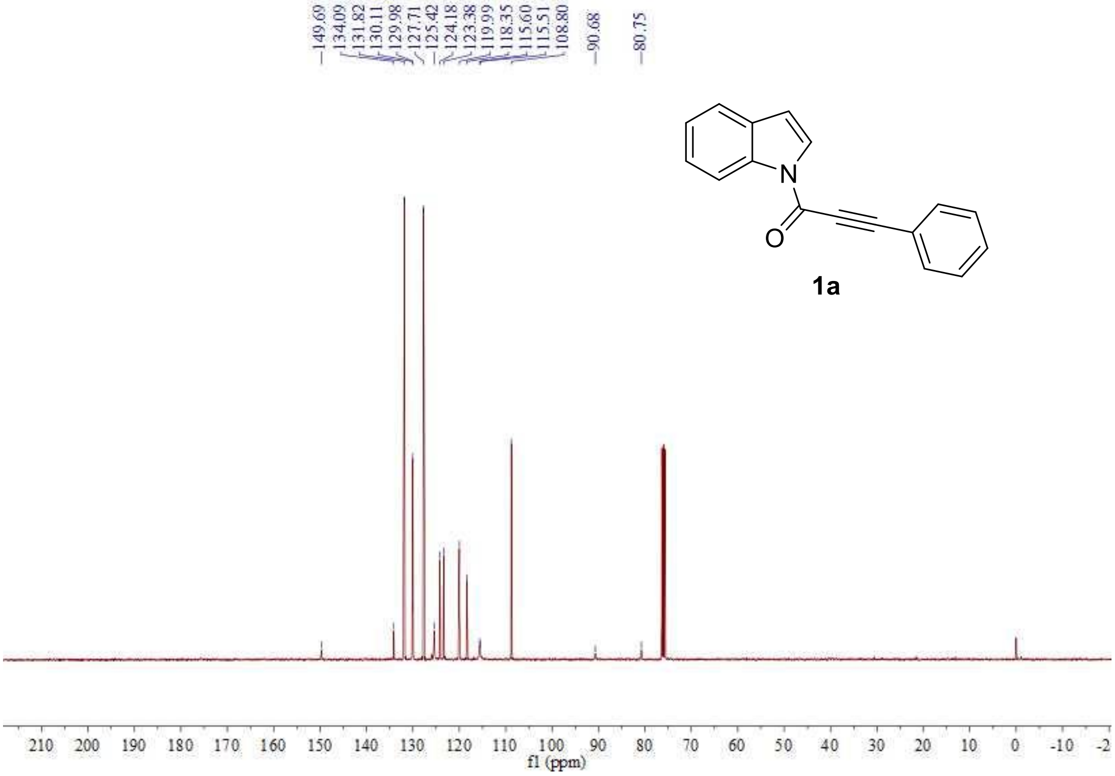

^{1}**H NMR** spectrum of **1b** in $CDCl_3$

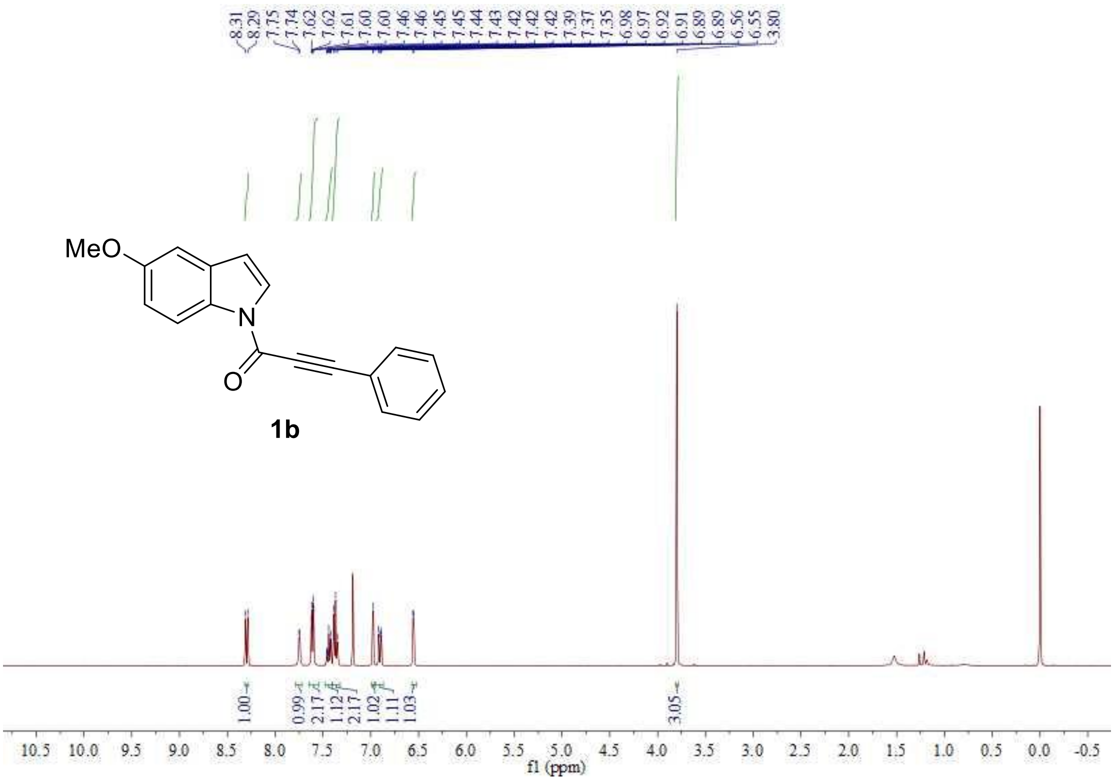

^{13}**C NMR** spectrum of **1b** in $CDCl_3$

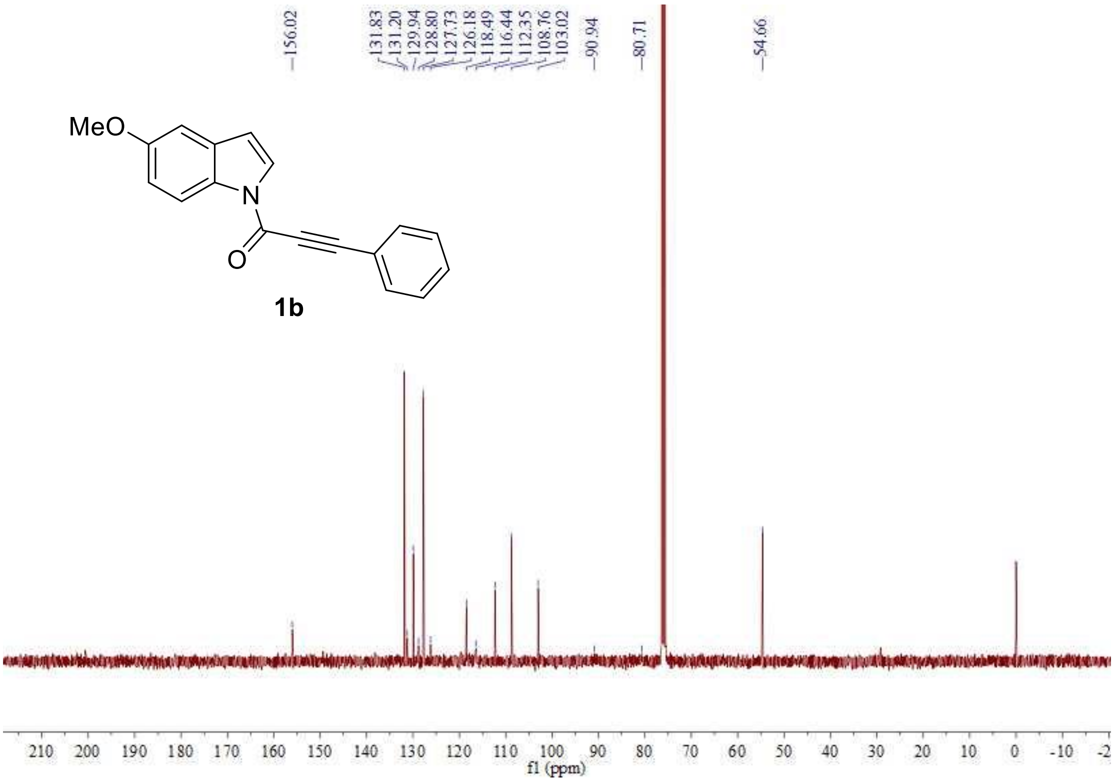

[1]**H NMR** spectrum of **1c** in $CDCl_3$

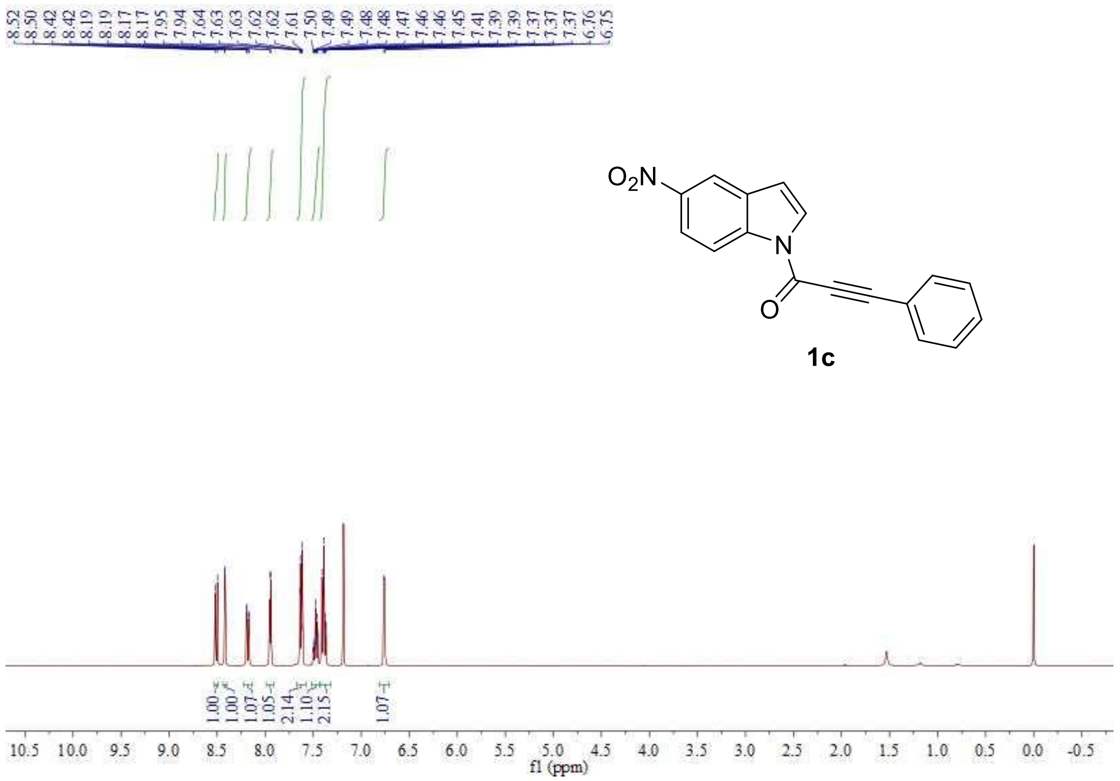

[13]**C NMR** spectrum of **1c** in $CDCl_3$

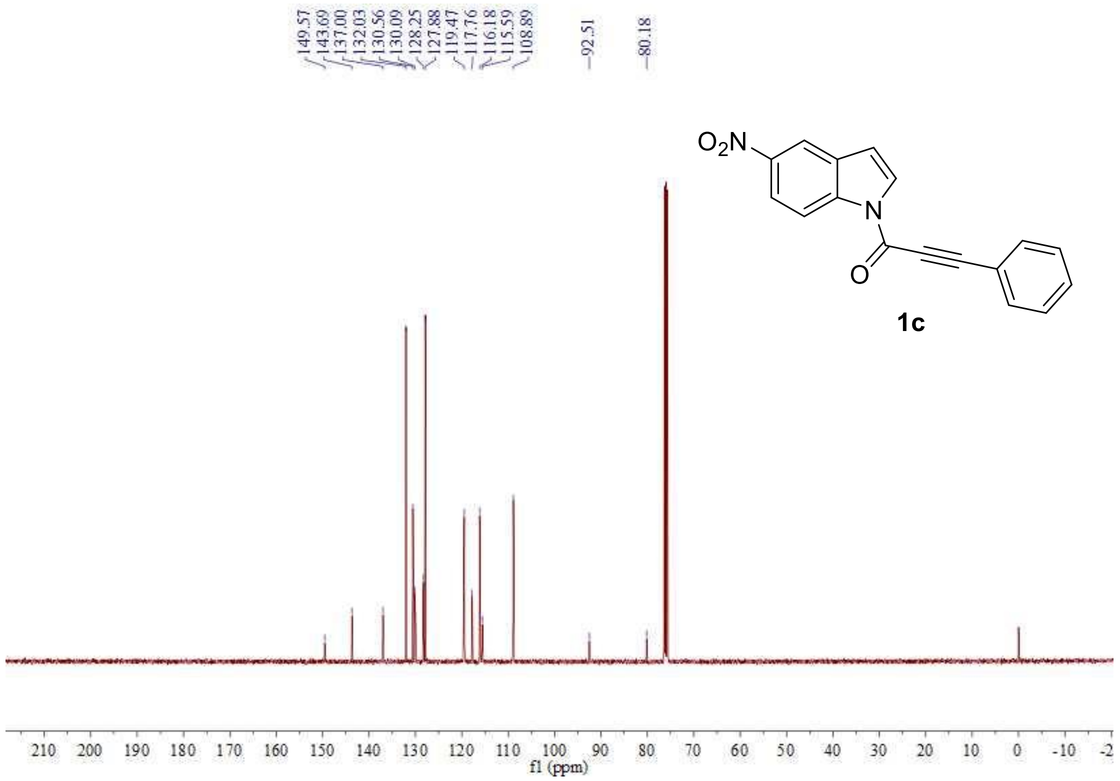

^{1}H NMR spectrum of **1d** in $CDCl_3$

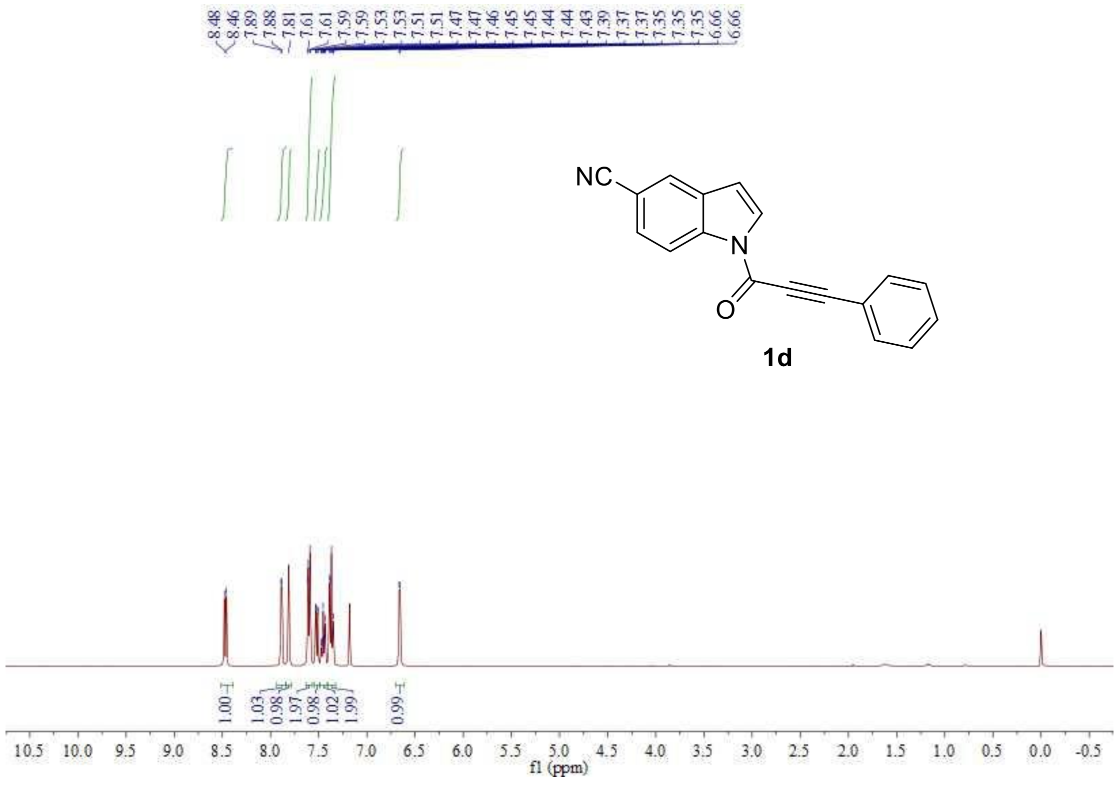

^{13}C NMR spectrum of **1d** in $CDCl_3$

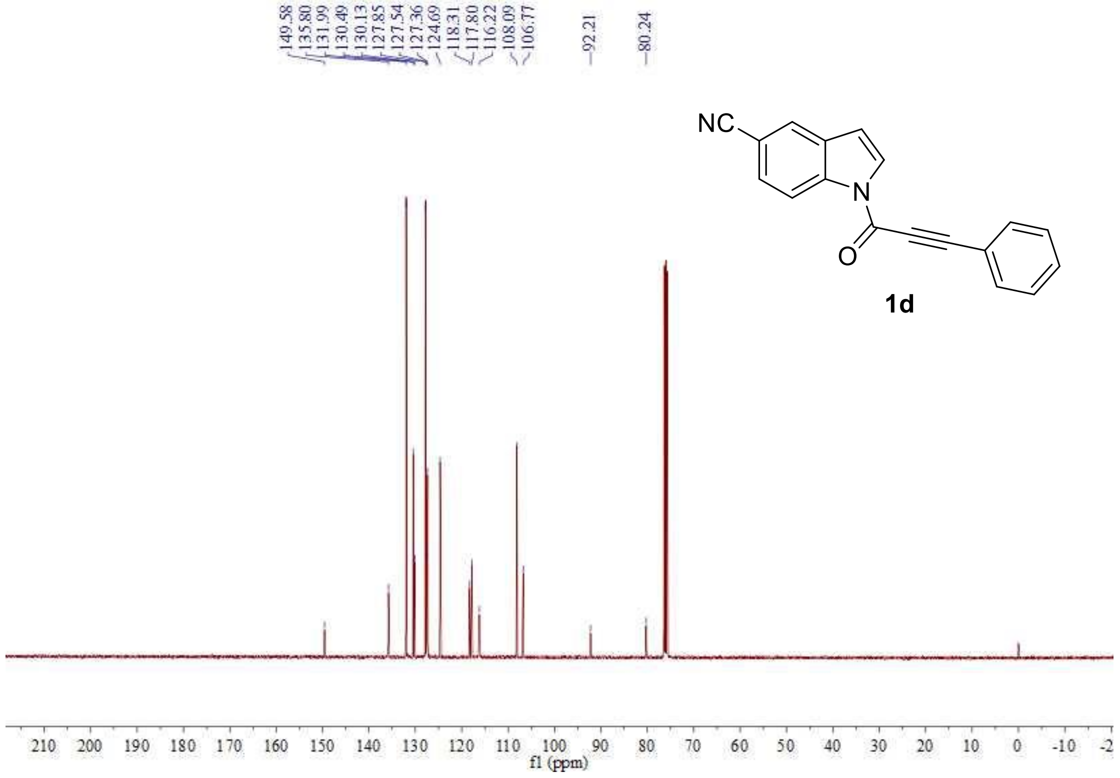

^{1}H NMR spectrum of **1e** in $CDCl_3$

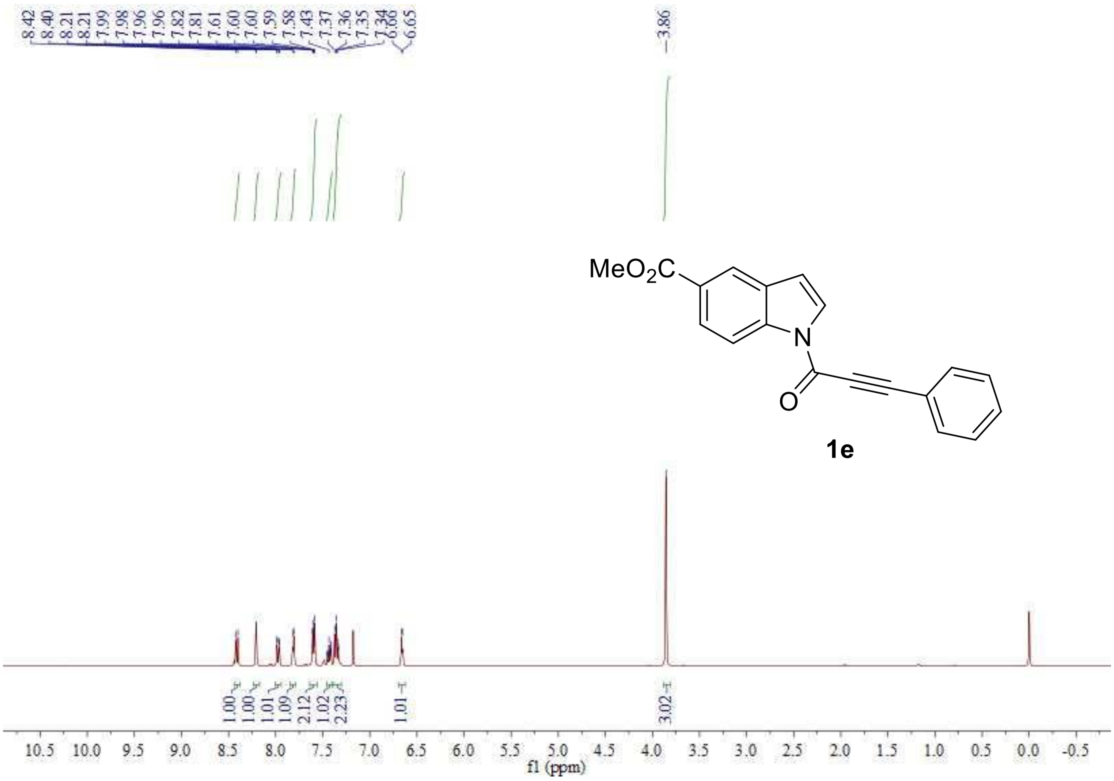

^{13}C NMR spectrum of **1e** in $CDCl_3$

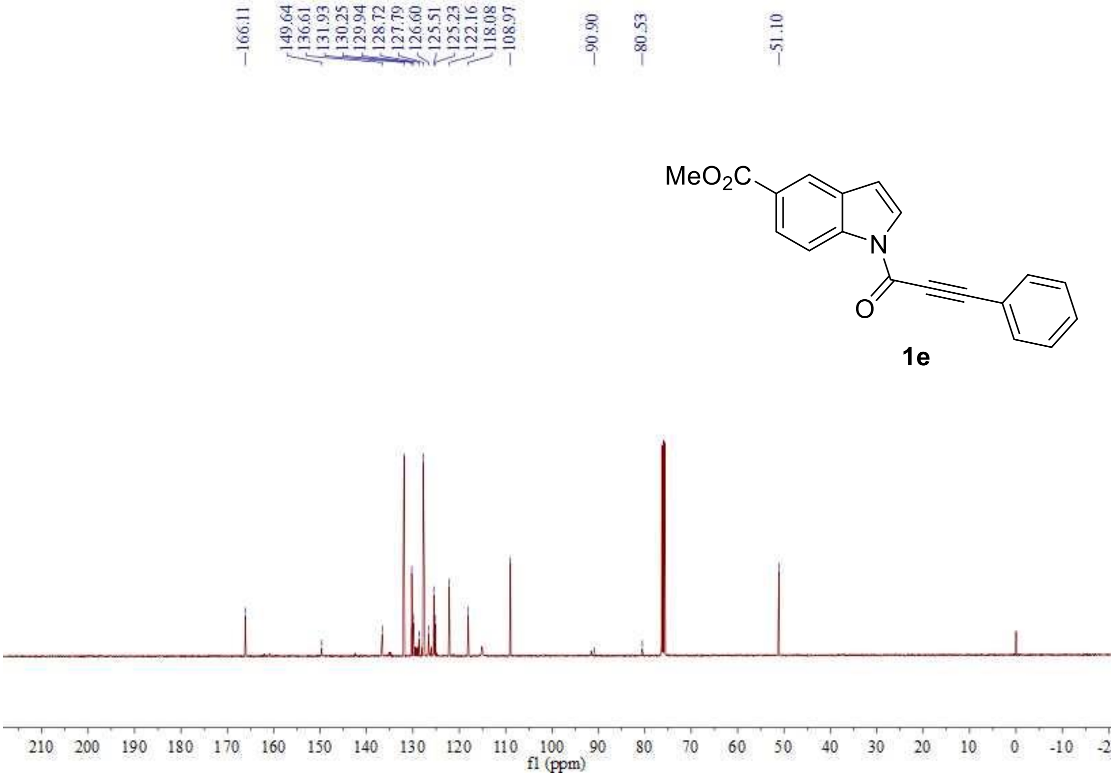

^{1}H NMR spectrum of **1f** in $CDCl_3$

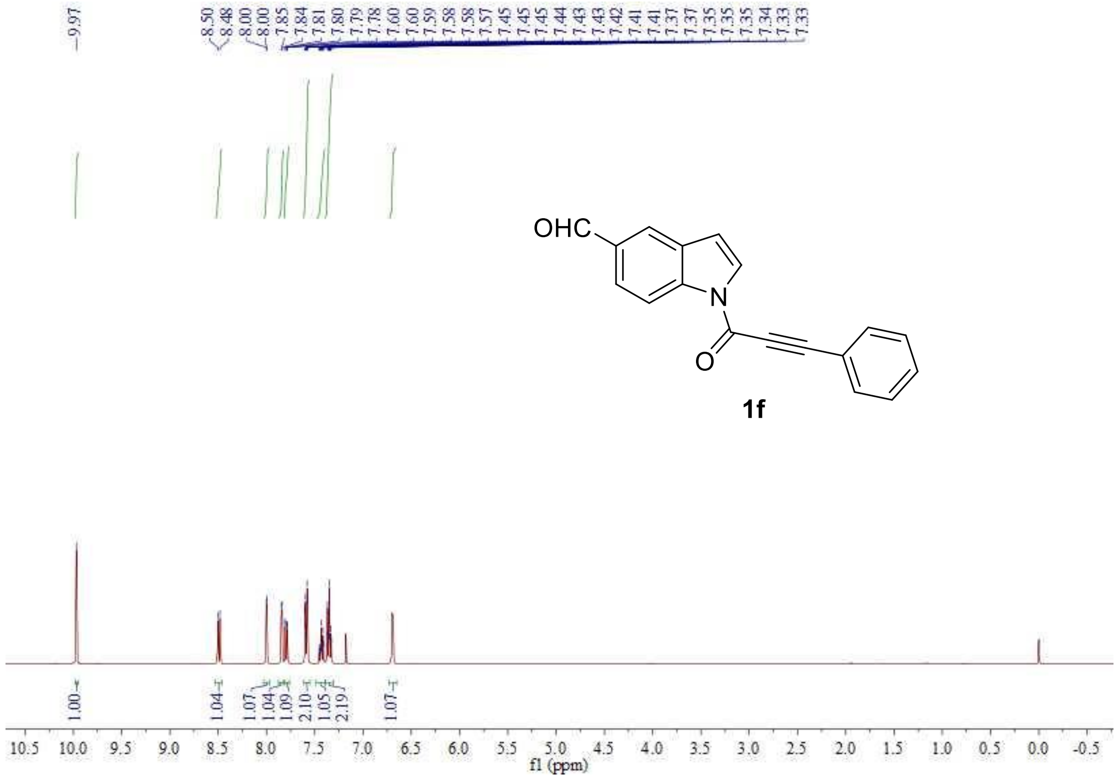

^{13}C NMR spectrum of **1f** in $CDCl_3$

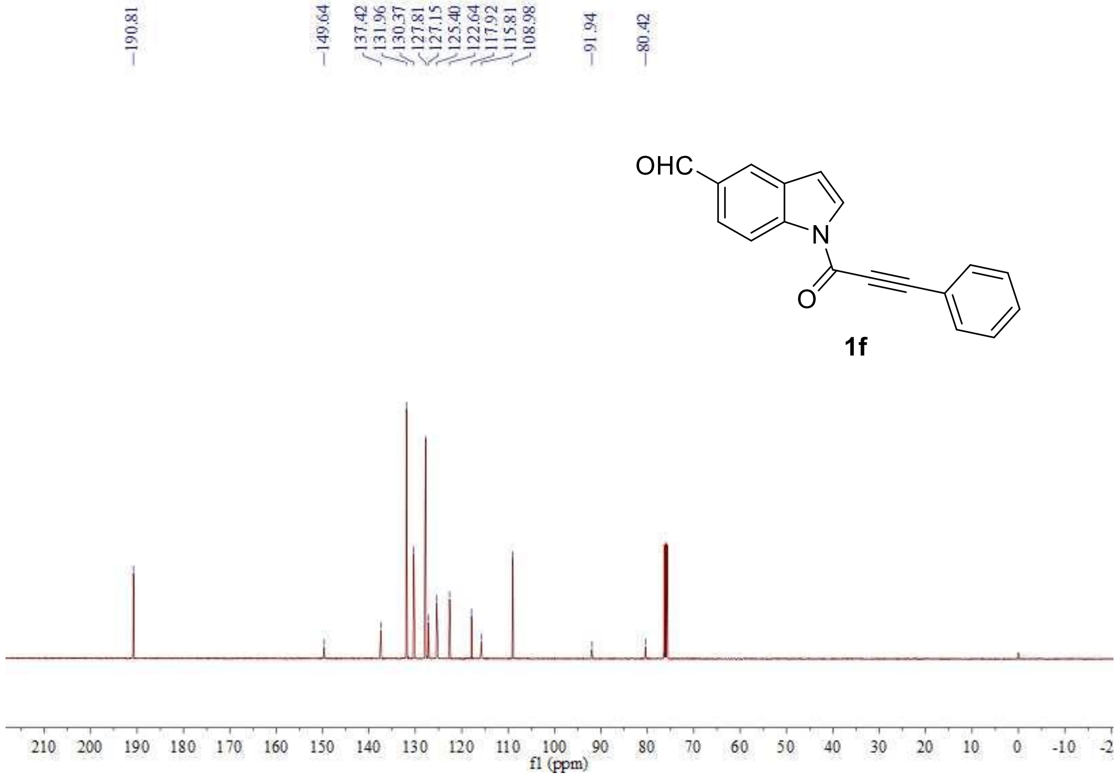

^{1}H NMR spectrum of **1g** in CDCl$_3$

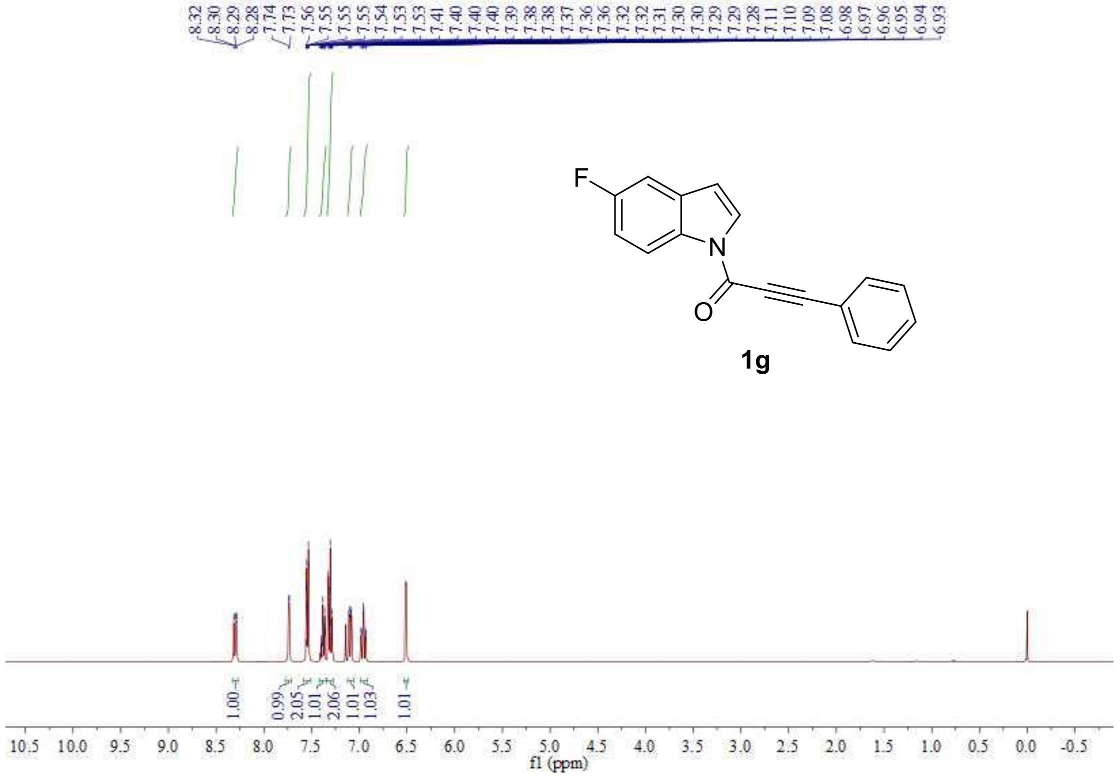

^{13}C NMR spectrum of **1g** in CDCl$_3$

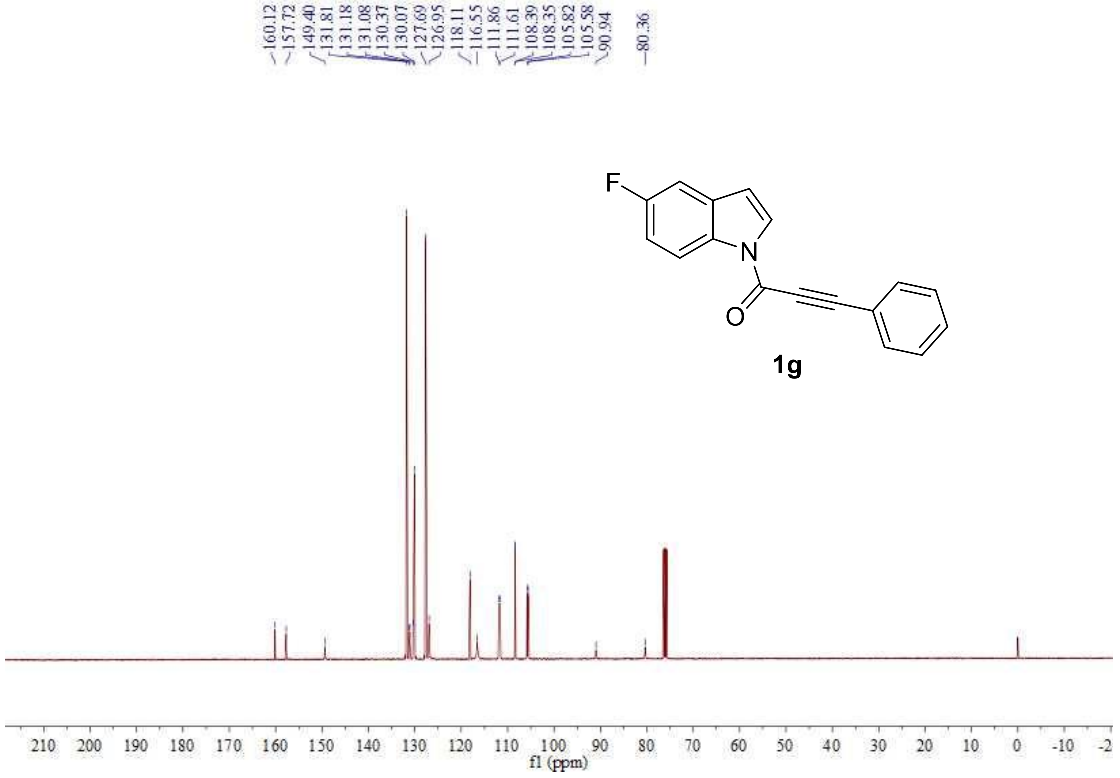

^{1}H NMR spectrum of **1h** in $CDCl_3$

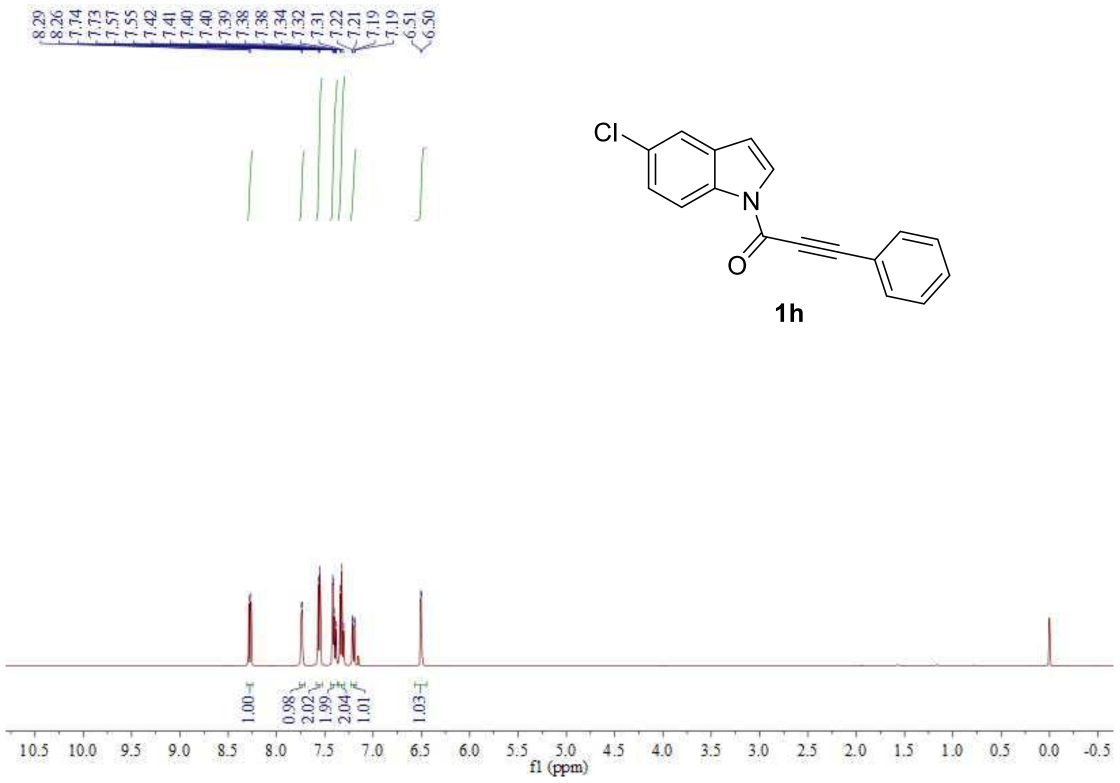

^{13}C NMR spectrum of **1h** in $CDCl_3$

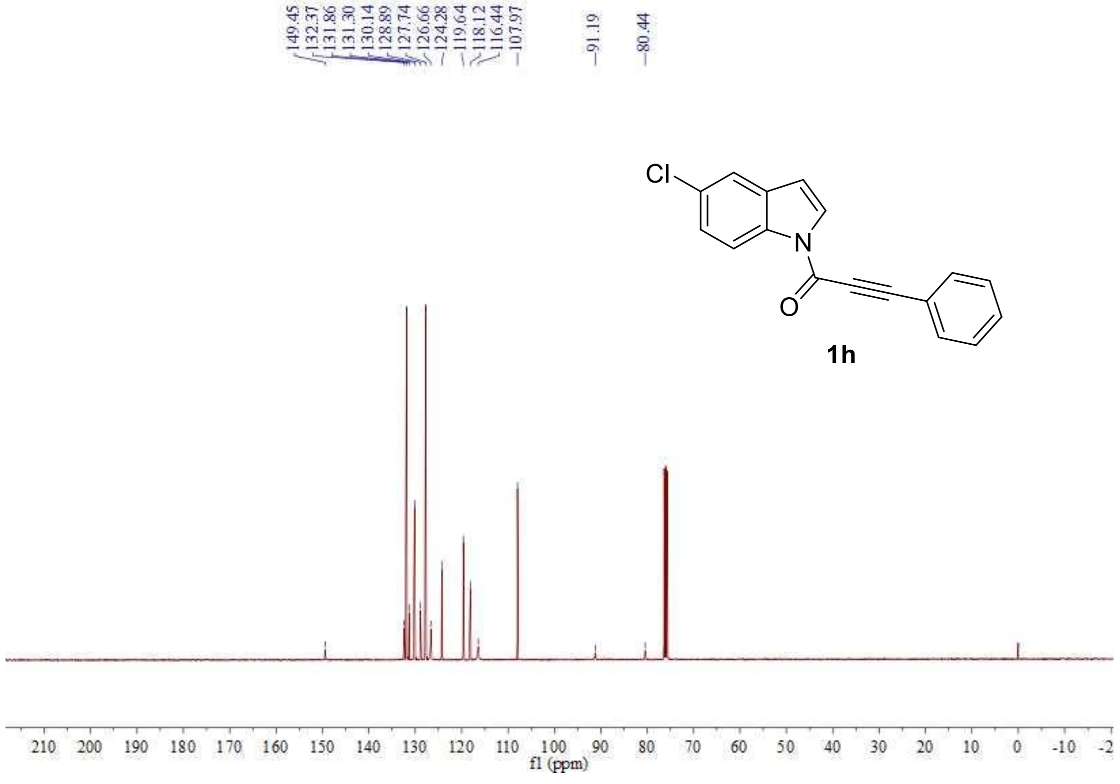

[1]**H NMR** spectrum of **1i** in $CDCl_3$

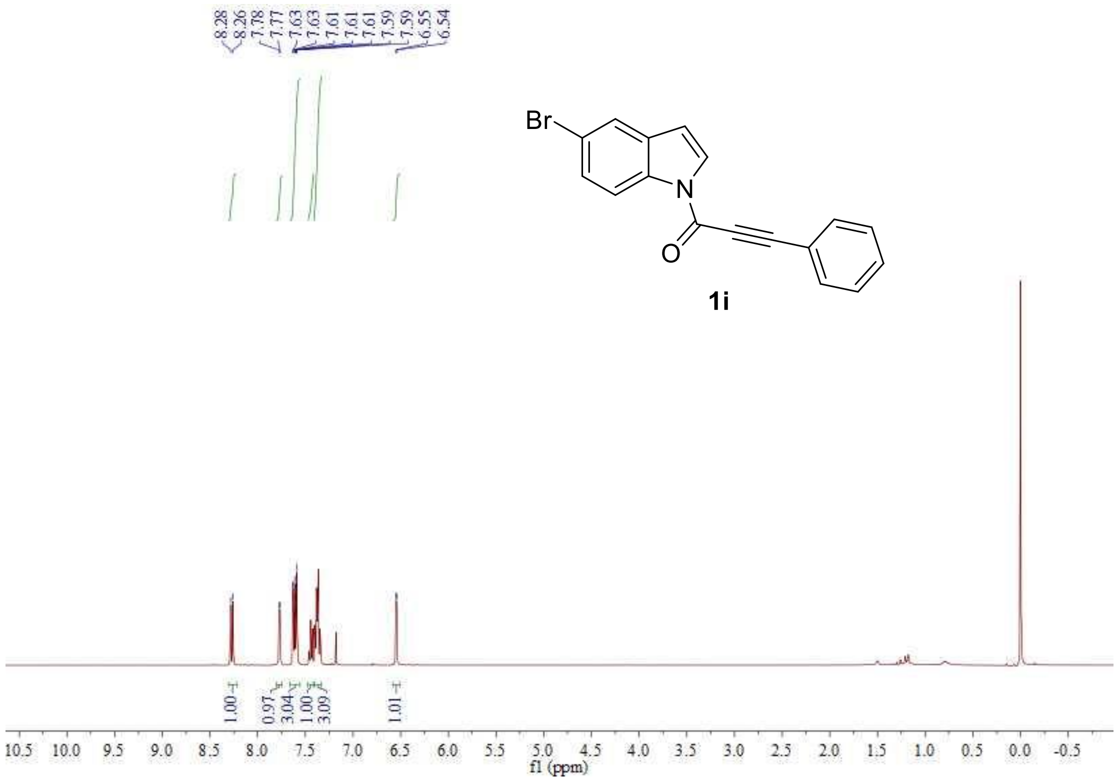

[13]**C NMR** spectrum of **1i** in $CDCl_3$

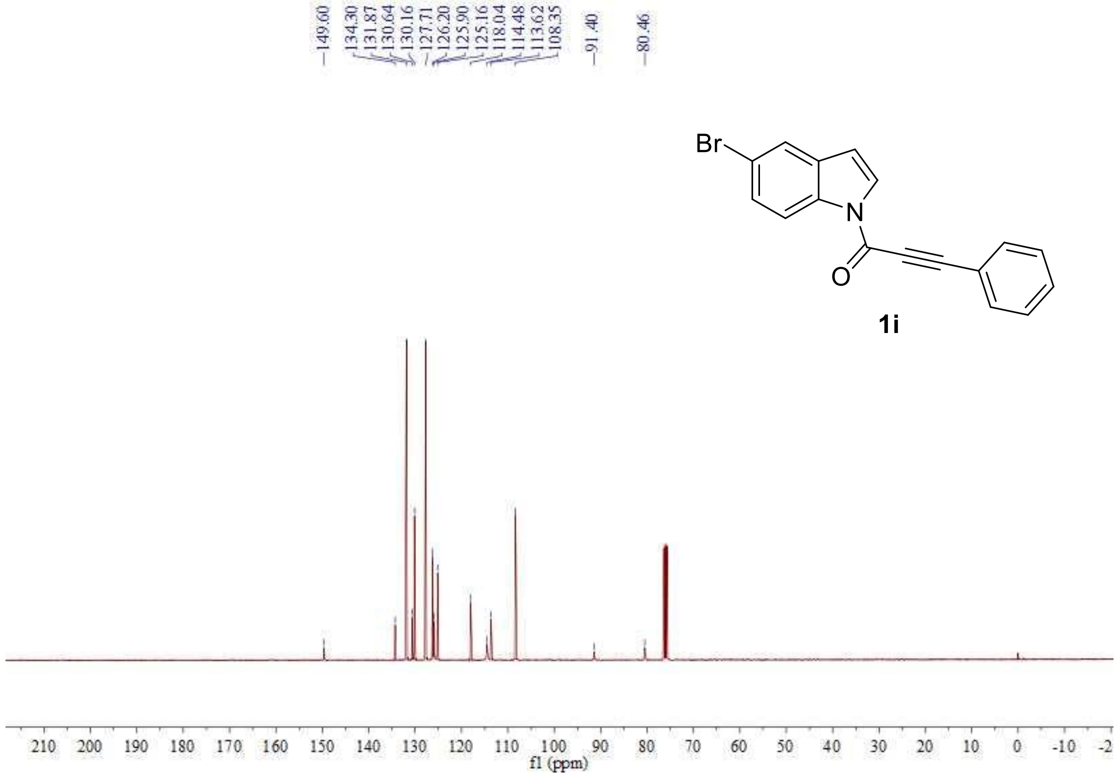

^{1}H NMR spectrum of **1j** in $CDCl_3$

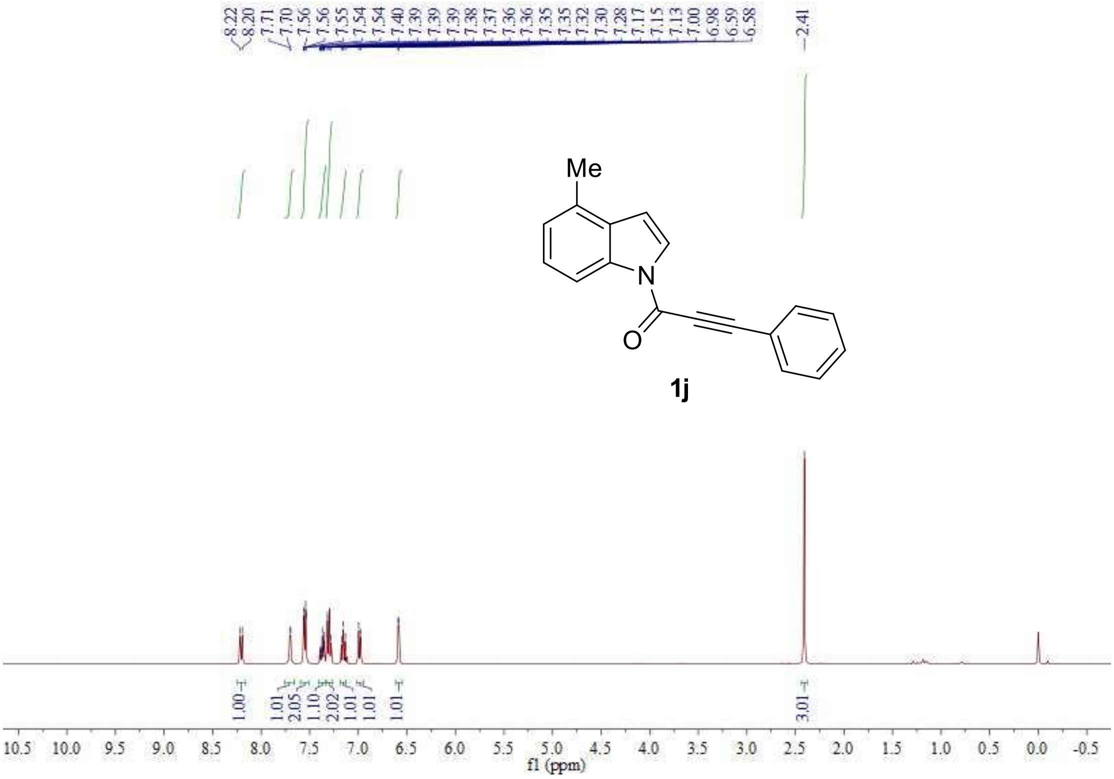

^{13}C NMR spectrum of **1j** in $CDCl_3$

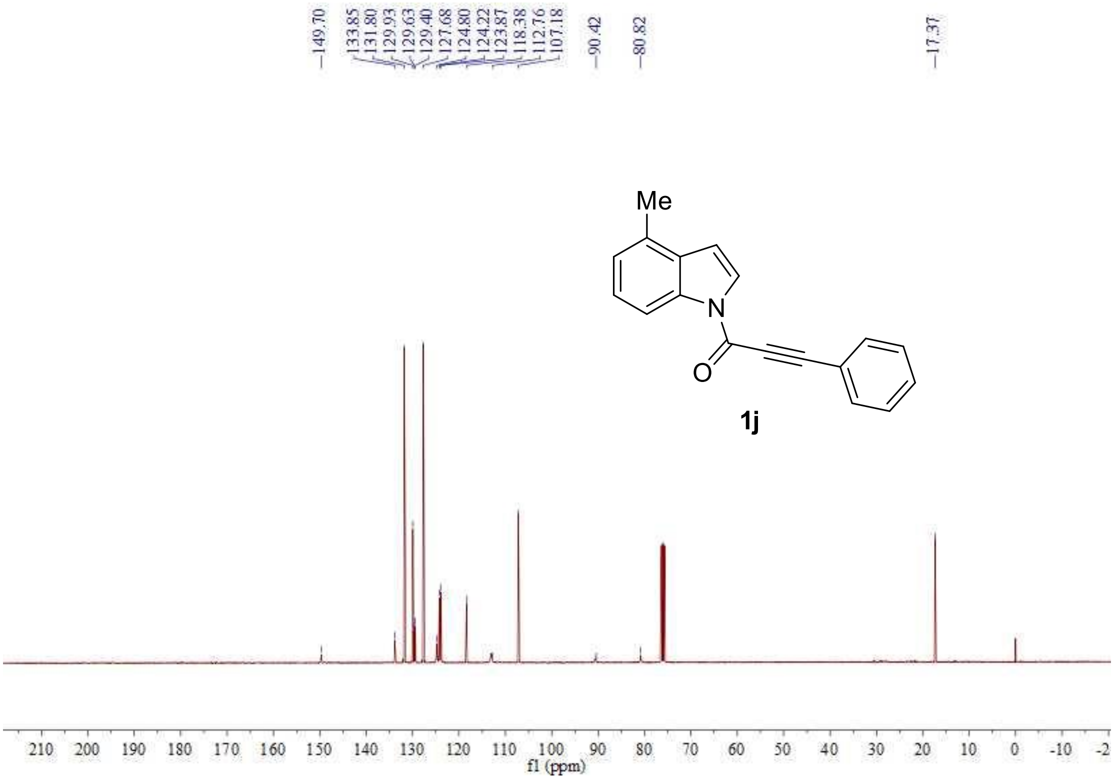

^{1}H NMR spectrum of **1k** in $CDCl_3$

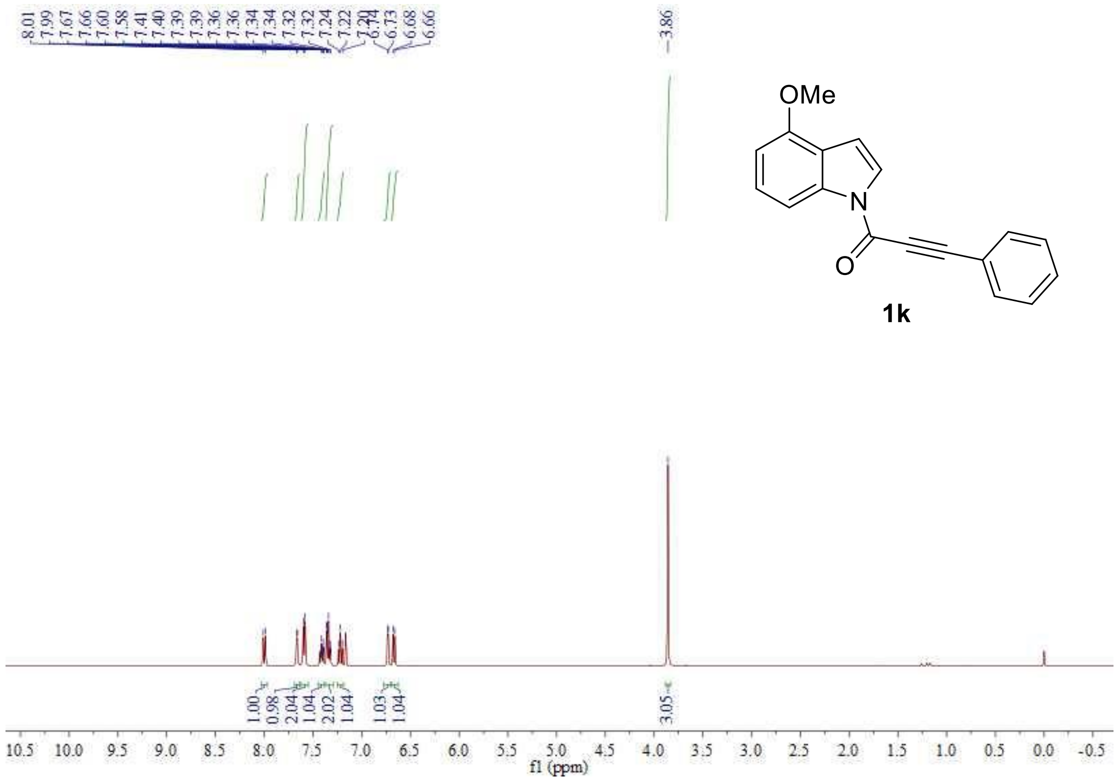

^{13}C NMR spectrum of **1k** in $CDCl_3$

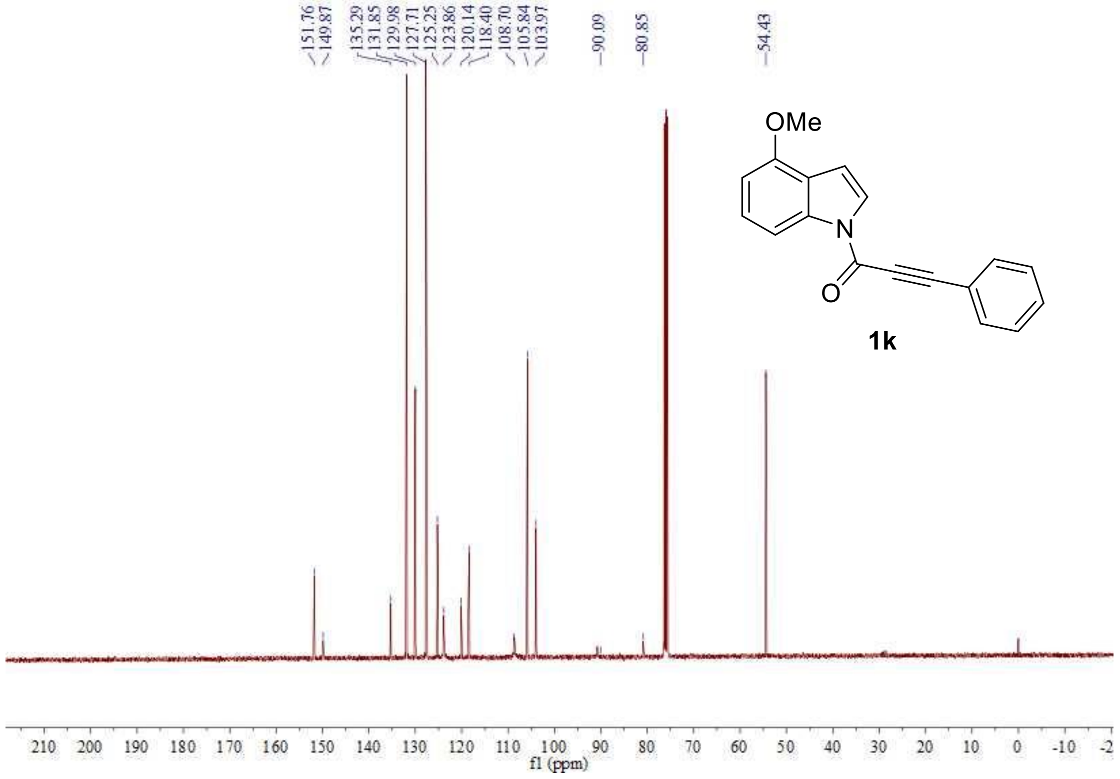

^{1}H NMR spectrum of **1l** in $CDCl_3$

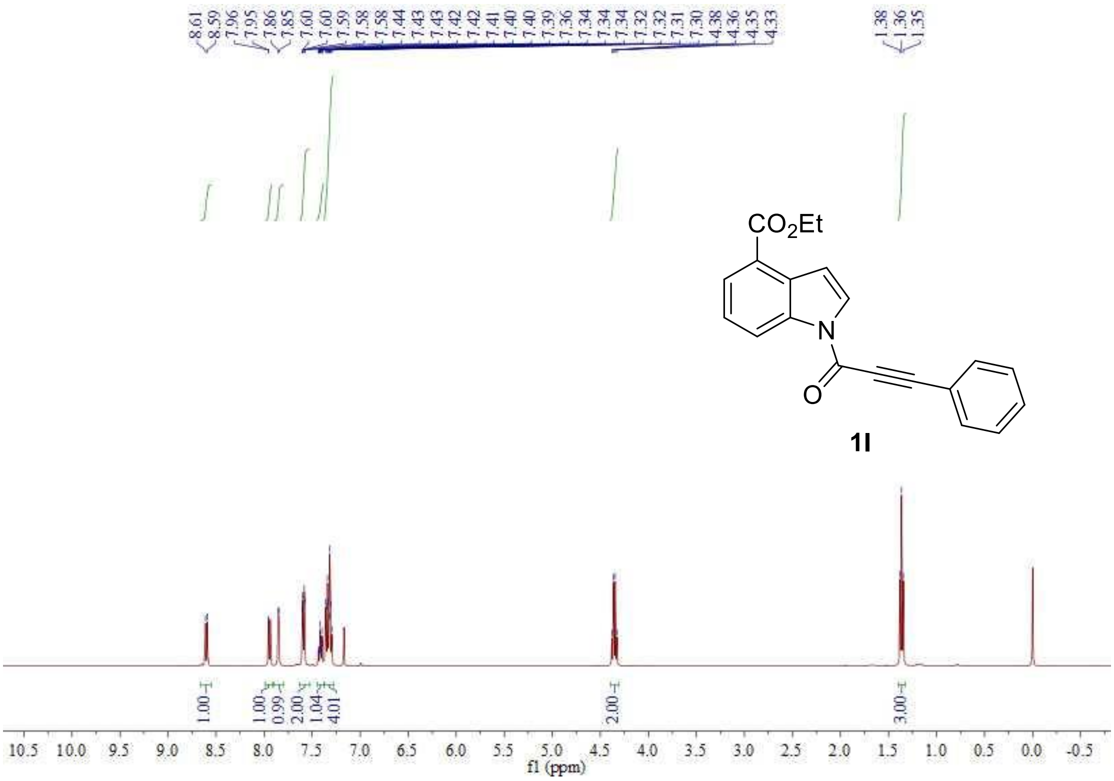

^{13}C NMR spectrum of **1l** in $CDCl_3$

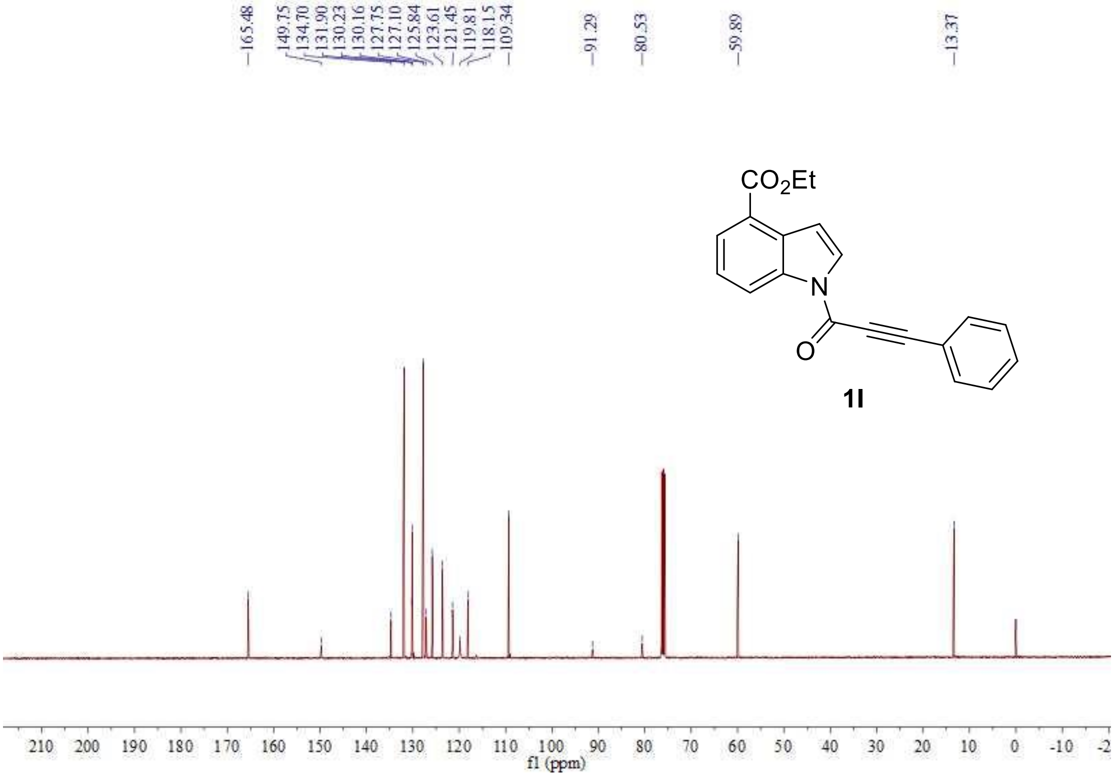

¹H NMR spectrum of **1m** in CDCl3

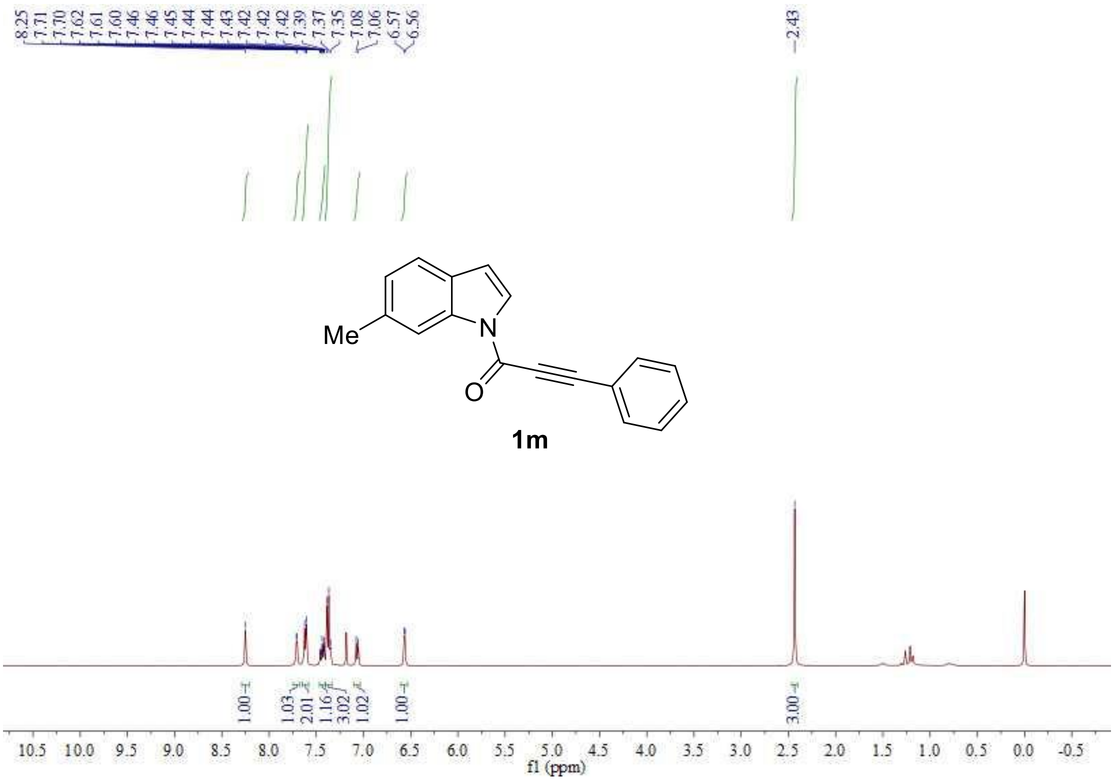

¹³C NMR spectrum of **1m** in CDCl3

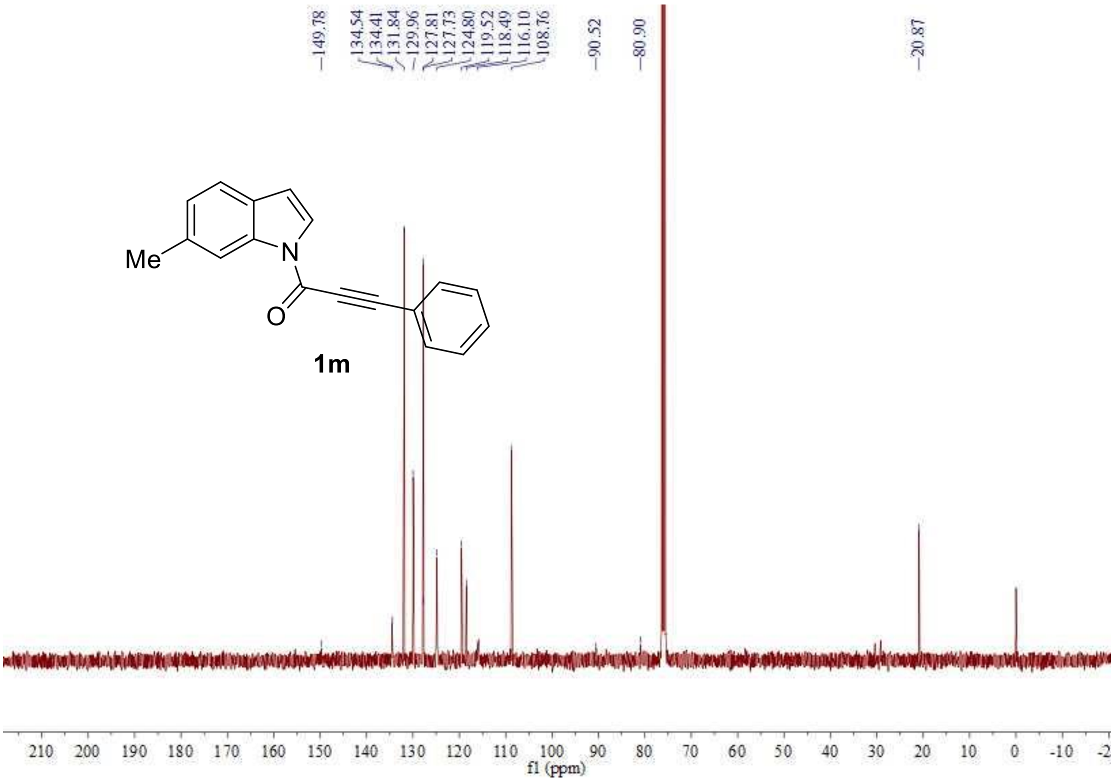

^{1}H NMR spectrum of 1n in CDCl3

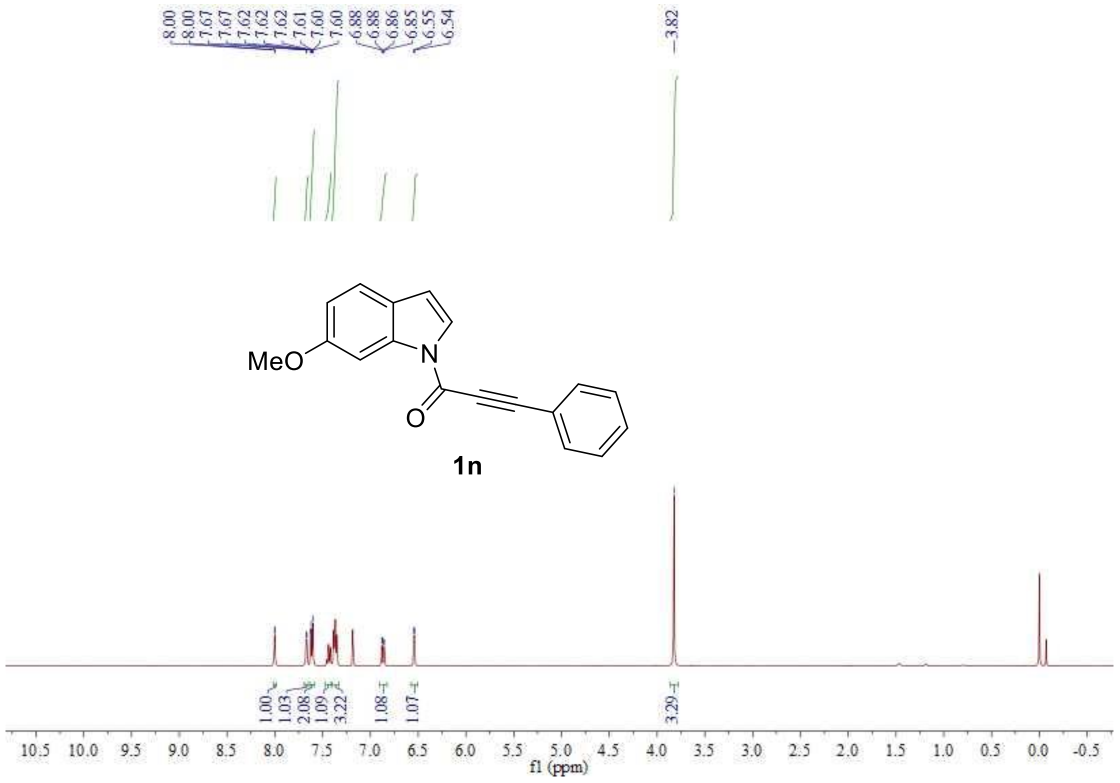

^{13}C NMR spectrum of 1n in CDCl3

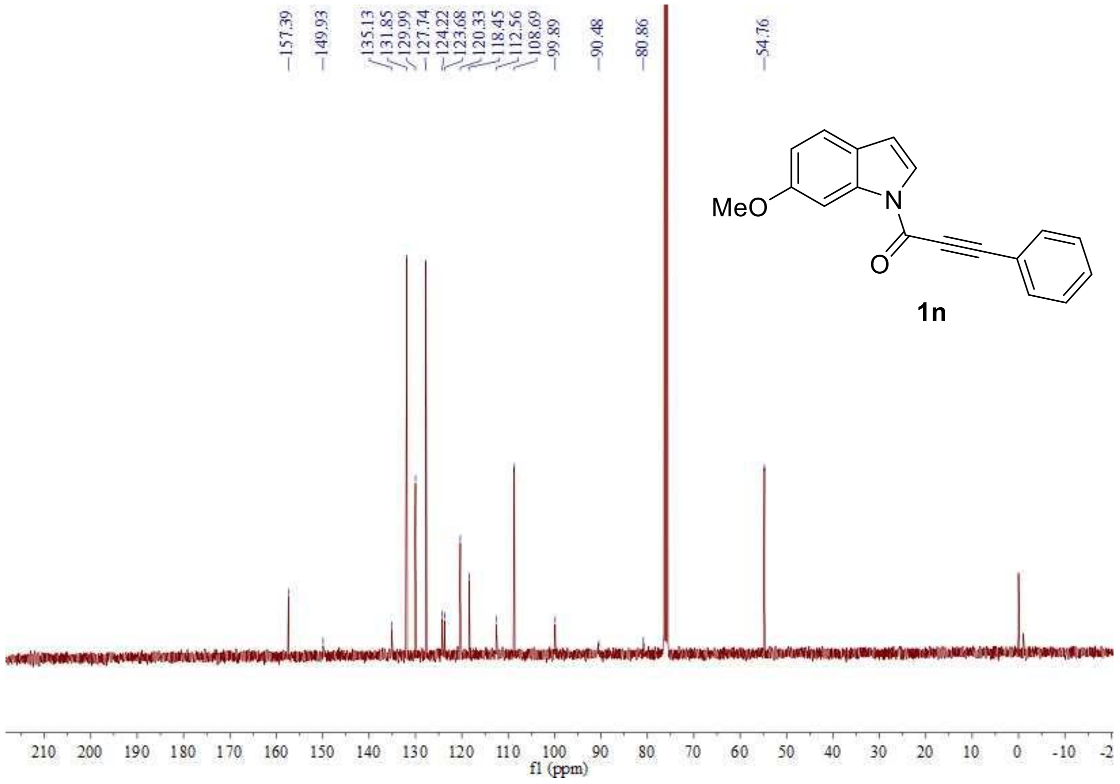

^{1}H NMR spectrum of **1o** in $CDCl_3$

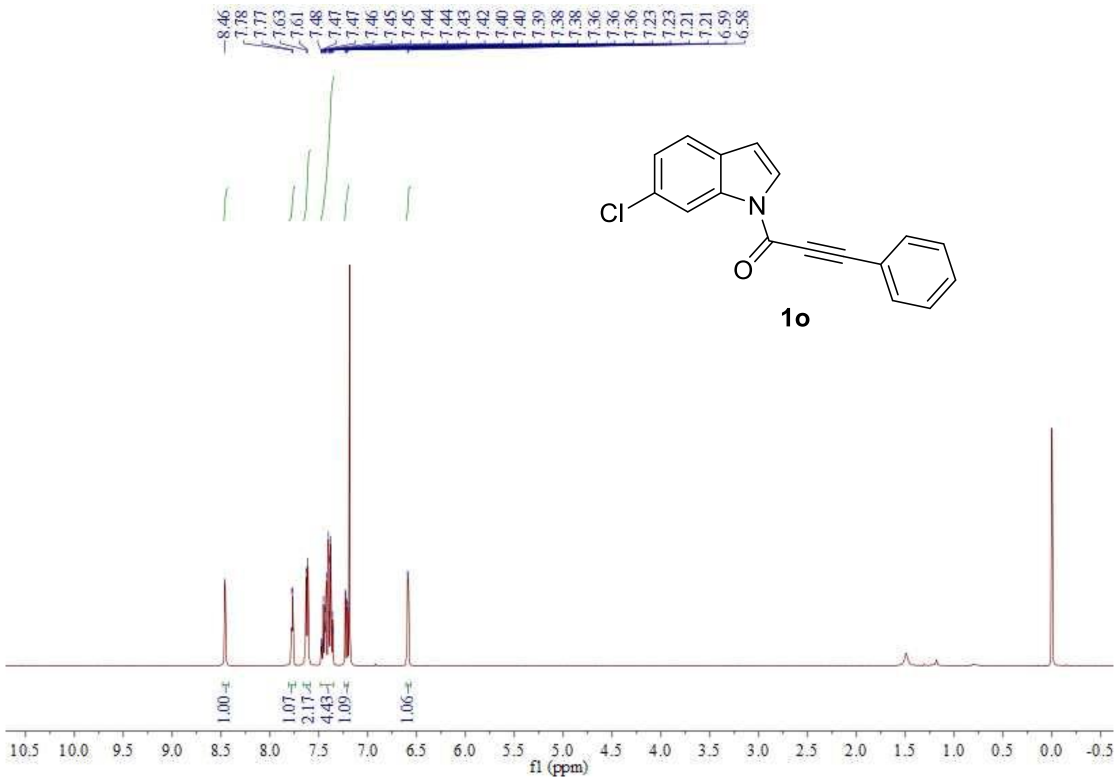

^{13}C NMR spectrum of **1o** in $CDCl_3$

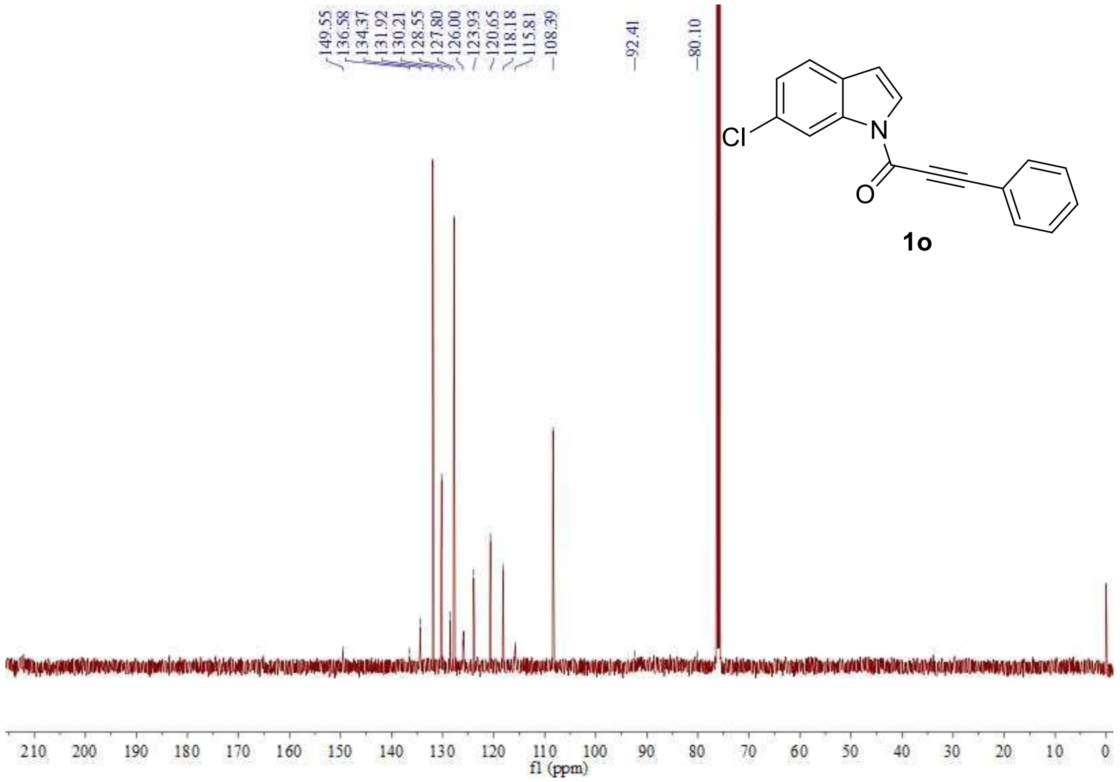

[1]**H NMR** spectrum of **1p** in $CDCl_3$

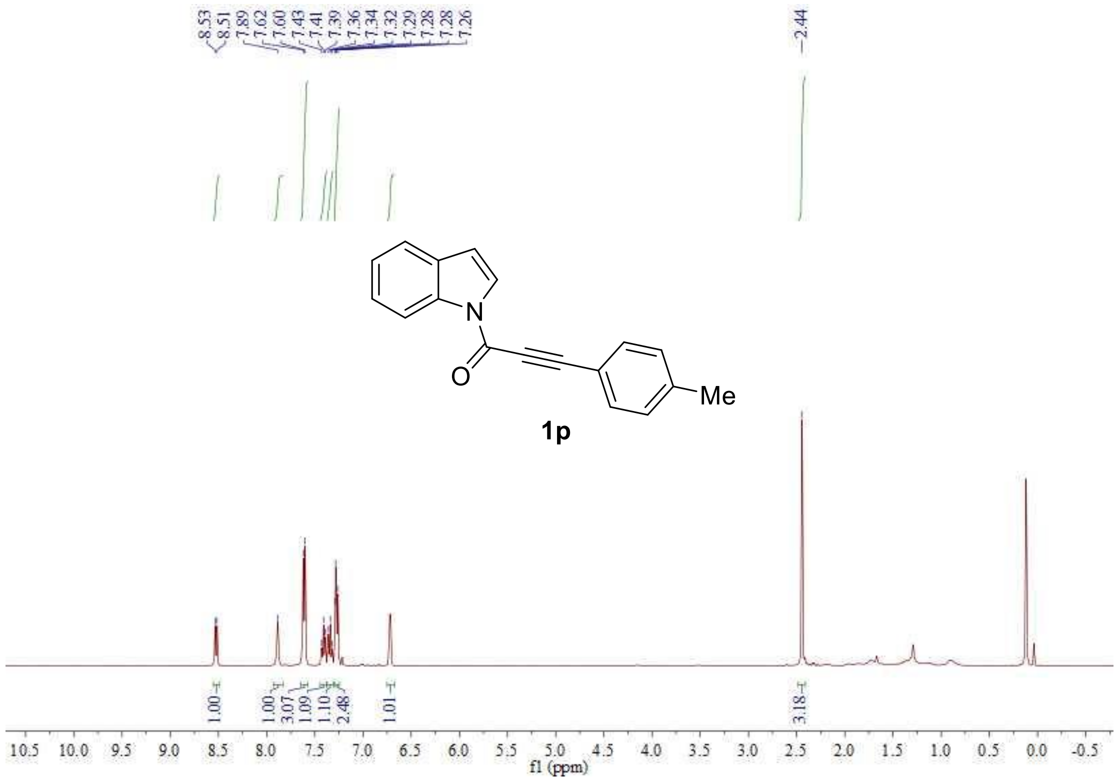

[13]**C NMR** spectrum of **1p** in $CDCl_3$

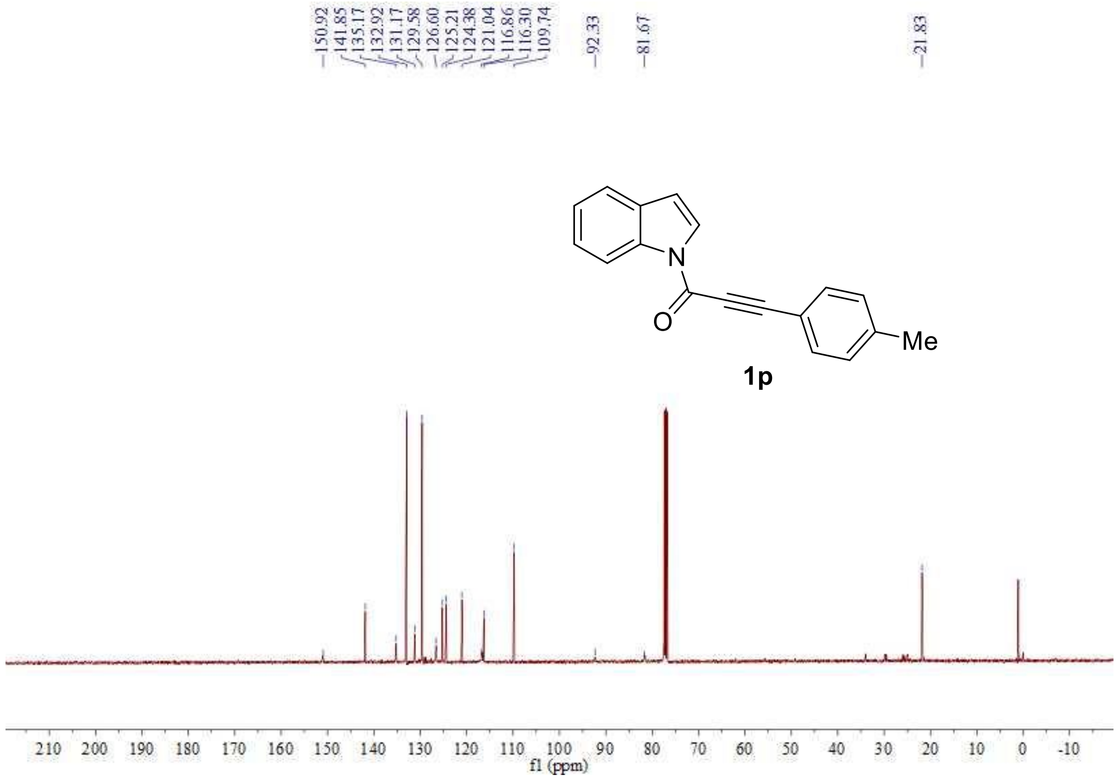

^{1}**H NMR** spectrum of **3a** in $CDCl_3$

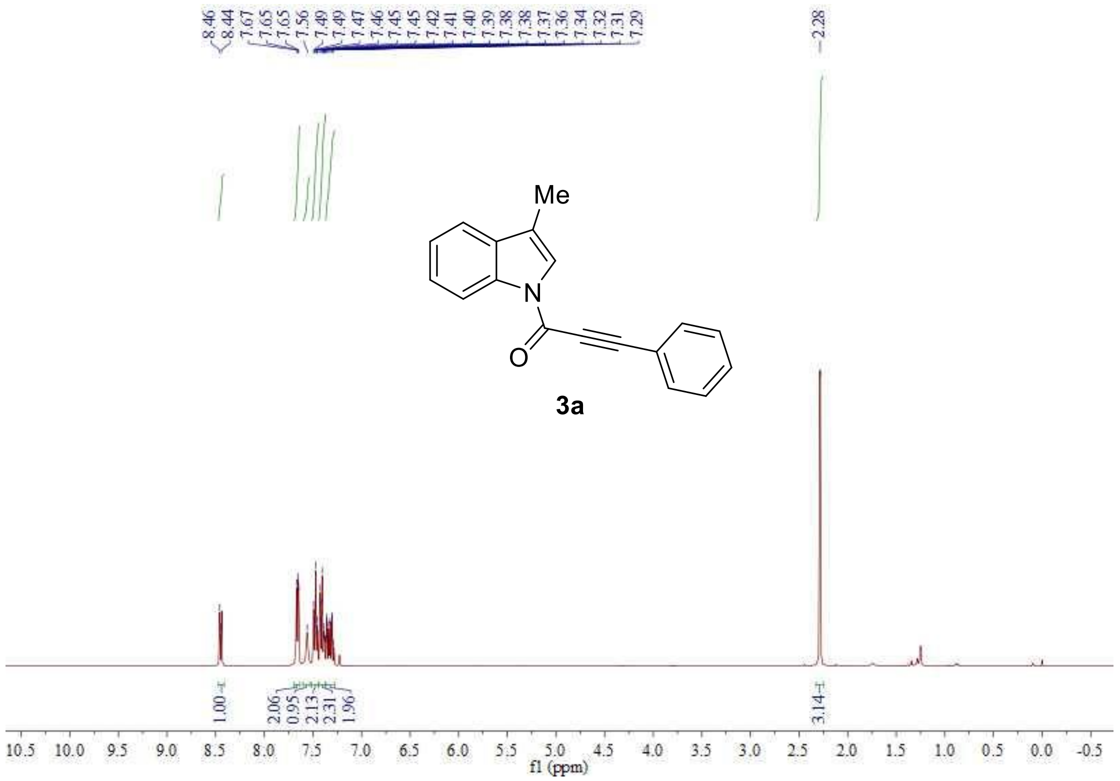

^{13}**C NMR** spectrum of **3a** in $CDCl_3$

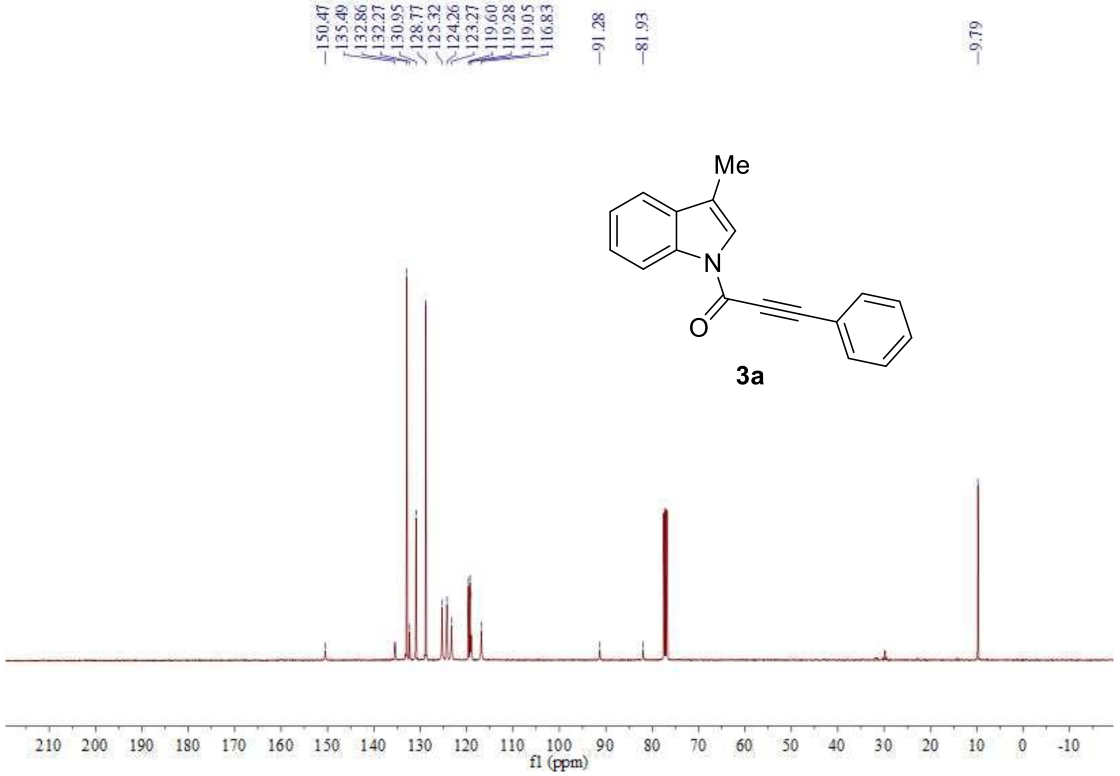

^{1}H NMR spectrum of **3b** in $CDCl_3$

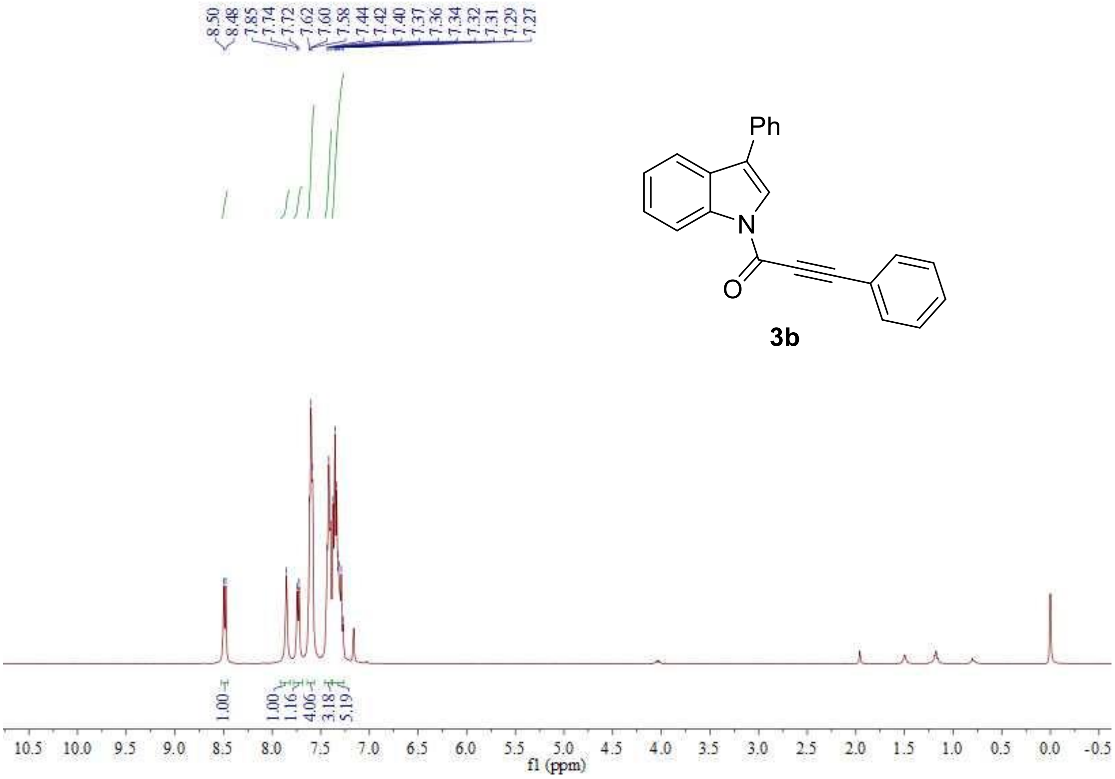

^{13}C NMR spectrum of **3b** in $CDCl_3$

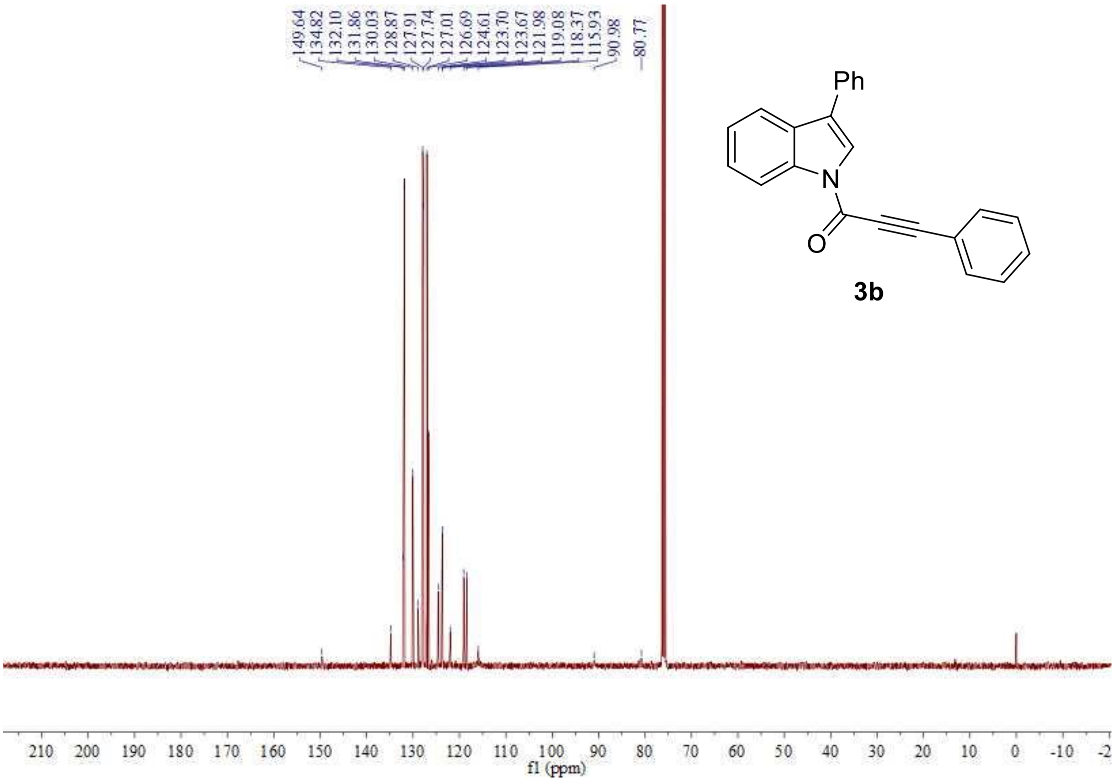

^{1}H NMR spectrum of **3c** in $CDCl_3$

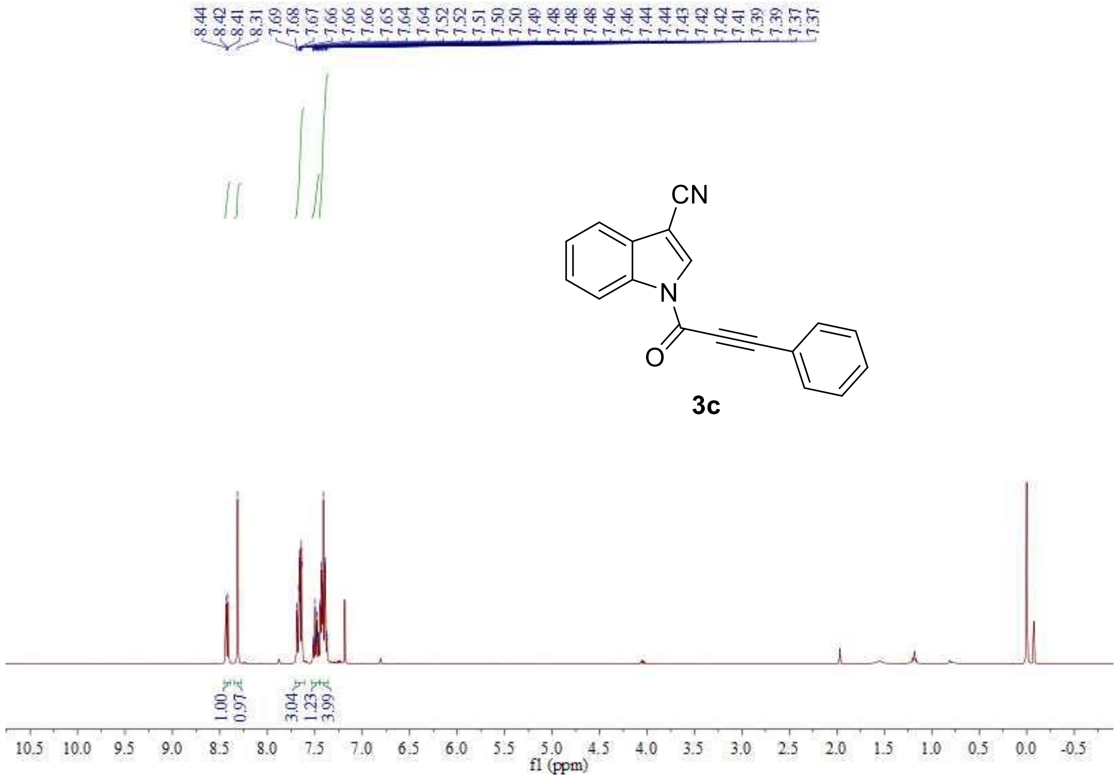

^{13}C NMR spectrum of **3c** in $CDCl_3$

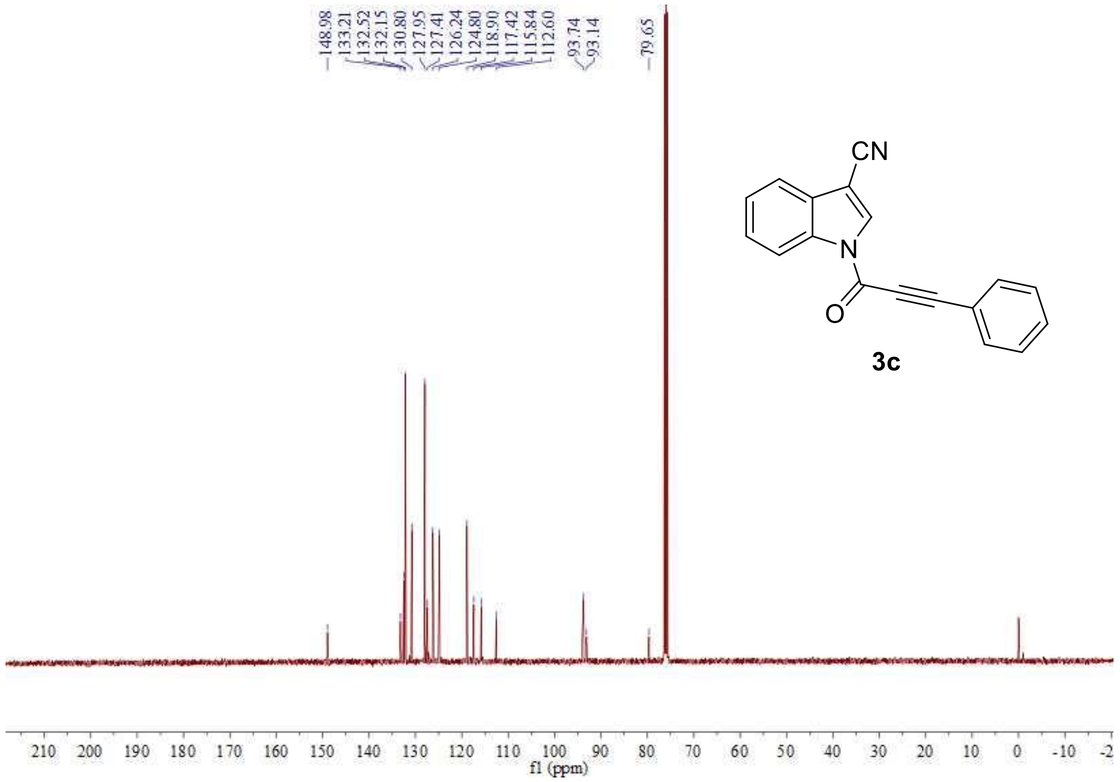

^{1}H NMR spectrum of **3d** in $CDCl_3$

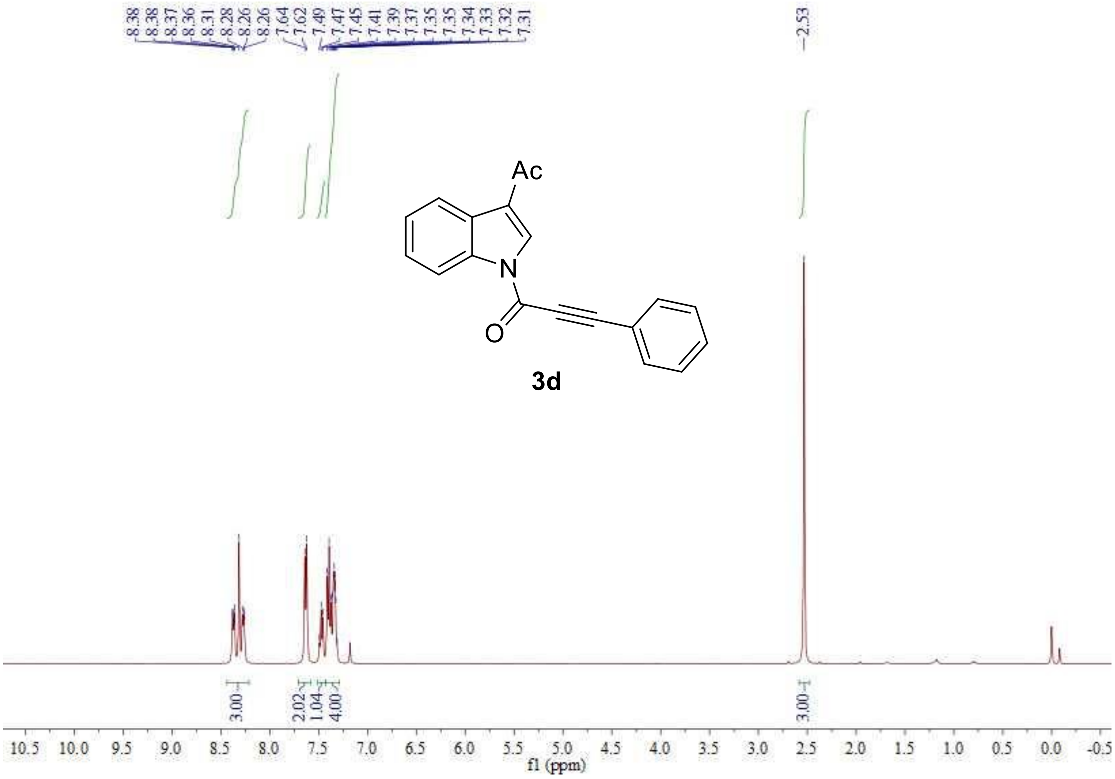

^{13}C NMR spectrum of **3d** in $CDCl_3$

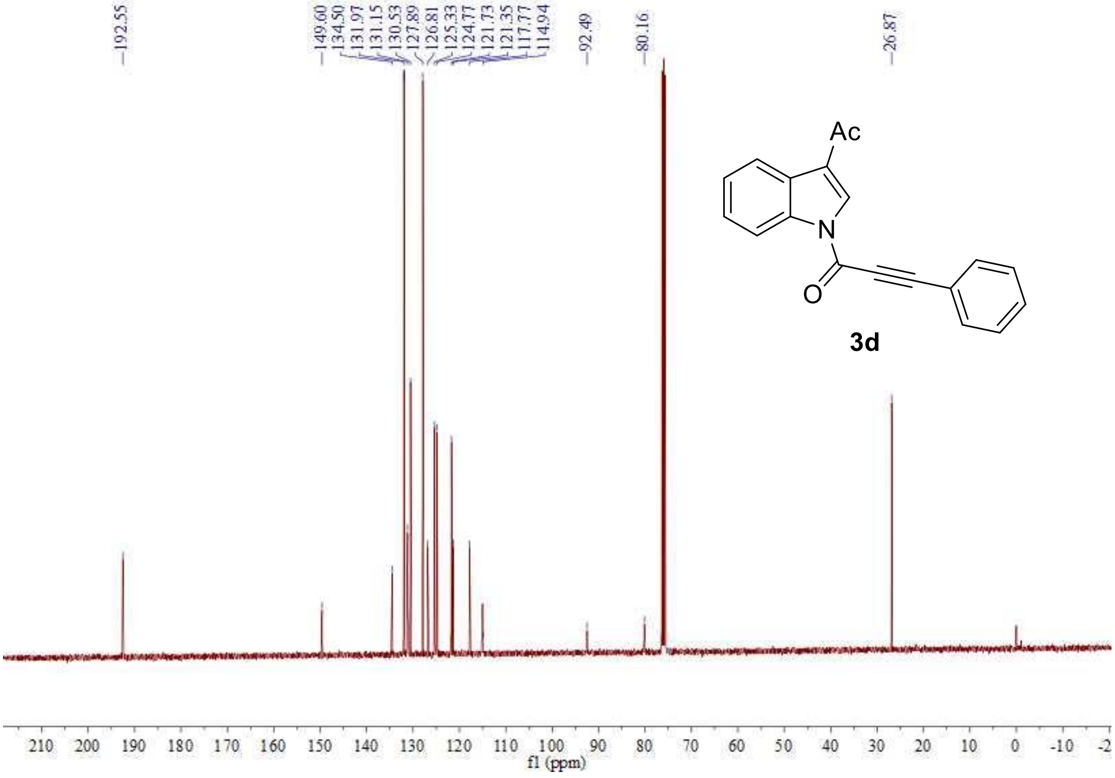

^{1}**H NMR** spectrum of **2a** in $CDCl_3$

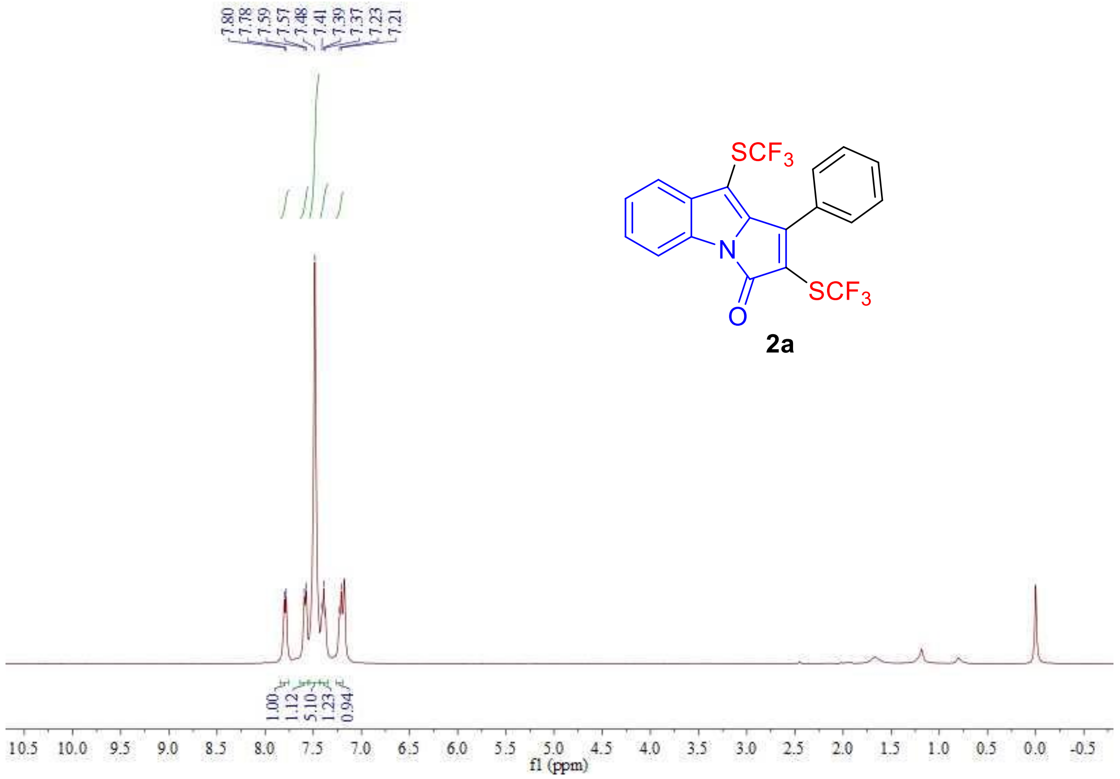

^{19}**F NMR** spectrum of **2a** in $CDCl_3$

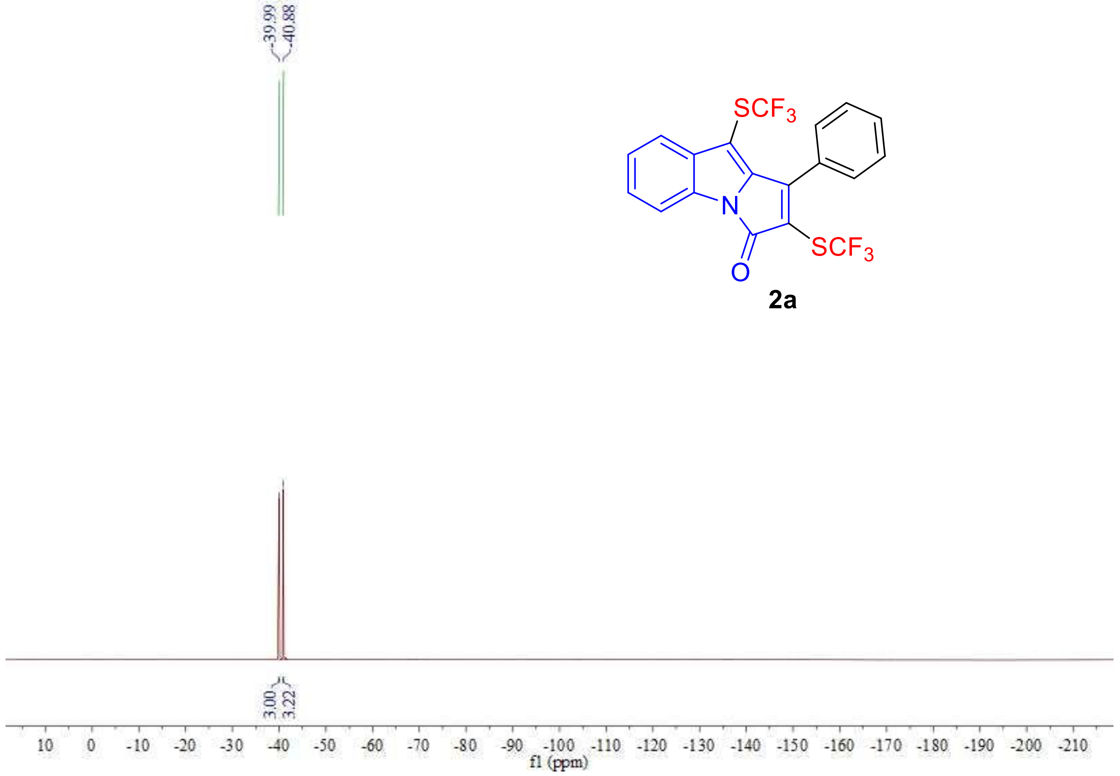

^{13}C **NMR** spectrum of **2a** in CDCl3

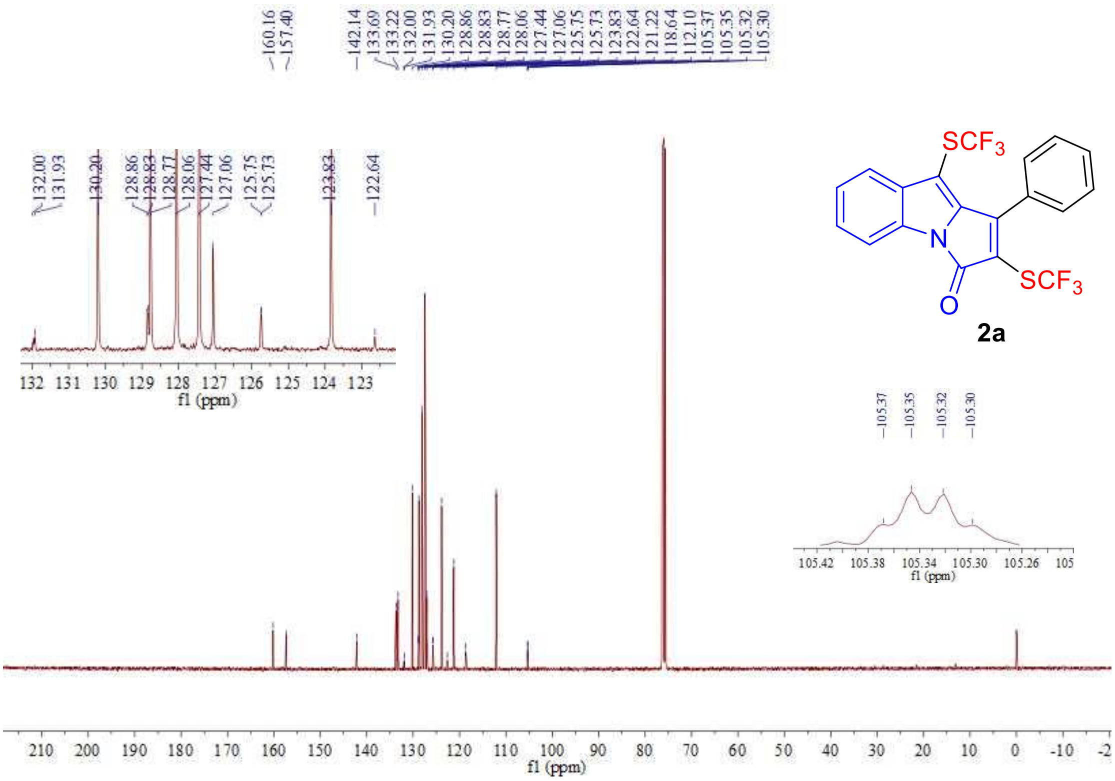

^{1}H **NMR** spectrum of **2b** in CDCl3

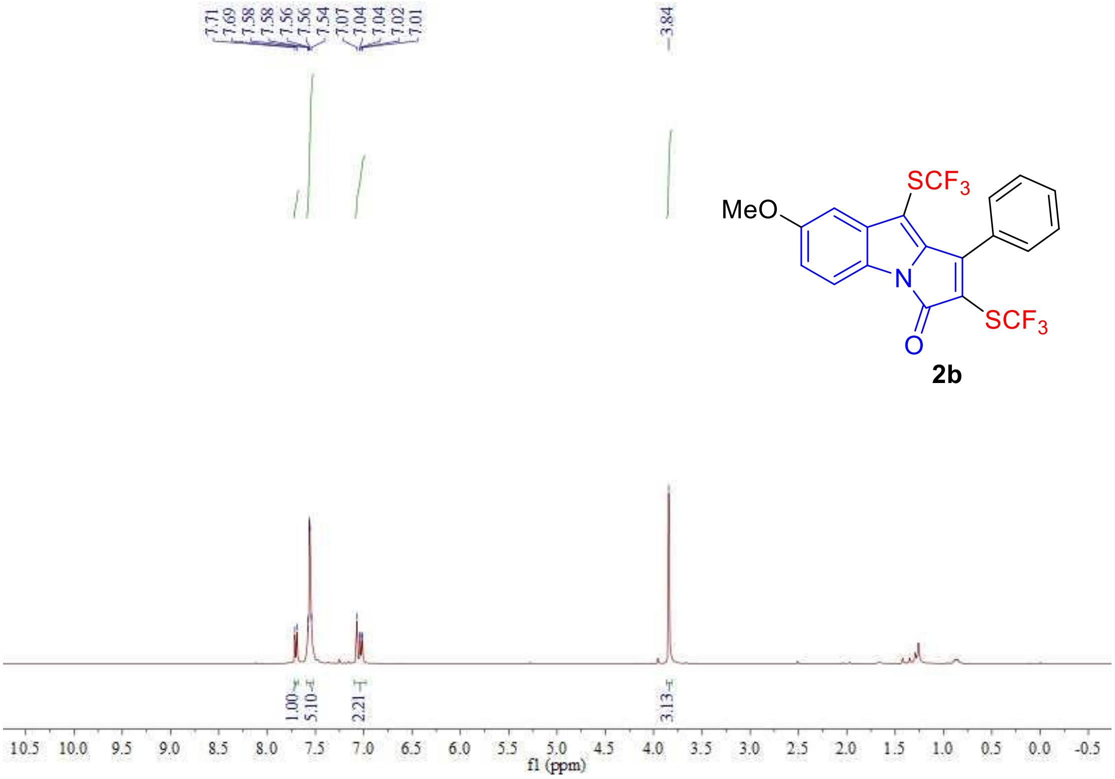

^{19}F **NMR** spectrum of **2b** in $CDCl_3$

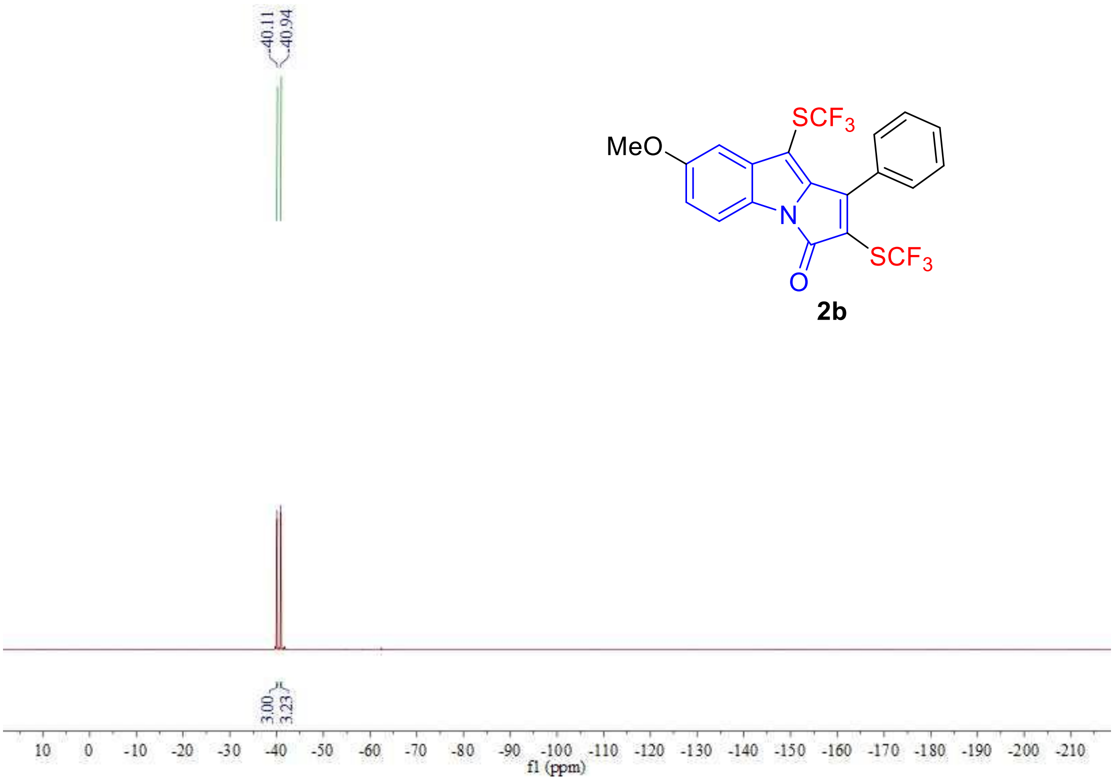

^{13}C **NMR** spectrum of **2b** in $CDCl_3$

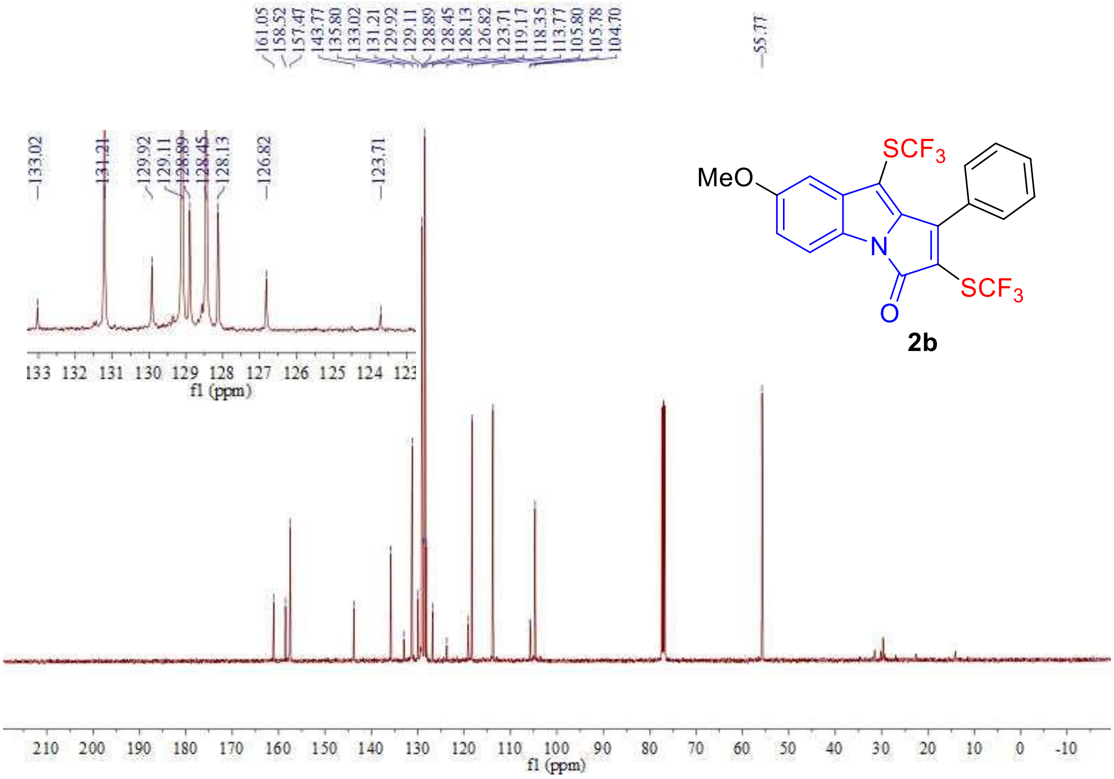

^{1}H NMR spectrum of **2c** in $CDCl_3$

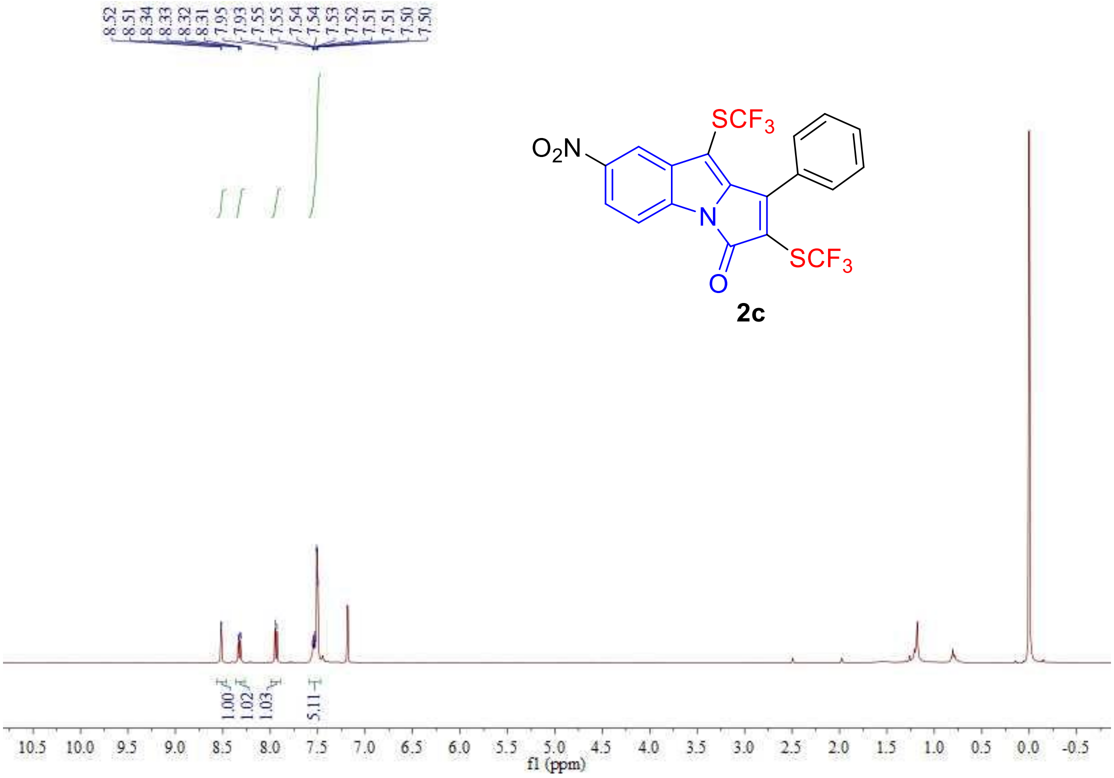

^{19}F NMR spectrum of **2c** in $CDCl_3$

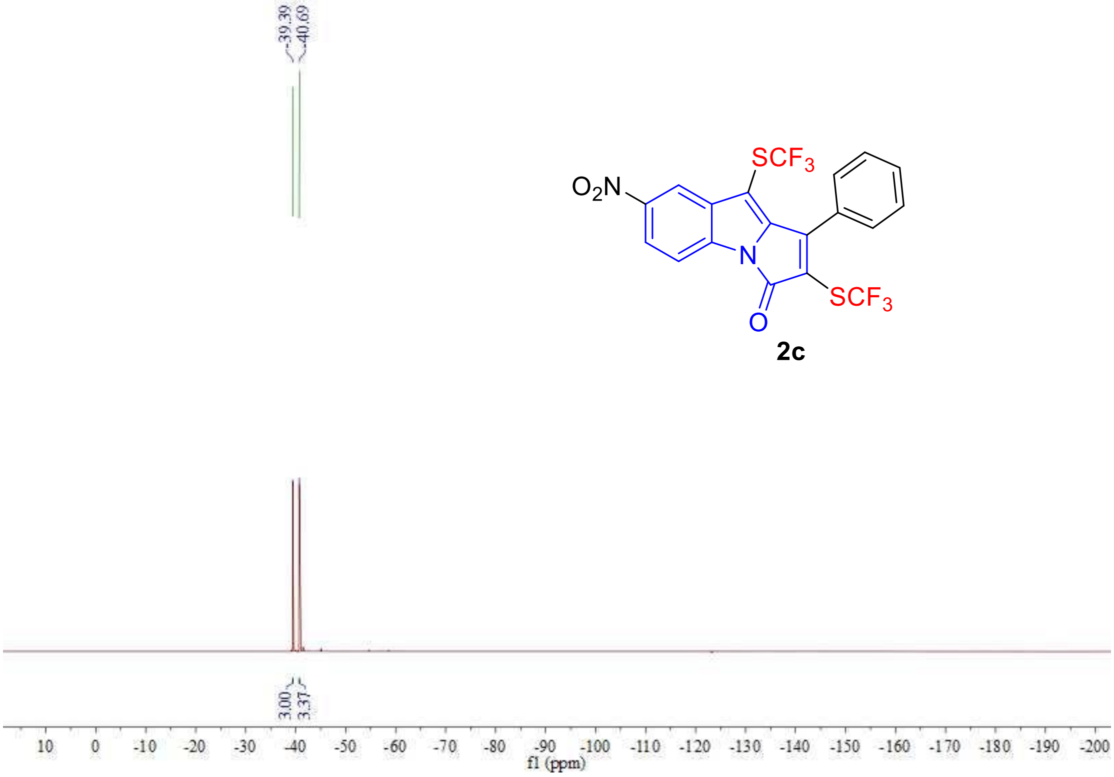

^{13}C NMR spectrum of **2c** in $CDCl_3$

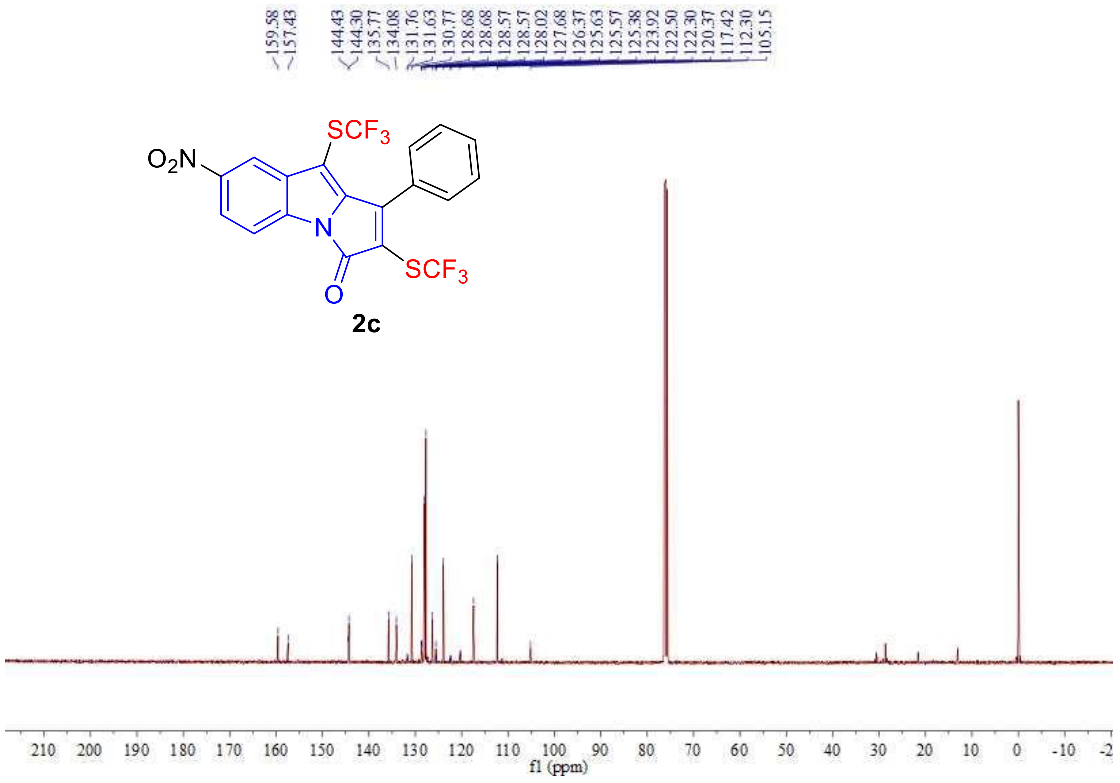

^{1}H NMR spectrum of **2d** in $CDCl_3$

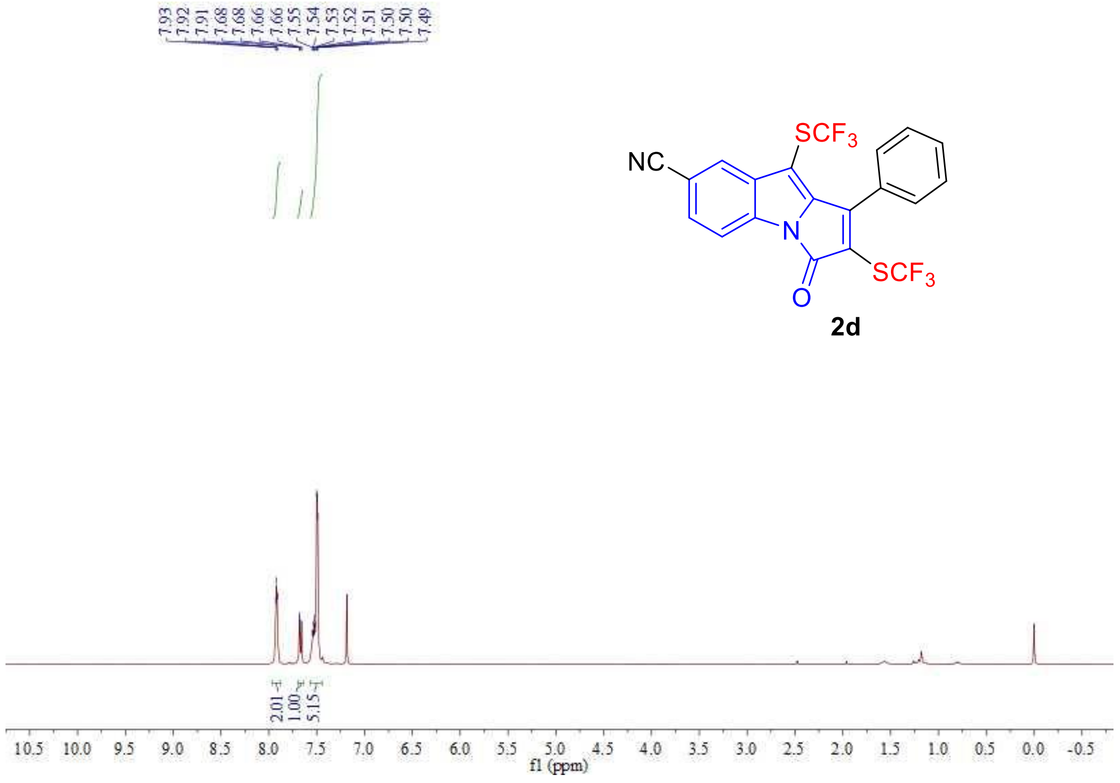

^{19}F NMR spectrum of **2d** in CDCl3

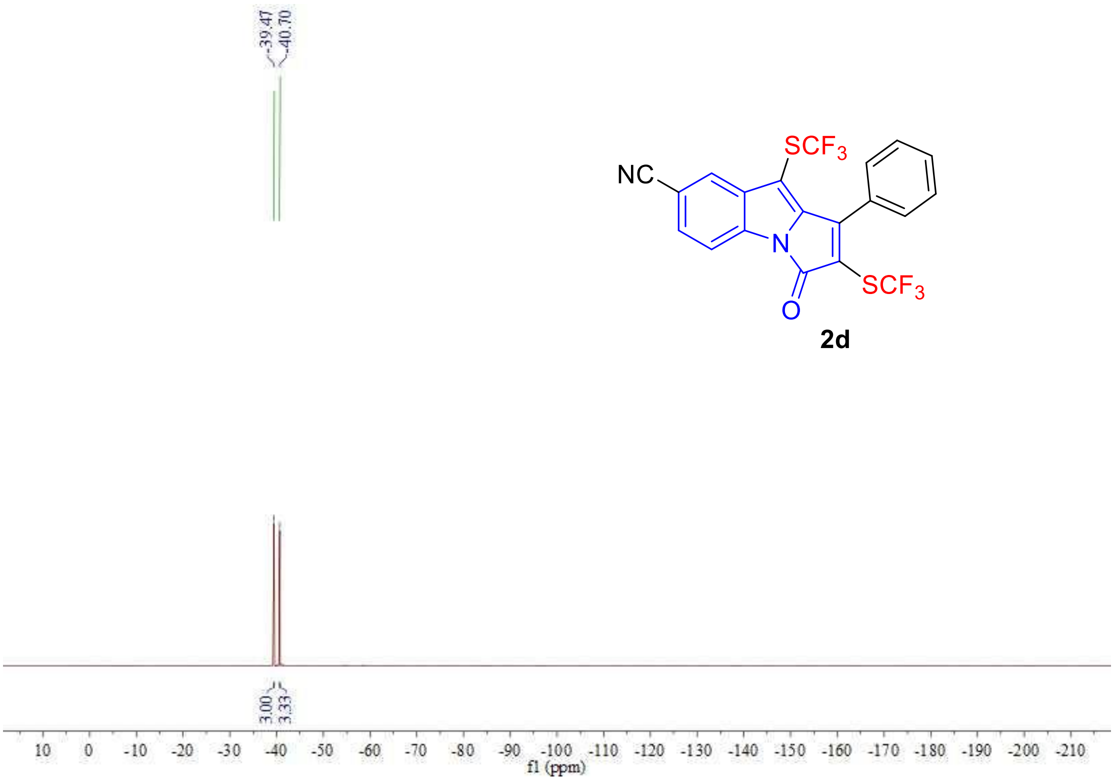

^{13}C NMR spectrum of **2d** in CDCl3

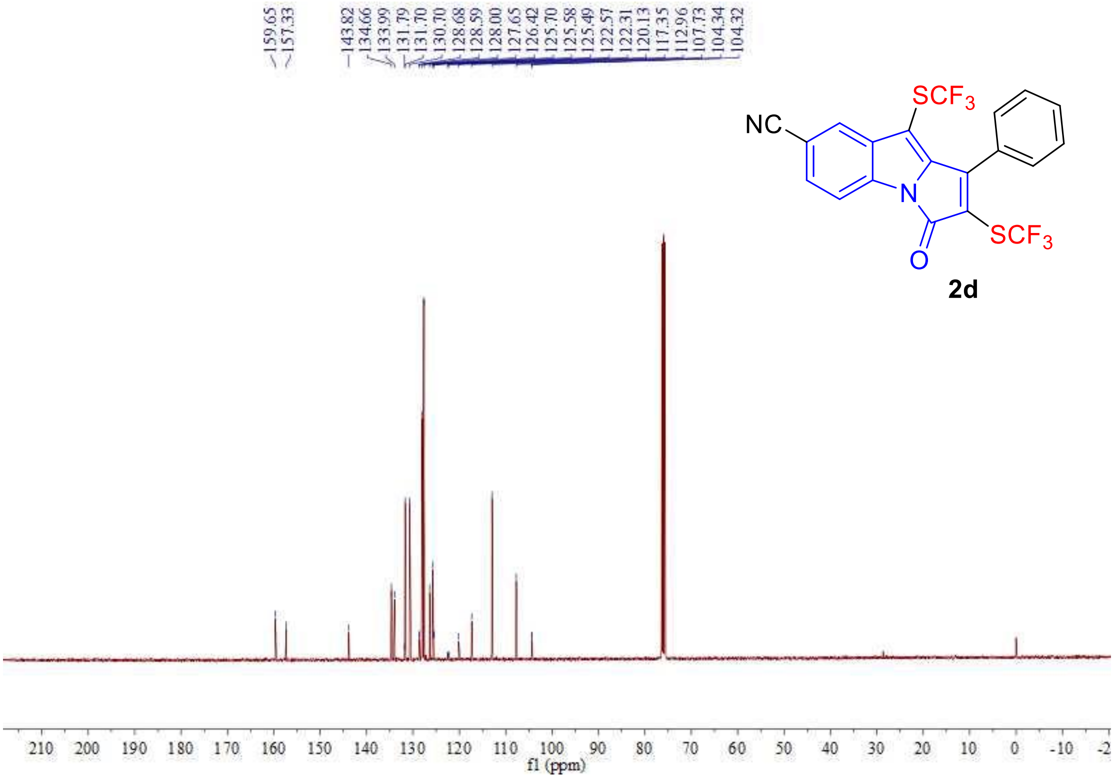

^{1}H NMR spectrum of **2e** in CDCl3

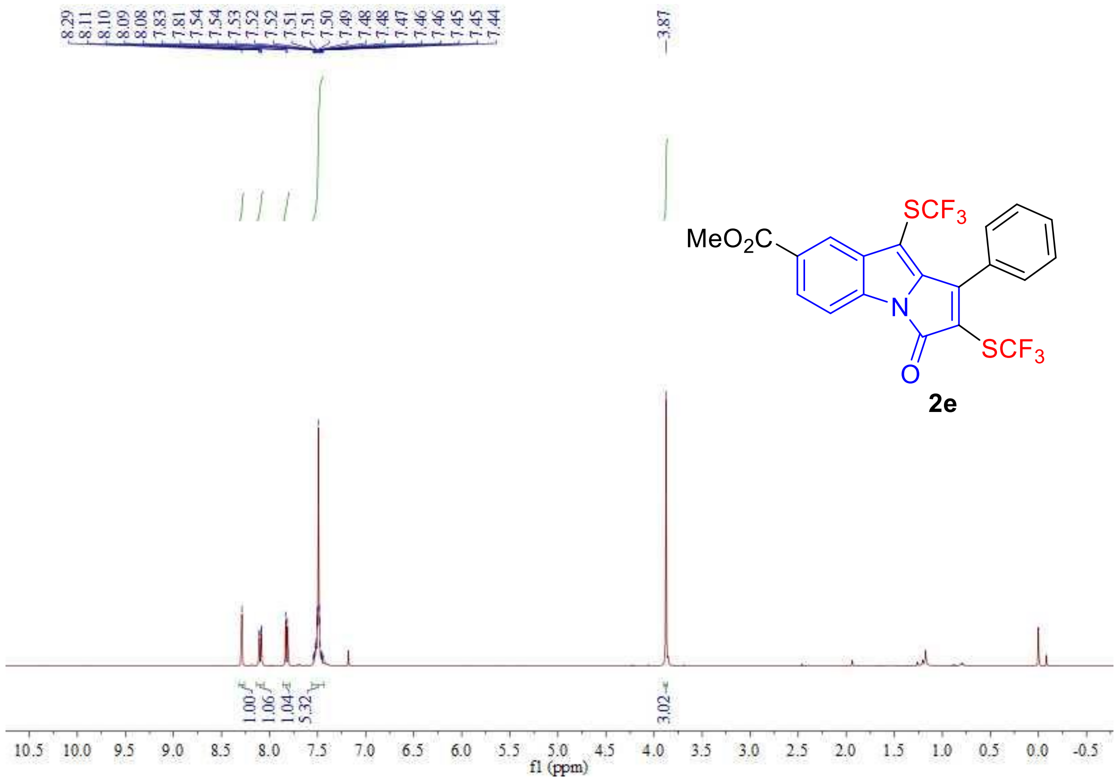

^{19}F NMR spectrum of **2e** in CDCl3

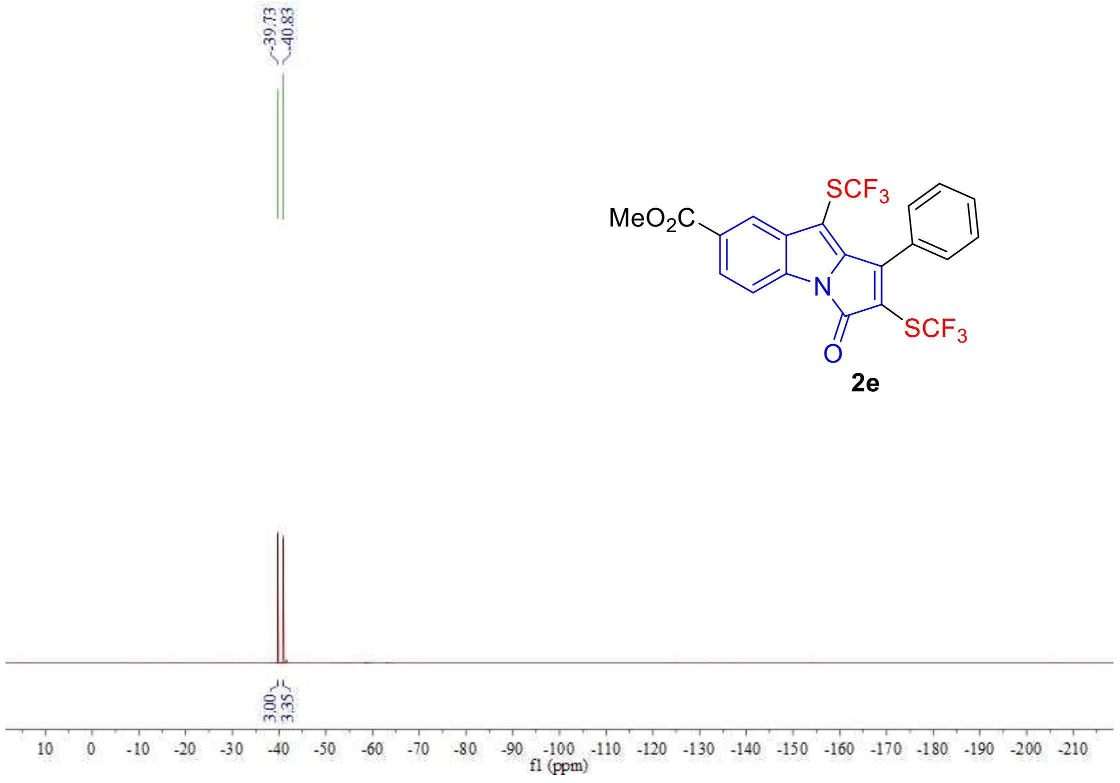

^{13}C NMR spectrum of **2e** in $CDCl_3$

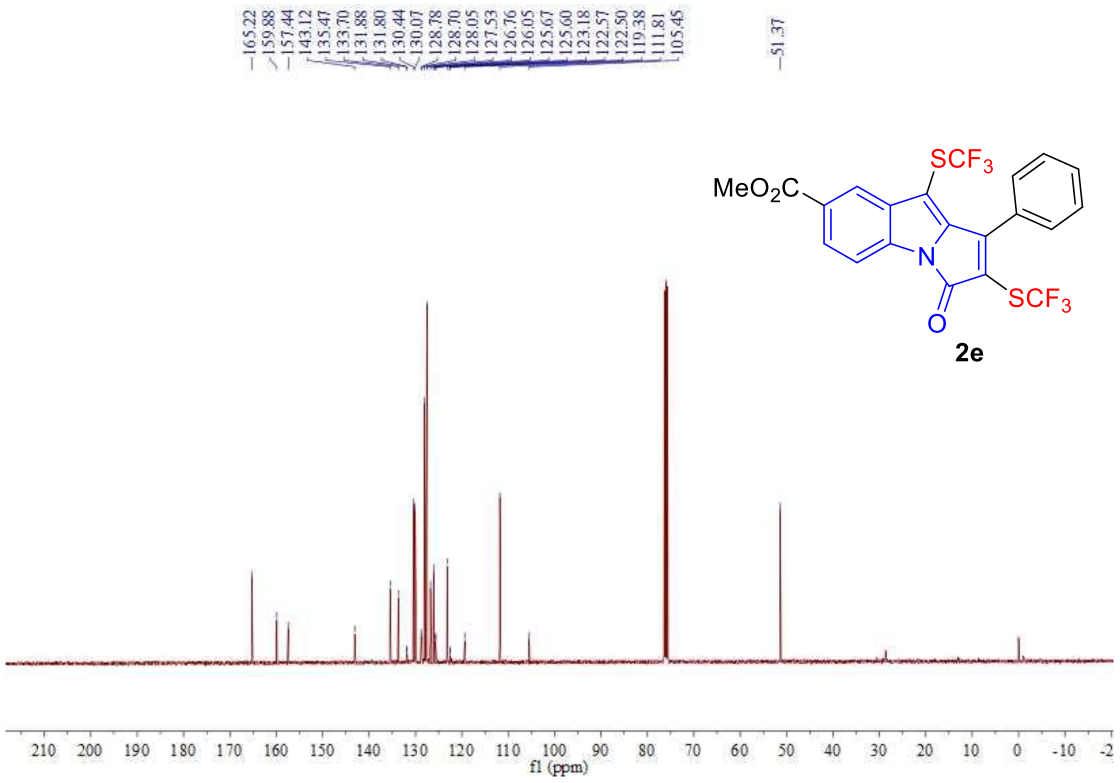

^{1}H NMR spectrum of **2f** in $CDCl_3$

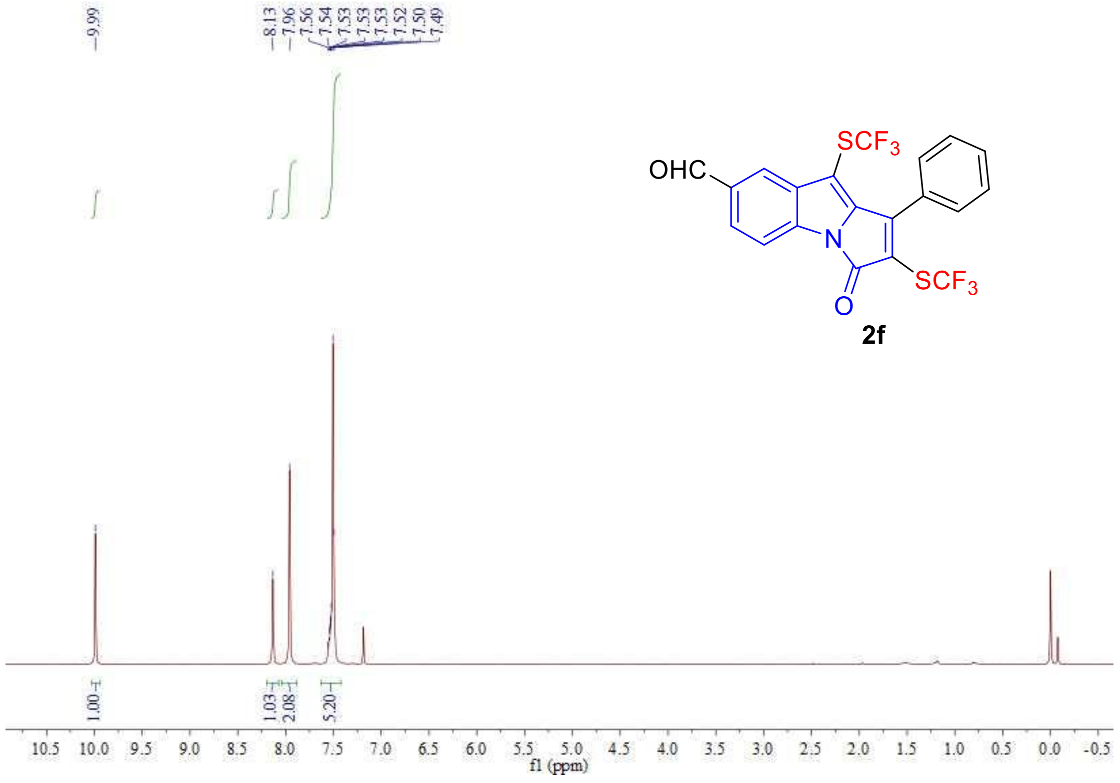

^{19}F NMR spectrum of **2f** in $CDCl_3$

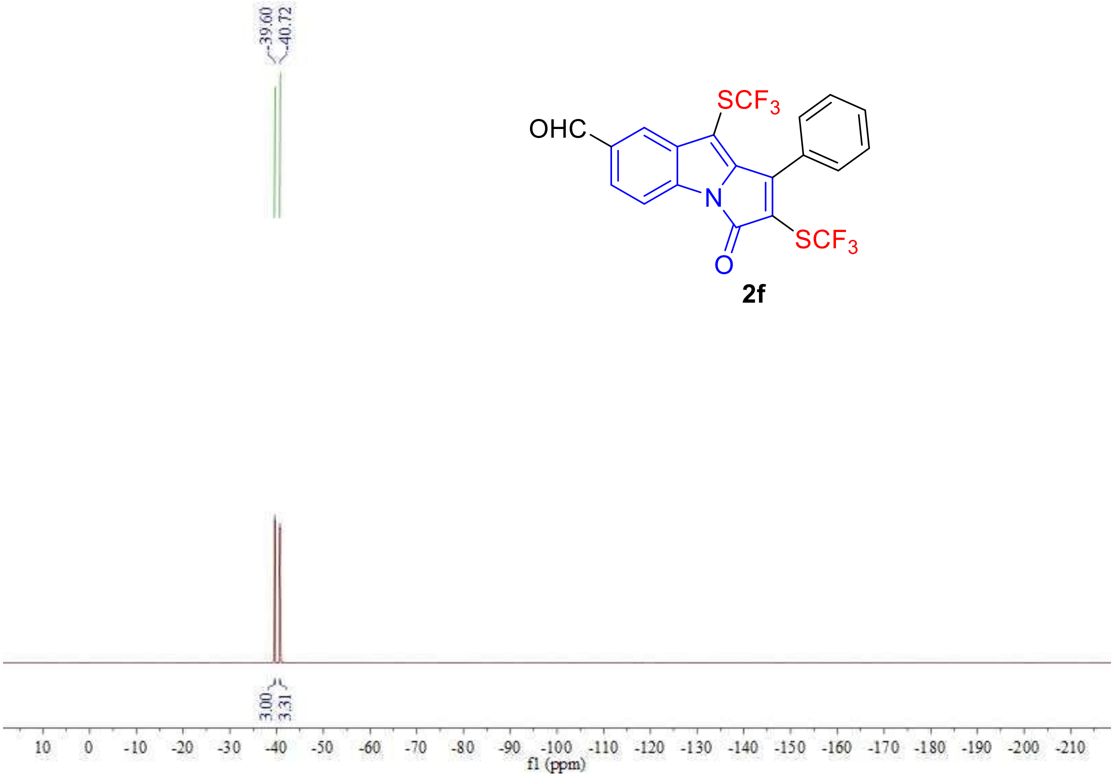

^{13}C NMR spectrum of **2f** in $CDCl_3$

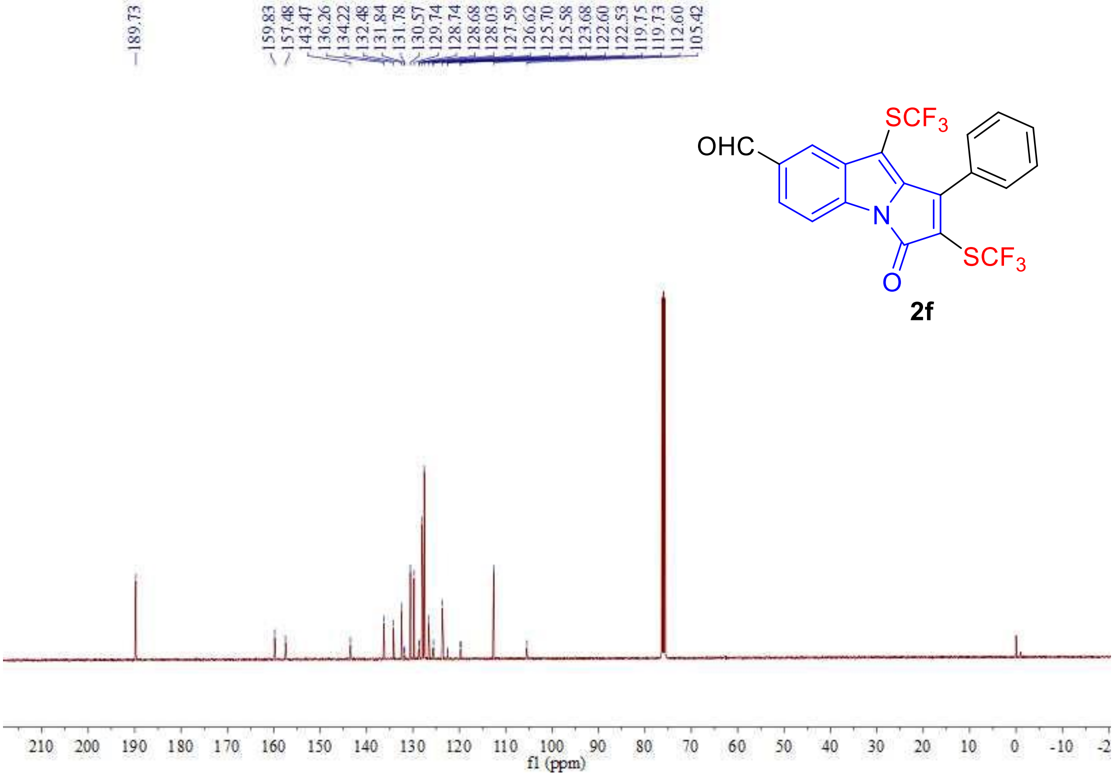

^{1}H NMR spectrum of **2g** in CDCl3

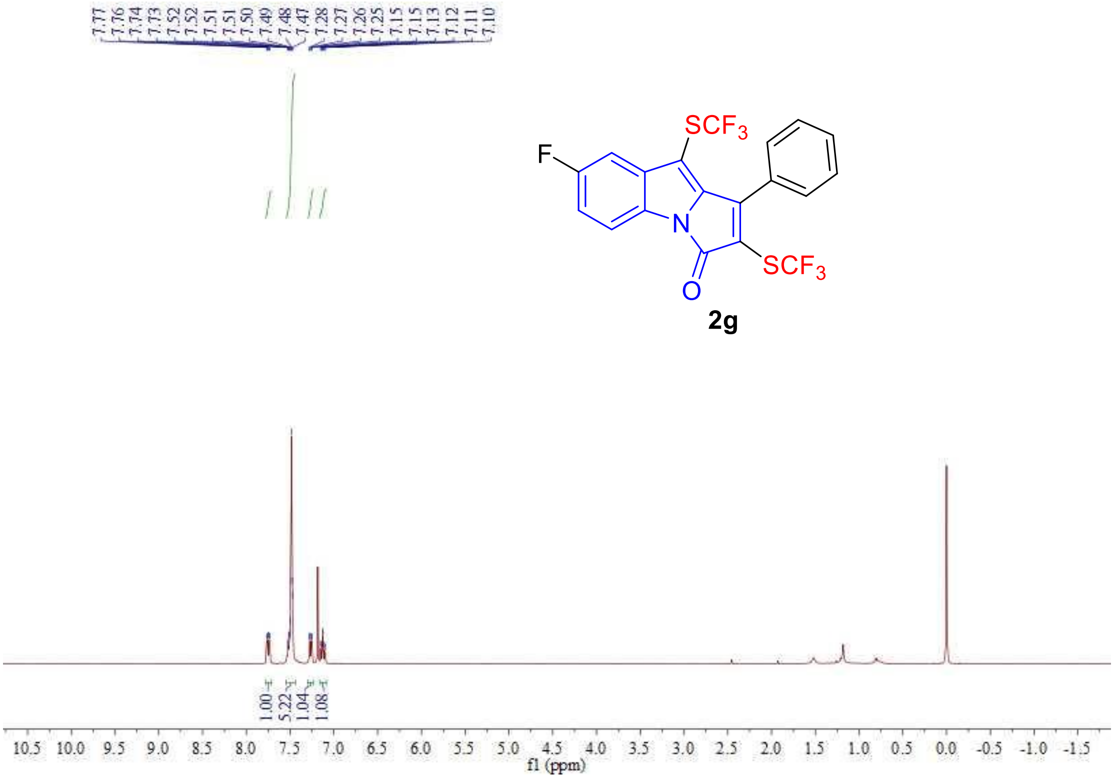

^{19}F NMR spectrum of **2g** in CDCl3

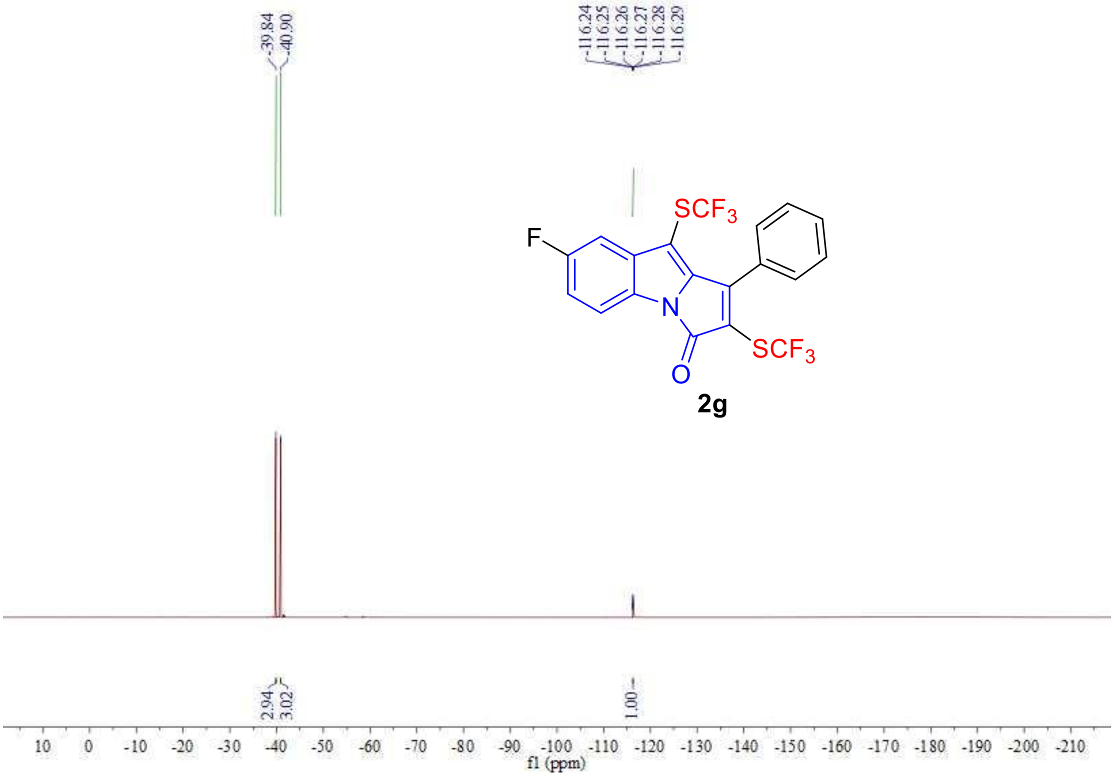

^{13}C NMR spectrum of **2g** in $CDCl_3$

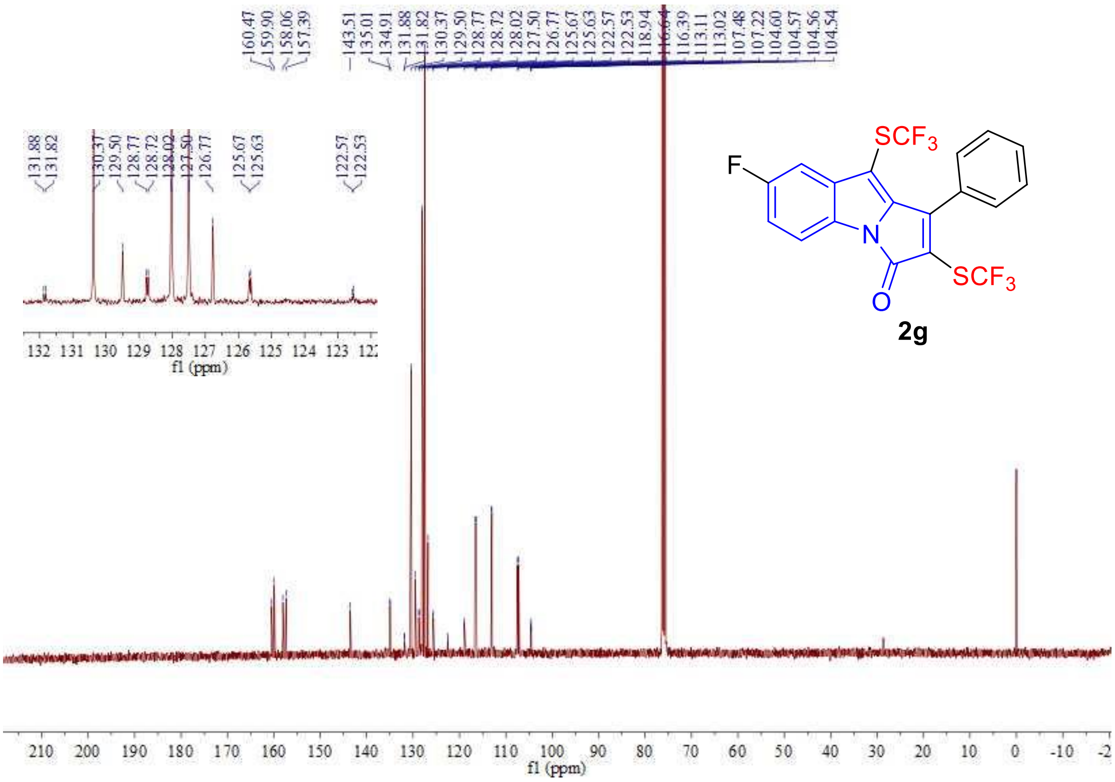

^{1}H NMR spectrum of **2h** in $CDCl_3$

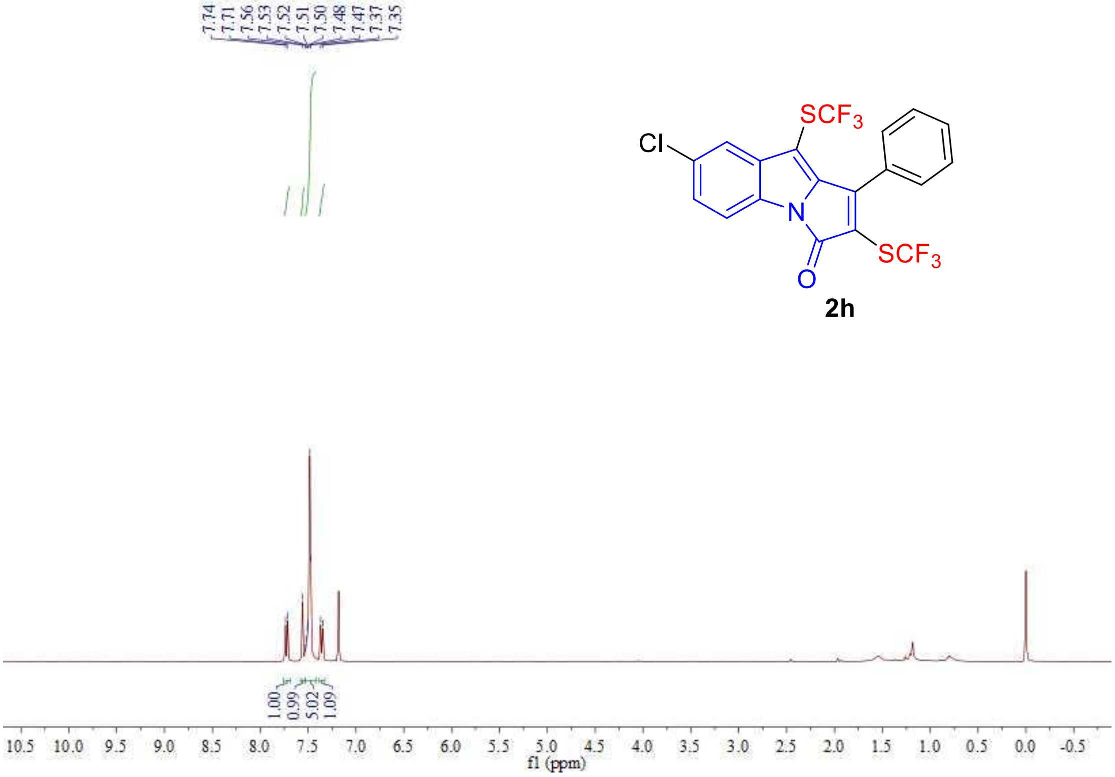

^{19}F NMR spectrum of **2h** in $CDCl_3$

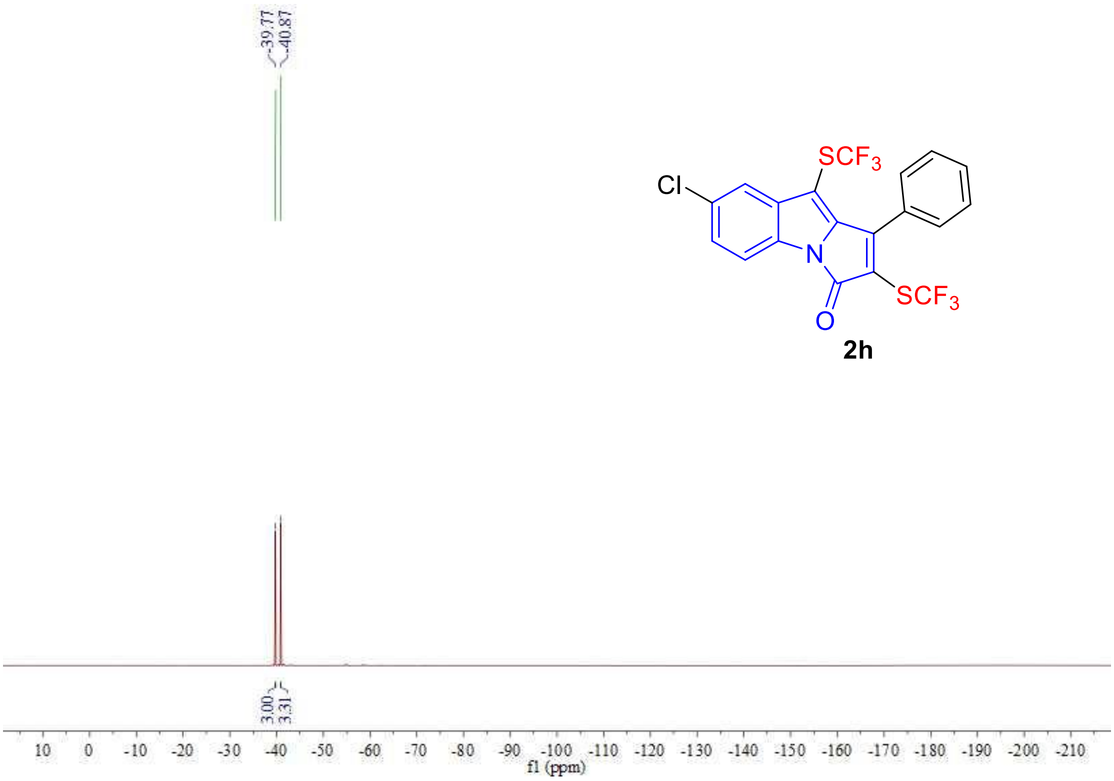

^{13}C NMR spectrum of **2h** in $CDCl_3$

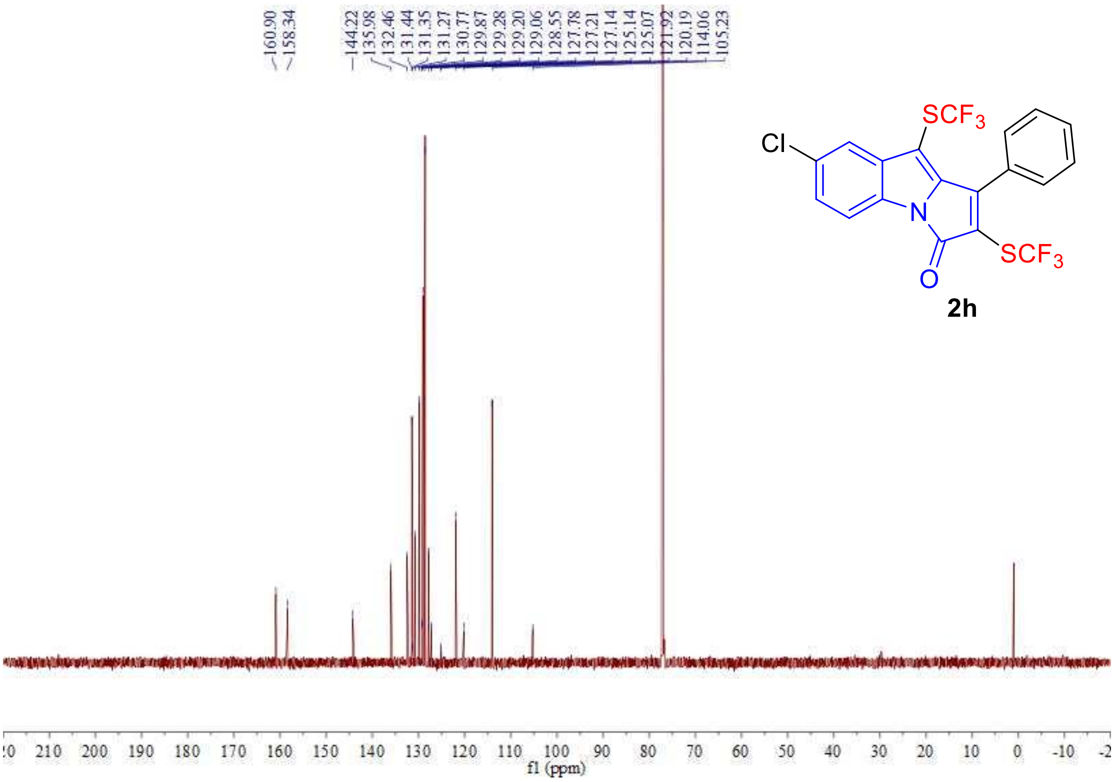

[1]**H NMR** spectrum of **2i** in $CDCl_3$

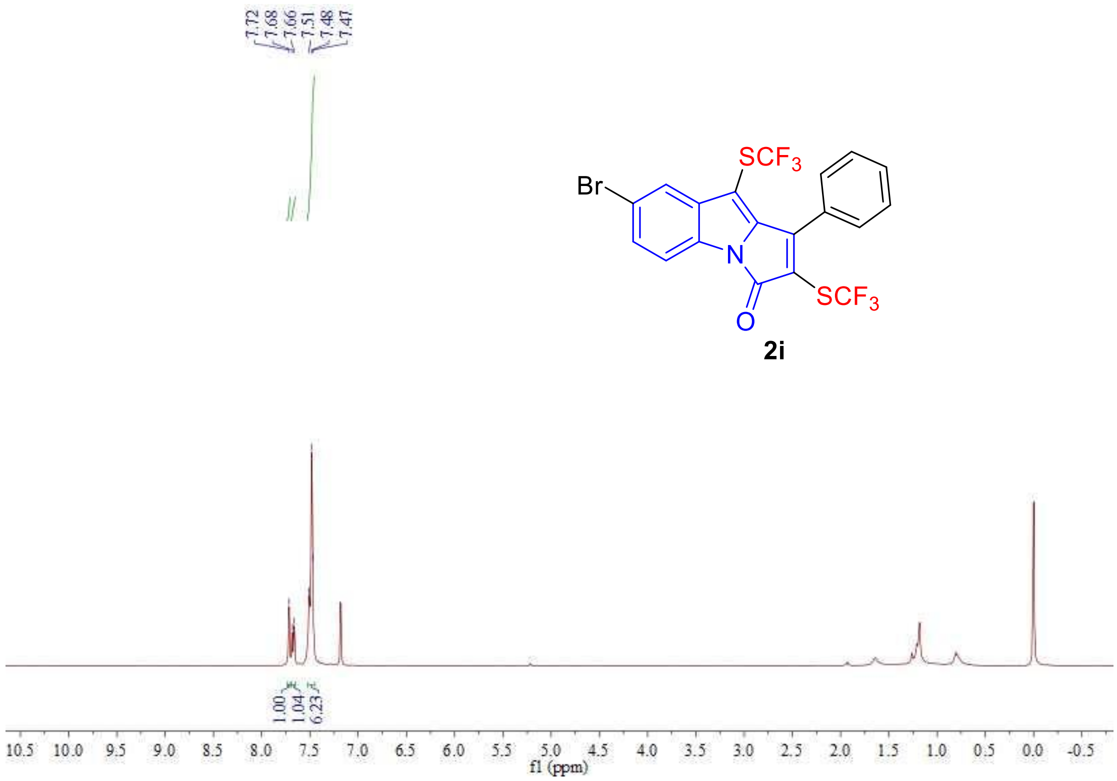

[19]**F NMR** spectrum of **2i** in $CDCl_3$

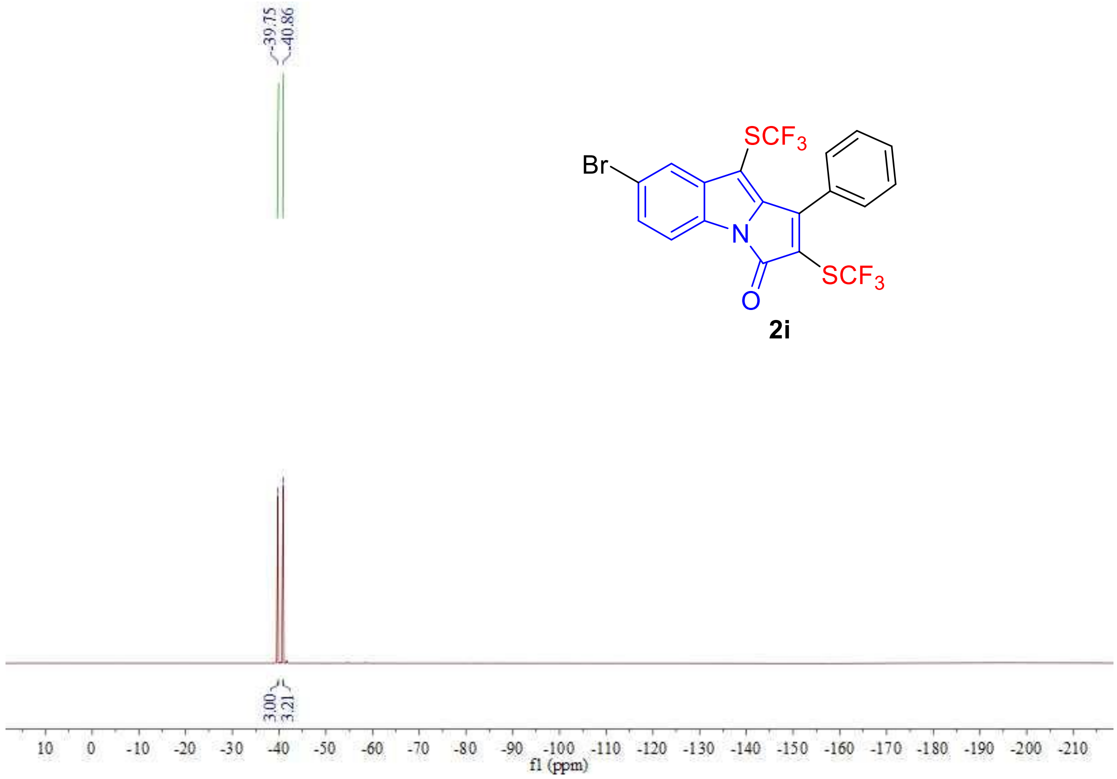

^{13}C NMR spectrum of **2i** in CDCl3

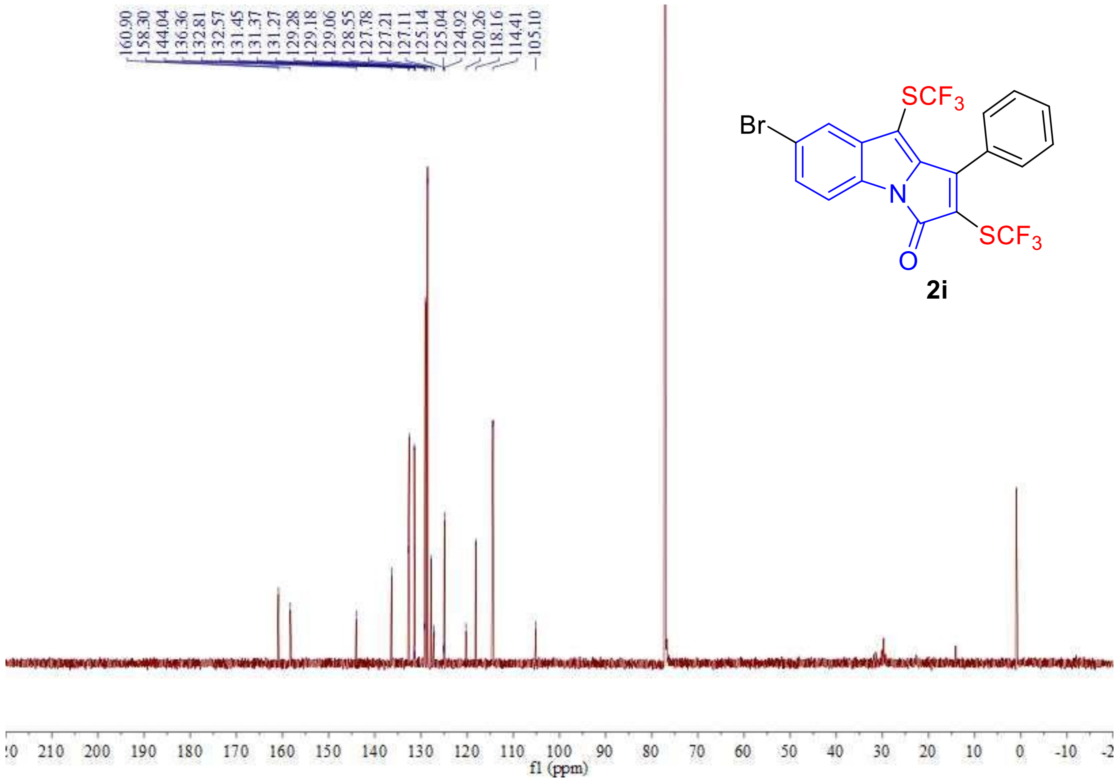

^{1}H NMR spectrum of **2j** in CDCl3

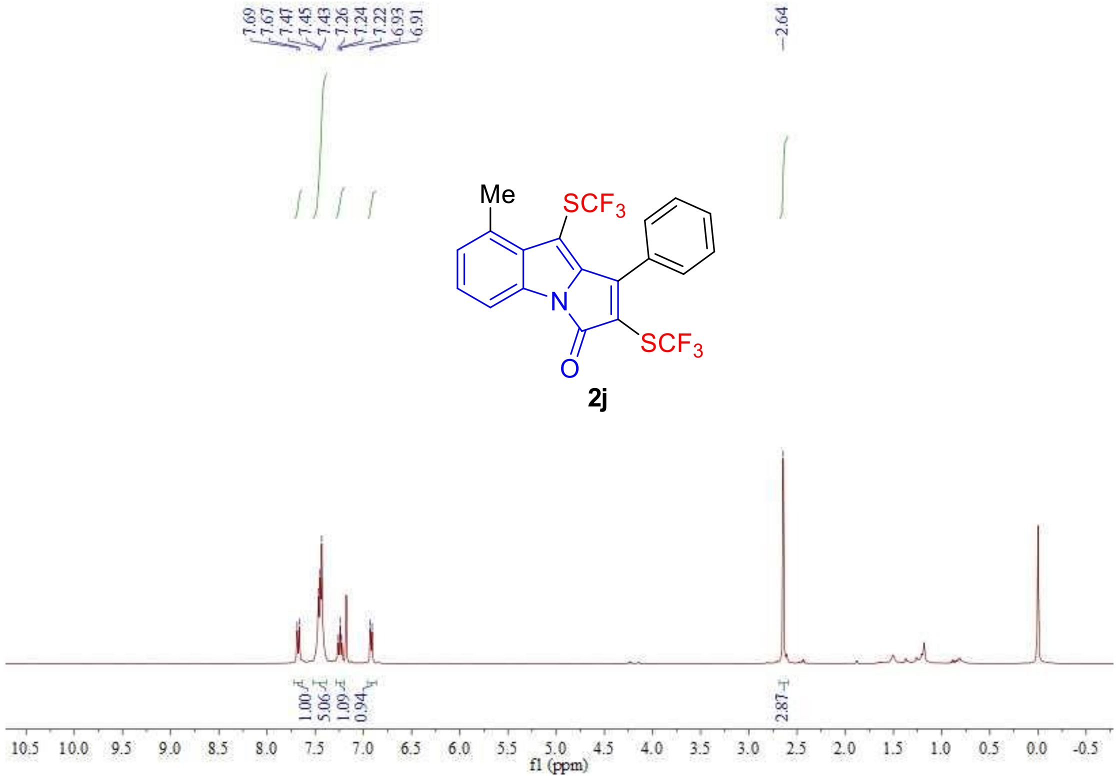

^{19}**F NMR** spectrum of **2j** in $CDCl_3$

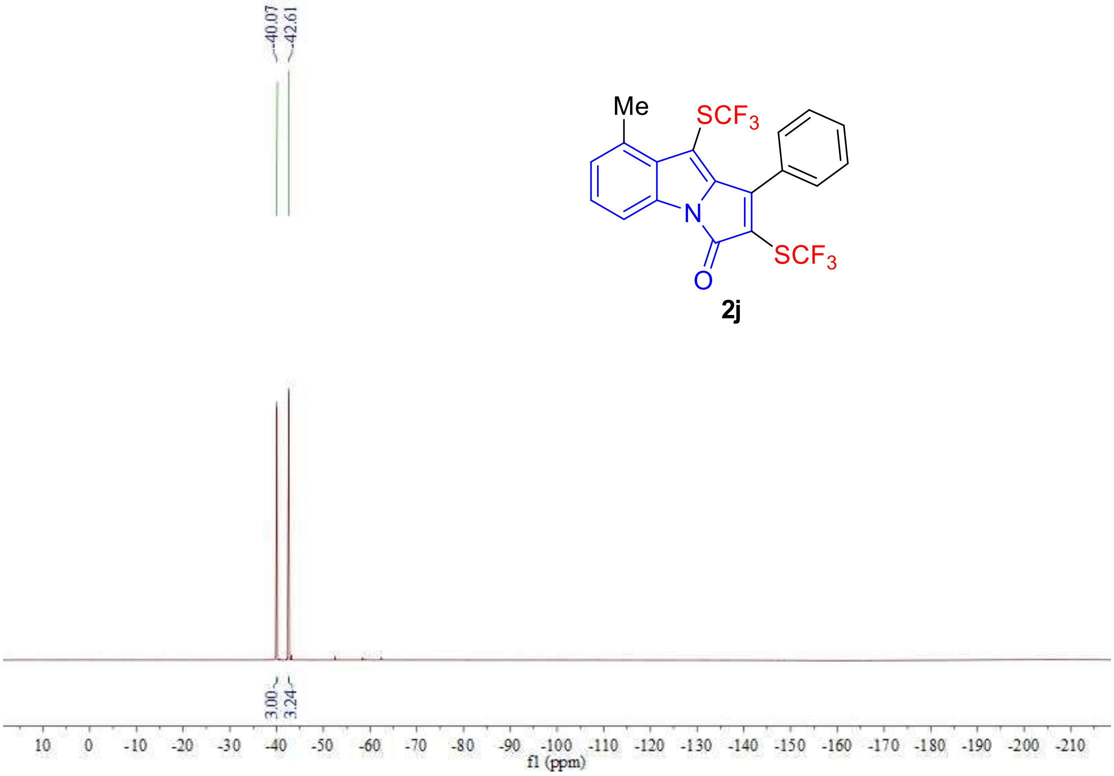

^{13}**C NMR** spectrum of **2j** in $CDCl_3$

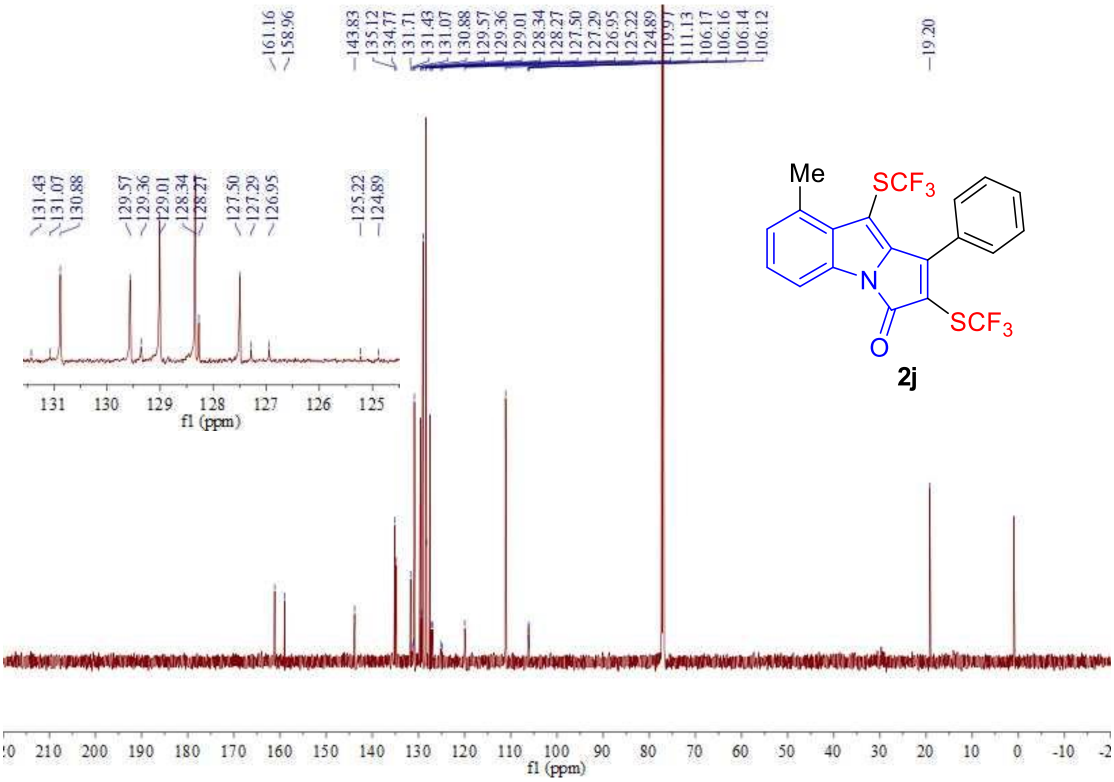

^{1}**H NMR** spectrum of **2k** in $CDCl_3$

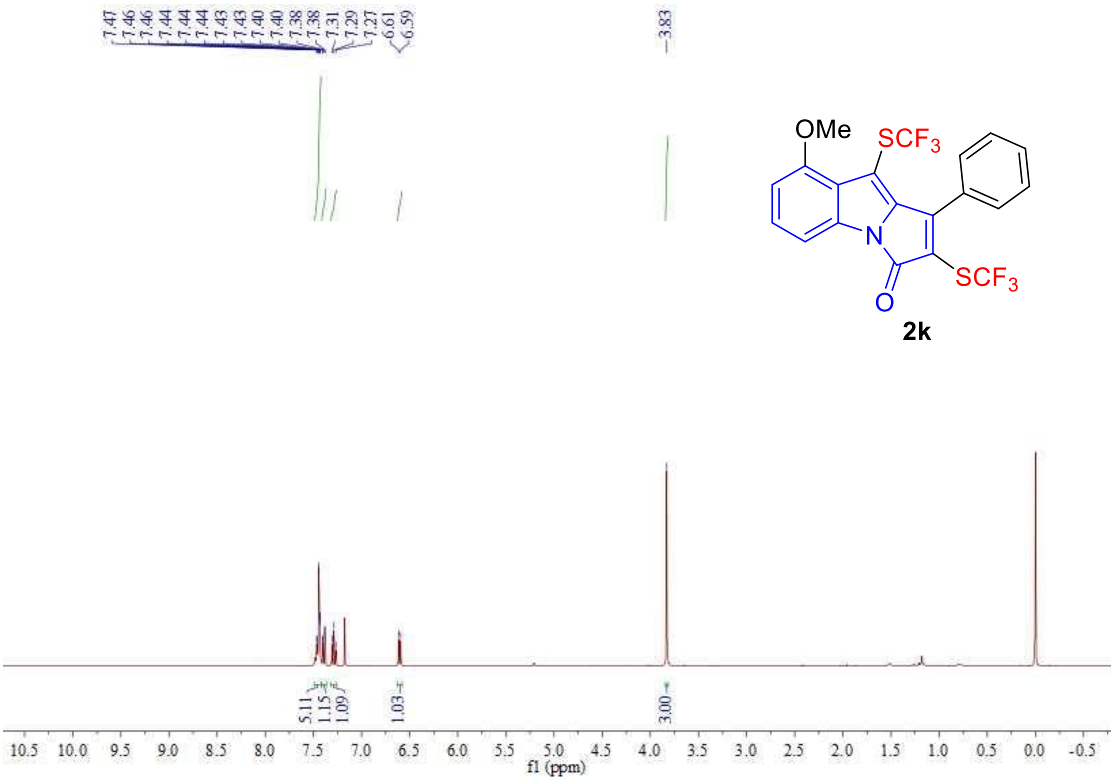

^{19}**F NMR** spectrum of **2k** in $CDCl_3$

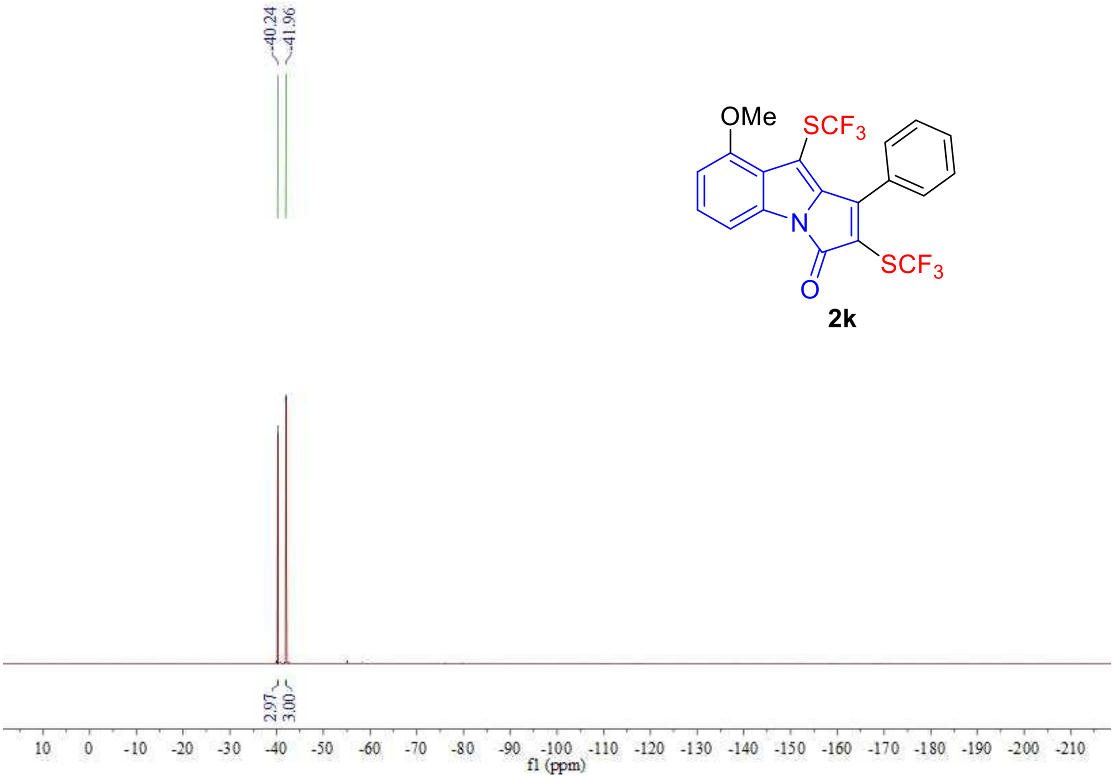

^{13}**C NMR** spectrum of **2k** in CDCl3

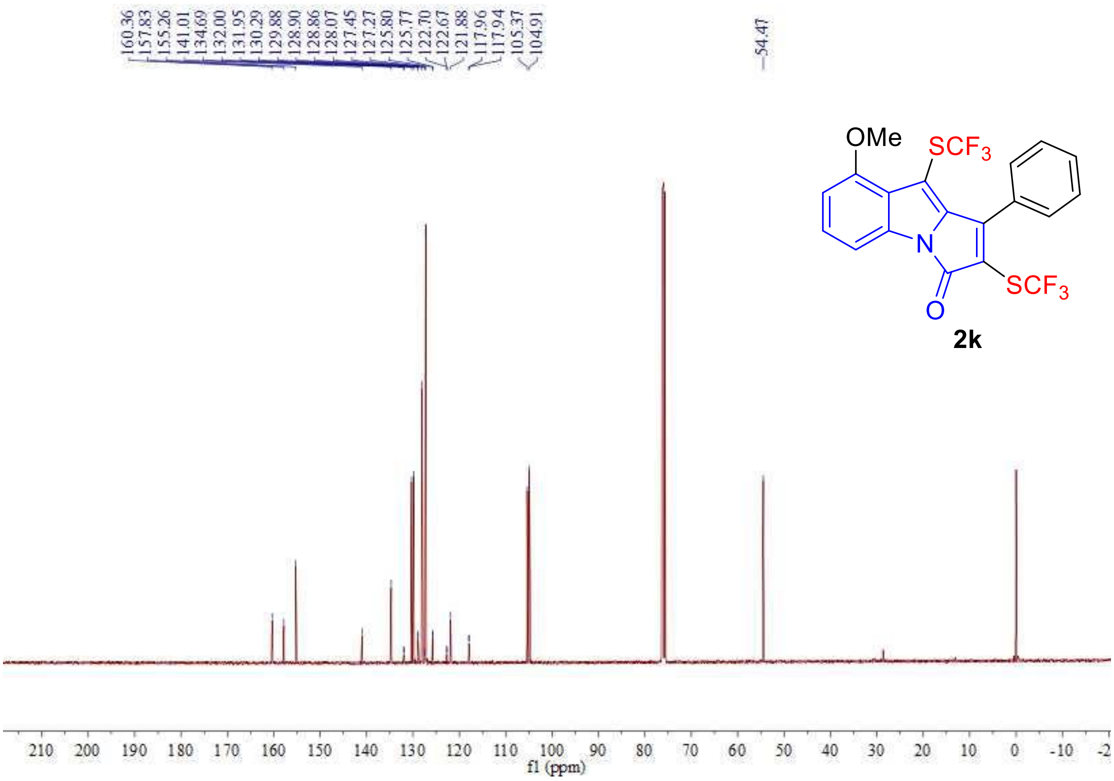

^{1}**H NMR** spectrum of **2l** in CDCl3

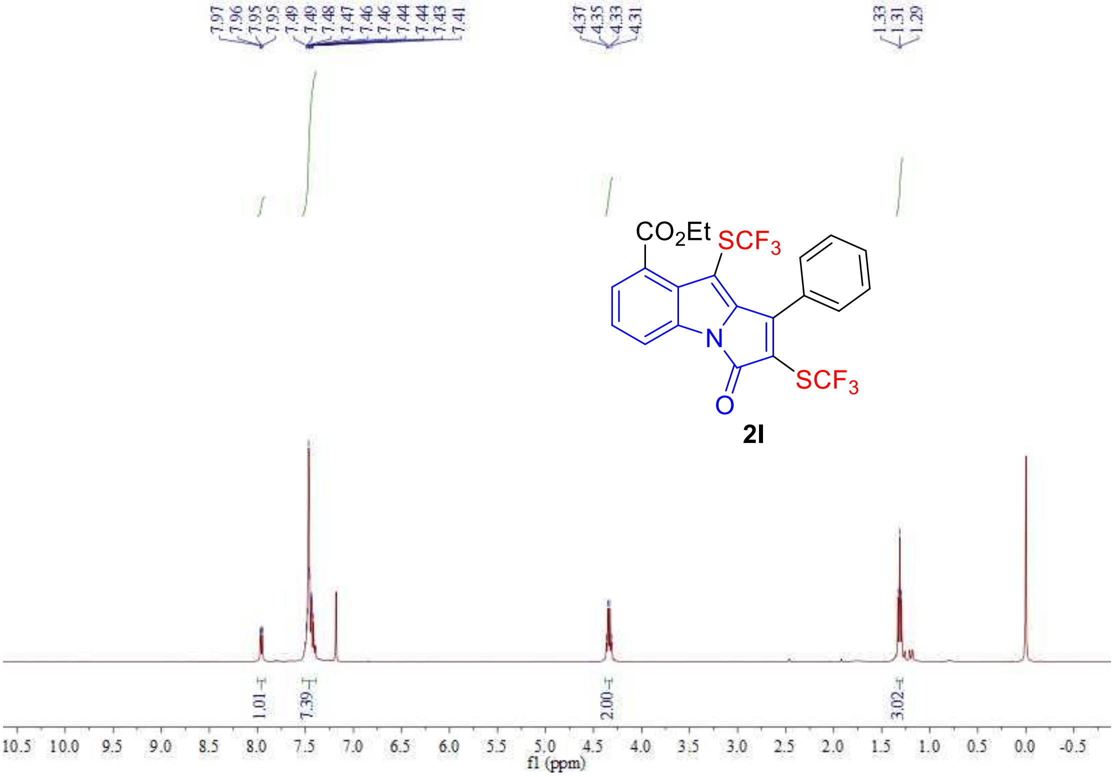

^{19}**F NMR** spectrum of **2l** in CDCl3

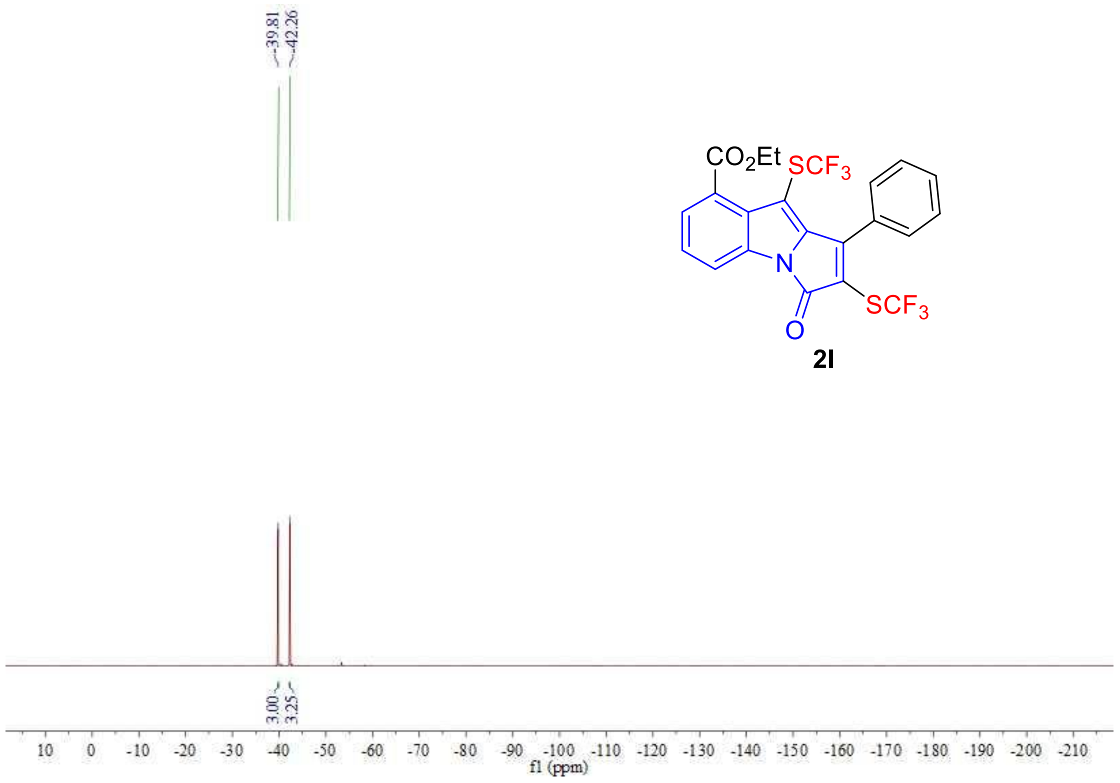

^{13}**C NMR** spectrum of **2l** in CDCl3

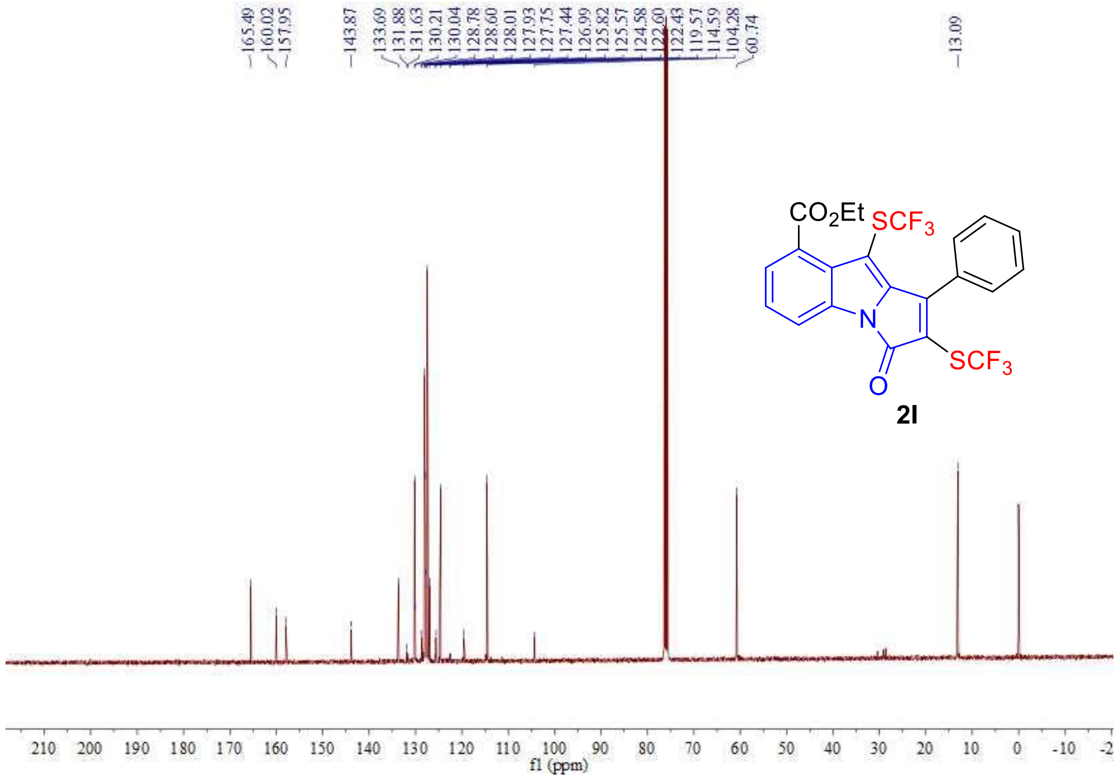

^{1}H NMR spectrum of **2m** in CDCl3

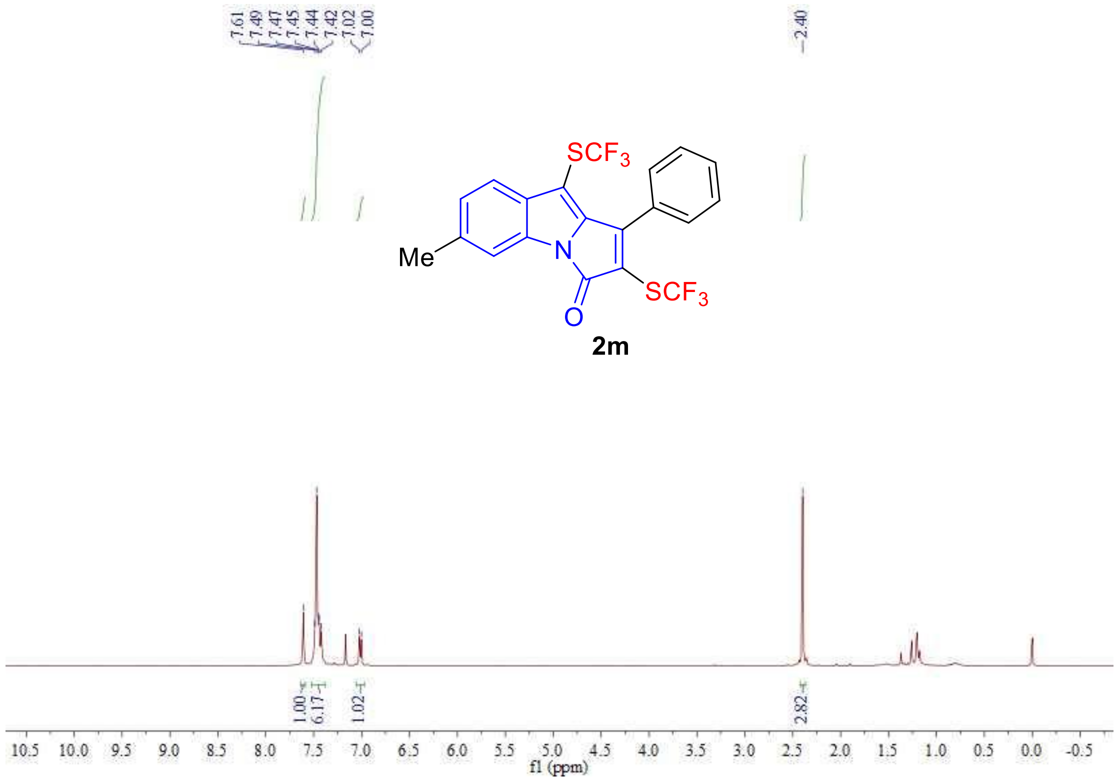

^{19}F NMR spectrum of **2m** in CDCl3

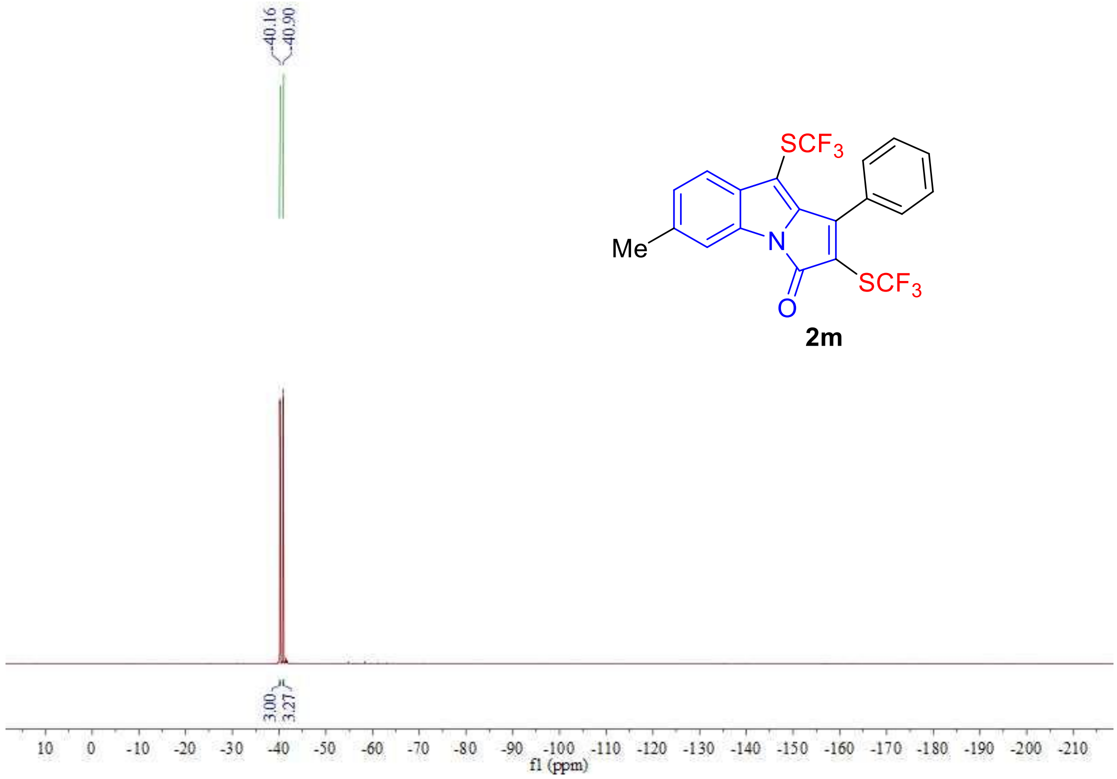

^{13}**C NMR** spectrum of **2m** in $CDCl_3$

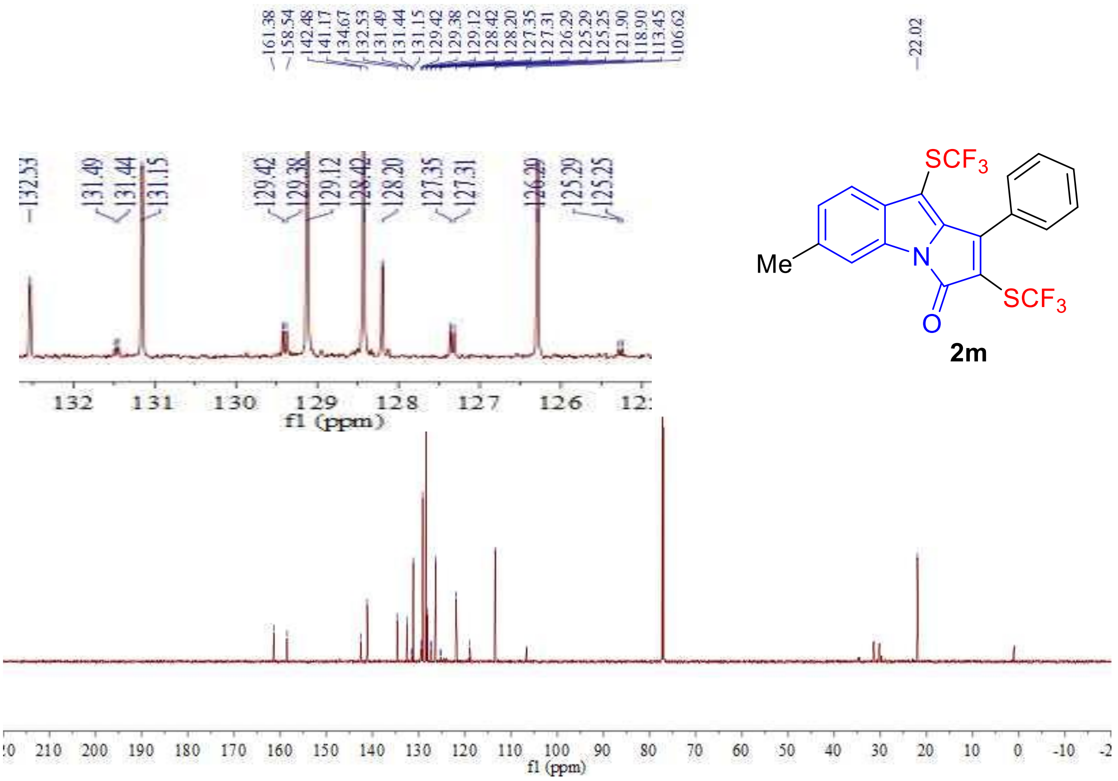

^{1}**H NMR** spectrum of **2n** in $CDCl_3$

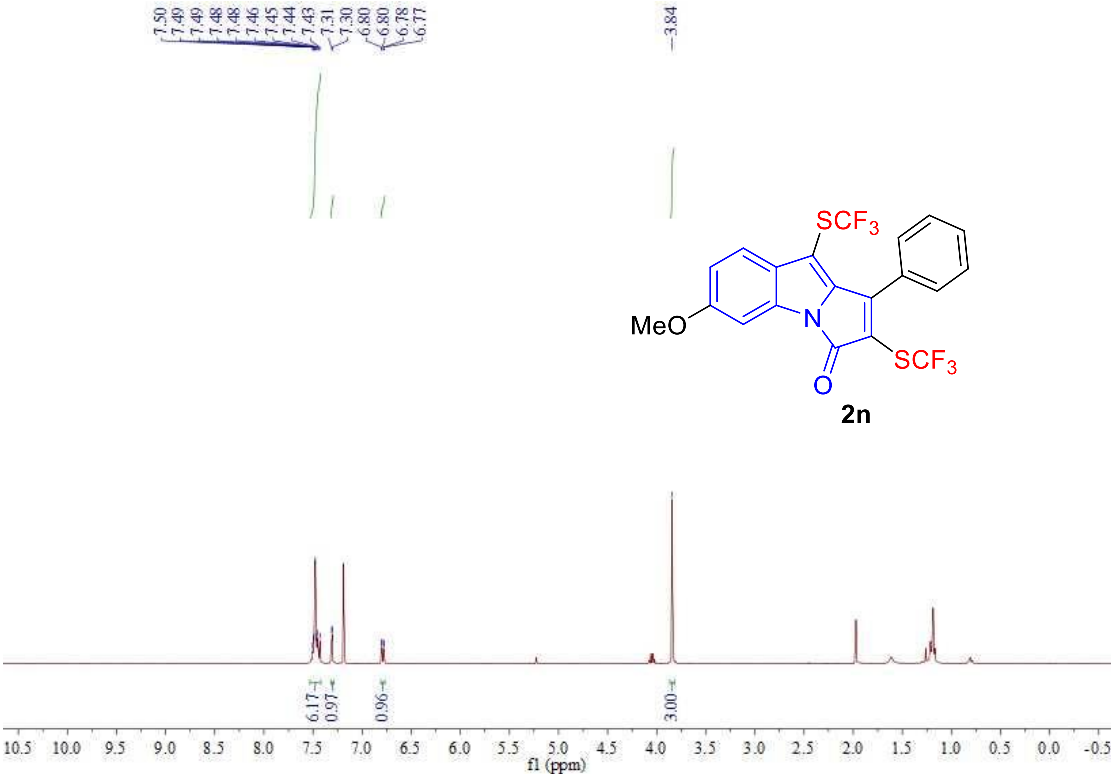

^{19}F NMR spectrum of **2n** in CDCl3

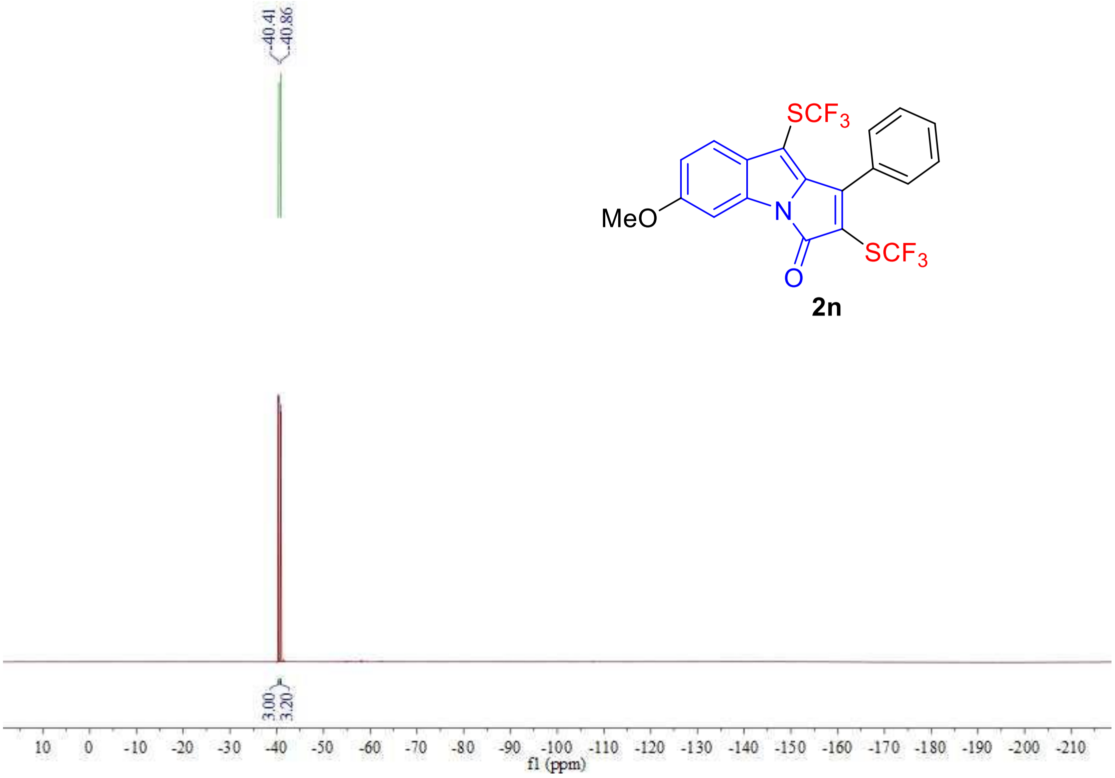

^{13}C NMR spectrum of **2n** in CDCl3

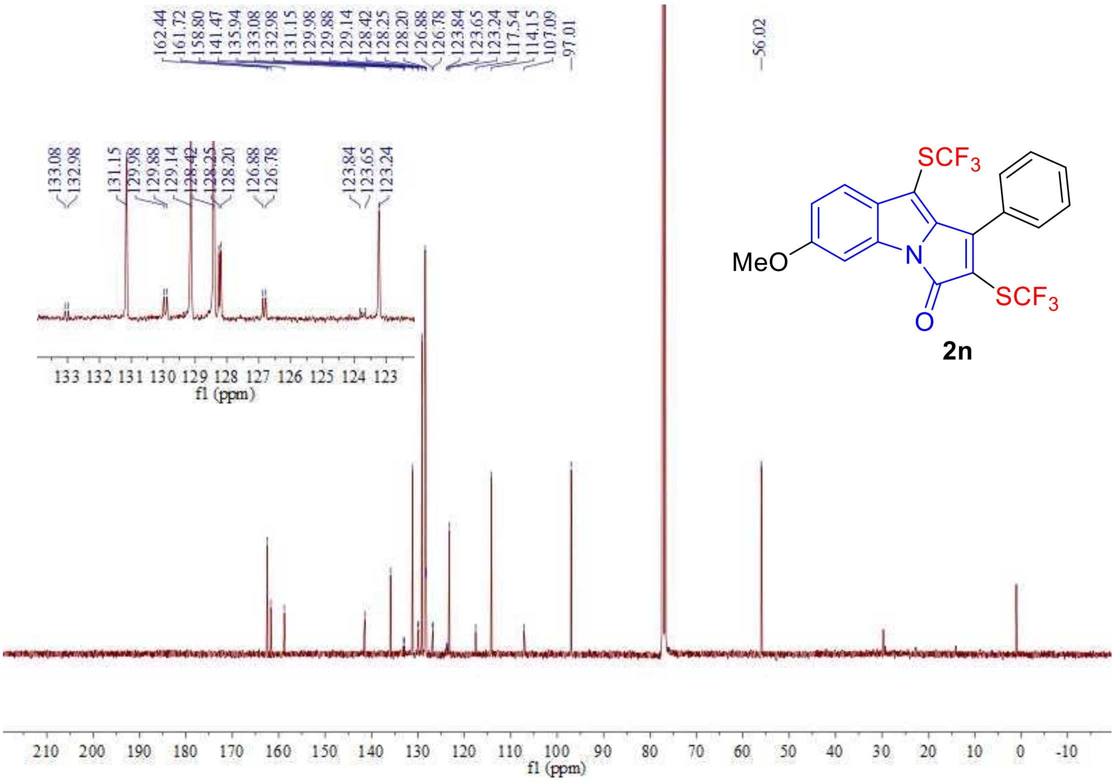

^{1}**H NMR** spectrum of **2o** in $CDCl_3$

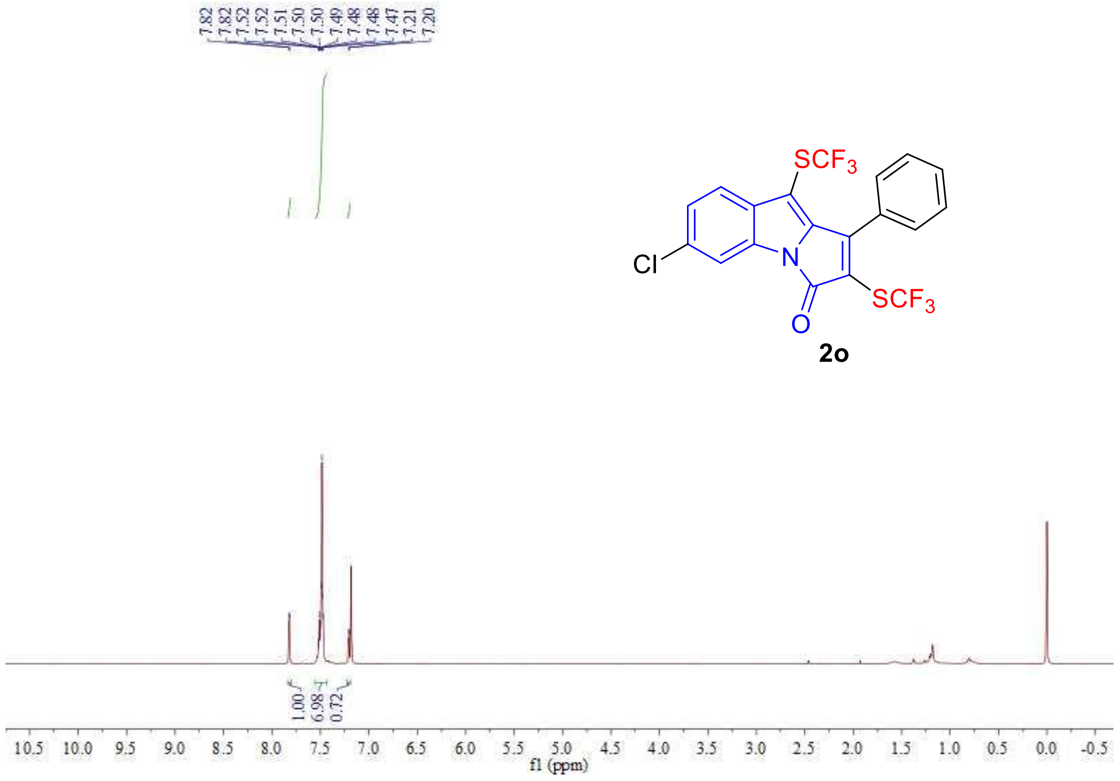

^{19}**F NMR** spectrum of **2o** in $CDCl_3$

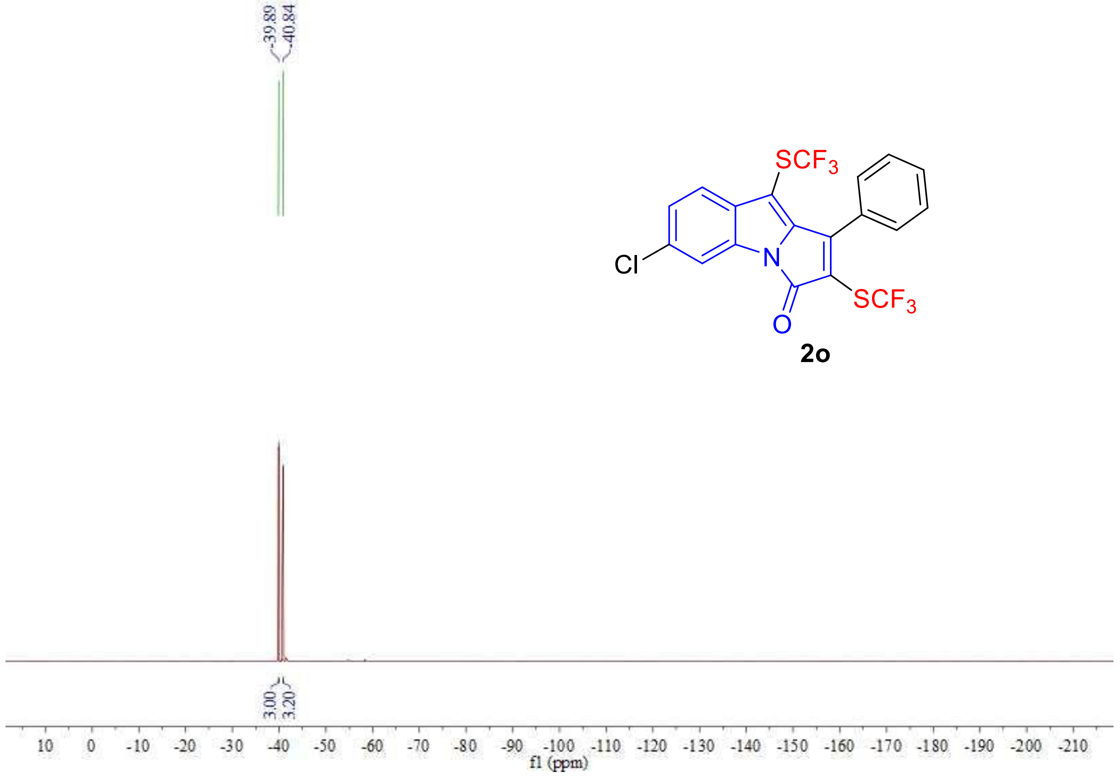

^{13}C NMR spectrum of **2o** in CDCl3

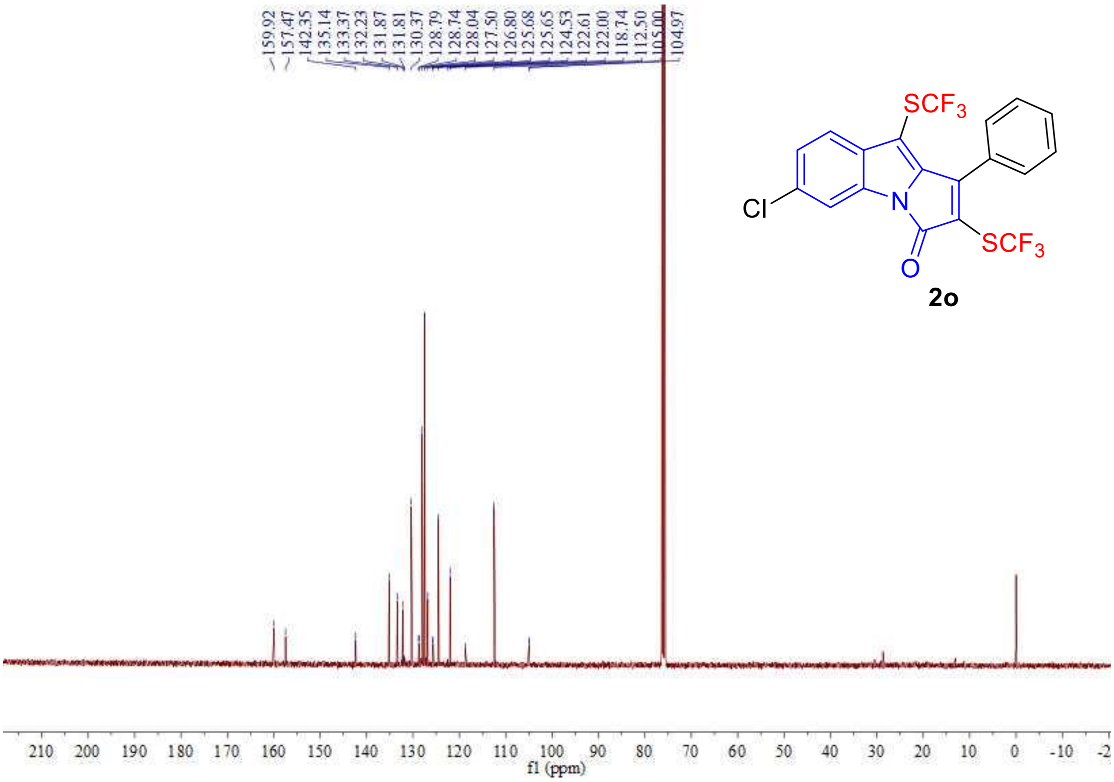

^{1}H NMR spectrum of **2p** in CDCl3

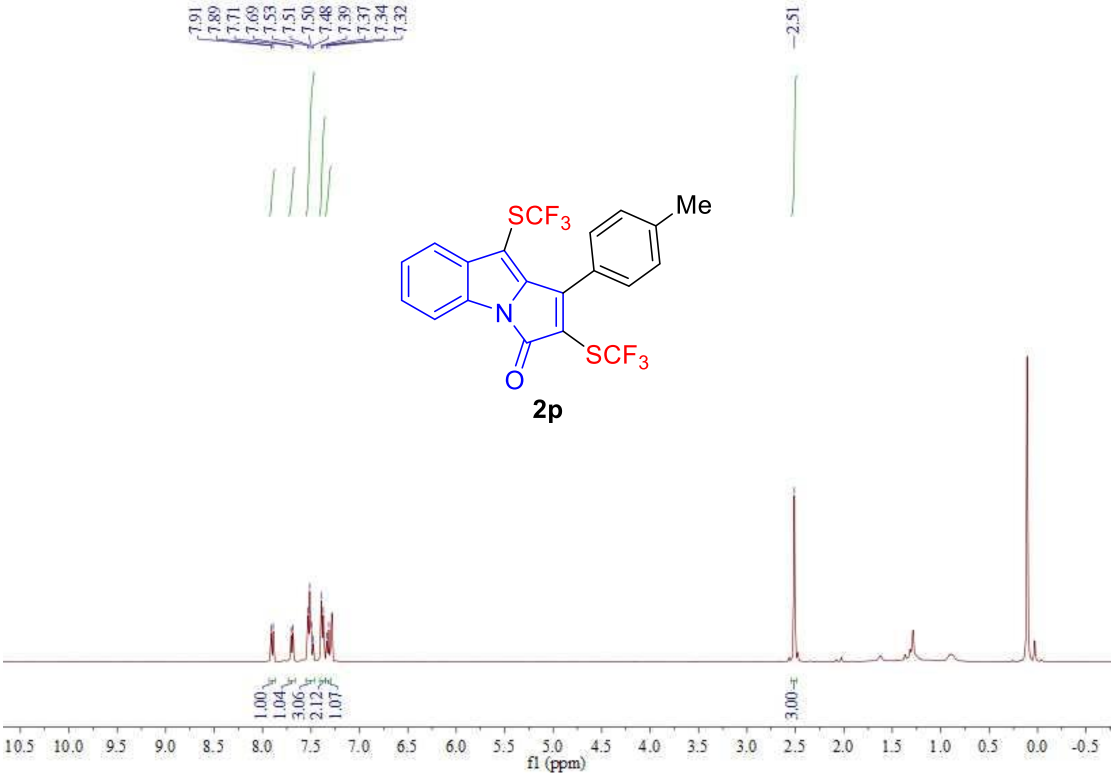

^{19}**F NMR** spectrum of **2p** in $CDCl_3$

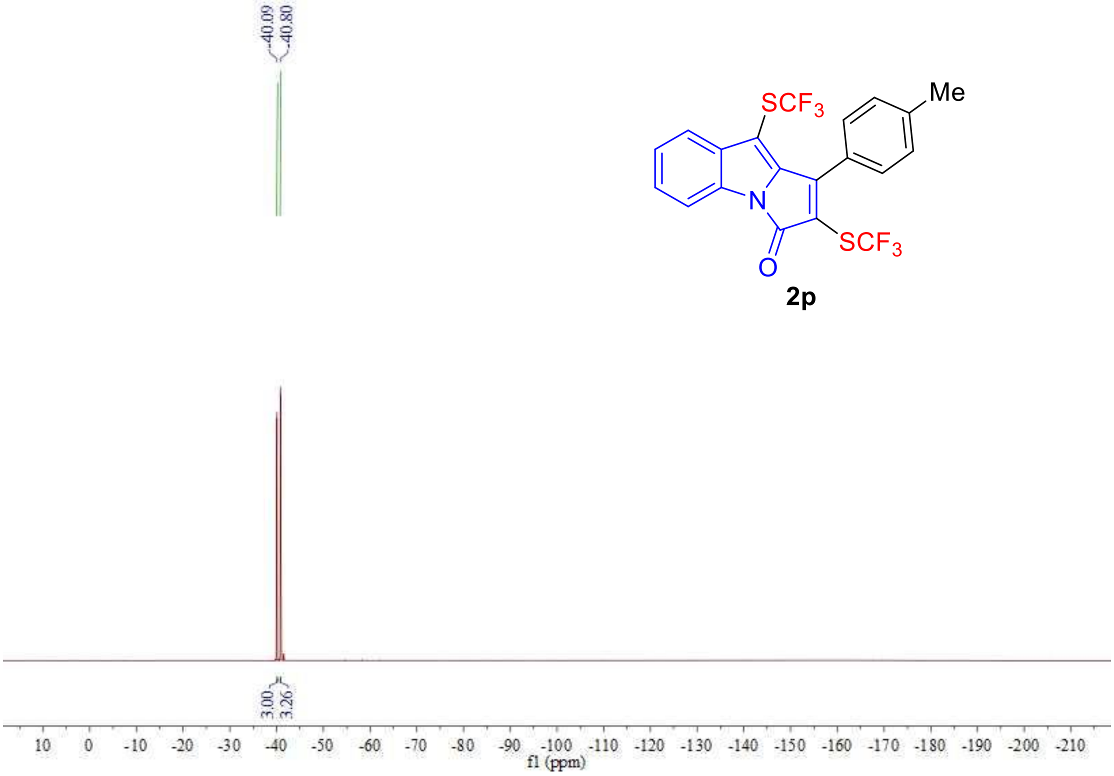

^{13}**C NMR** spectrum of **2p** in $CDCl_3$

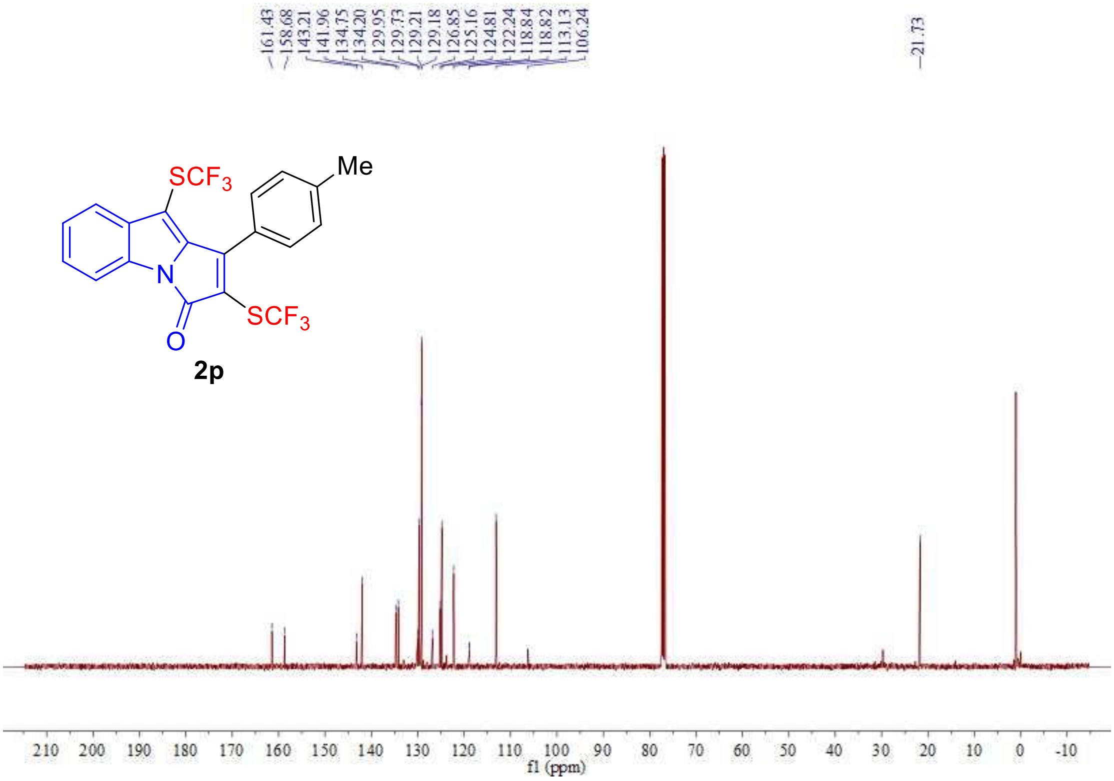

^{1}H NMR spectrum of **4a** in $CDCl_3$

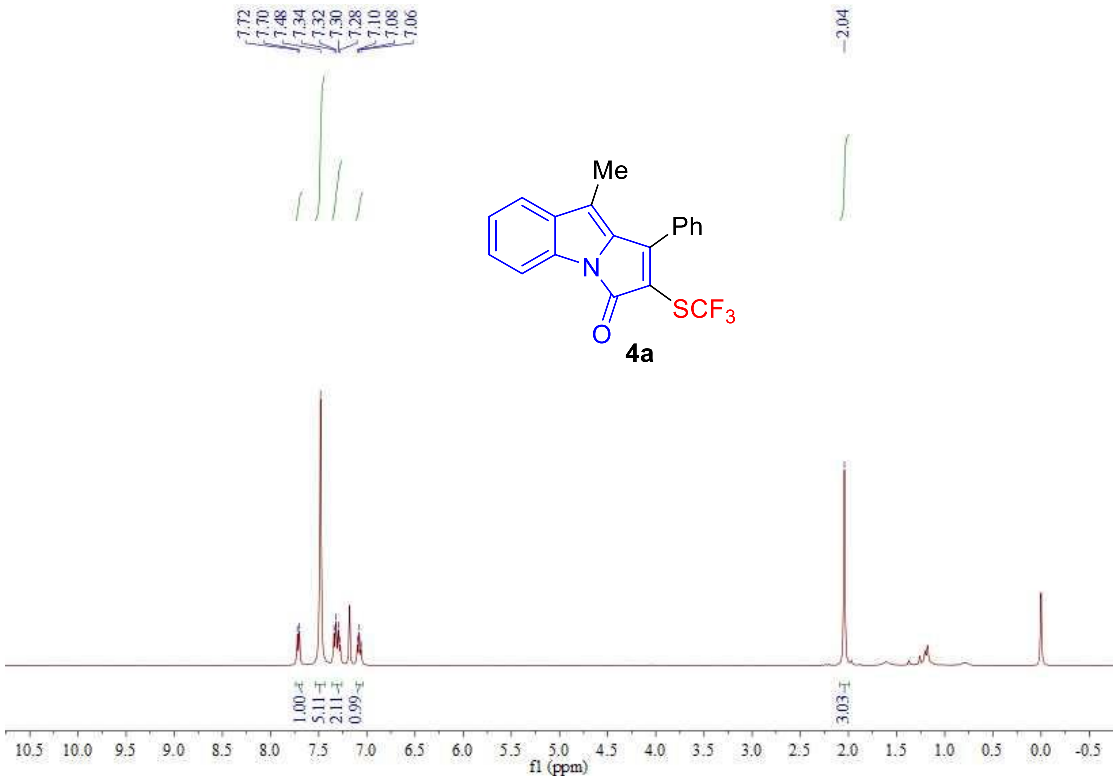

^{19}F NMR spectrum of **4a** in $CDCl_3$

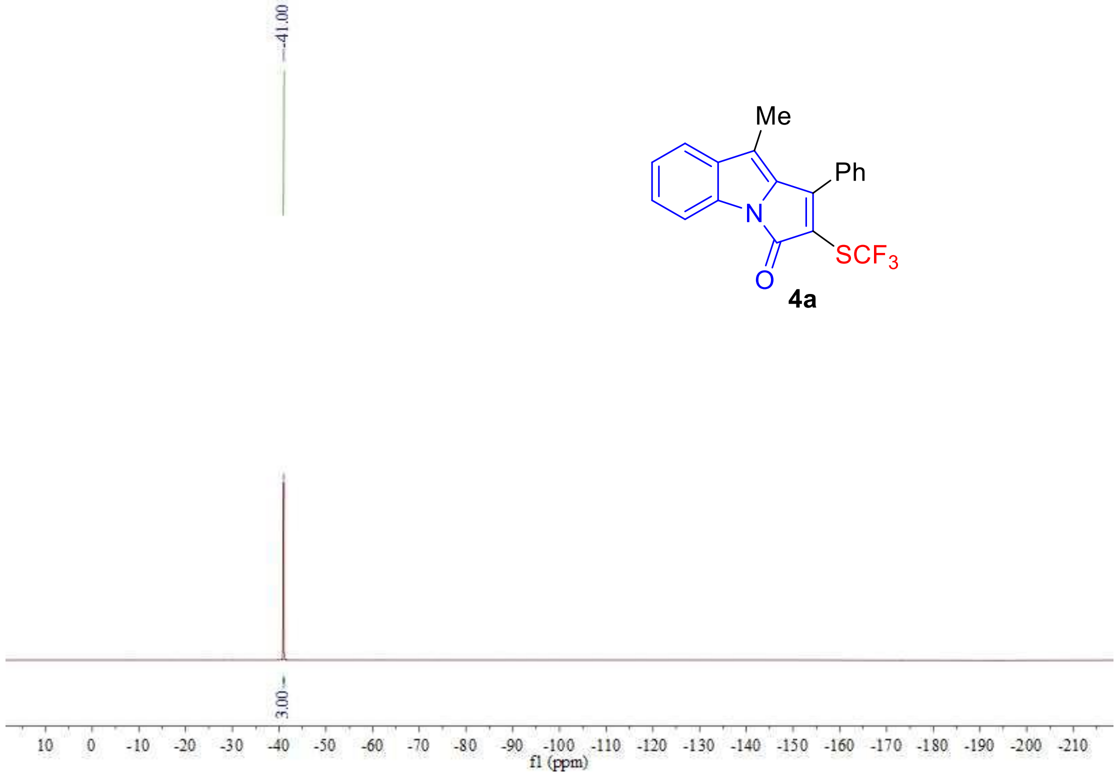

^{13}C NMR spectrum of **4a** in CDCl3

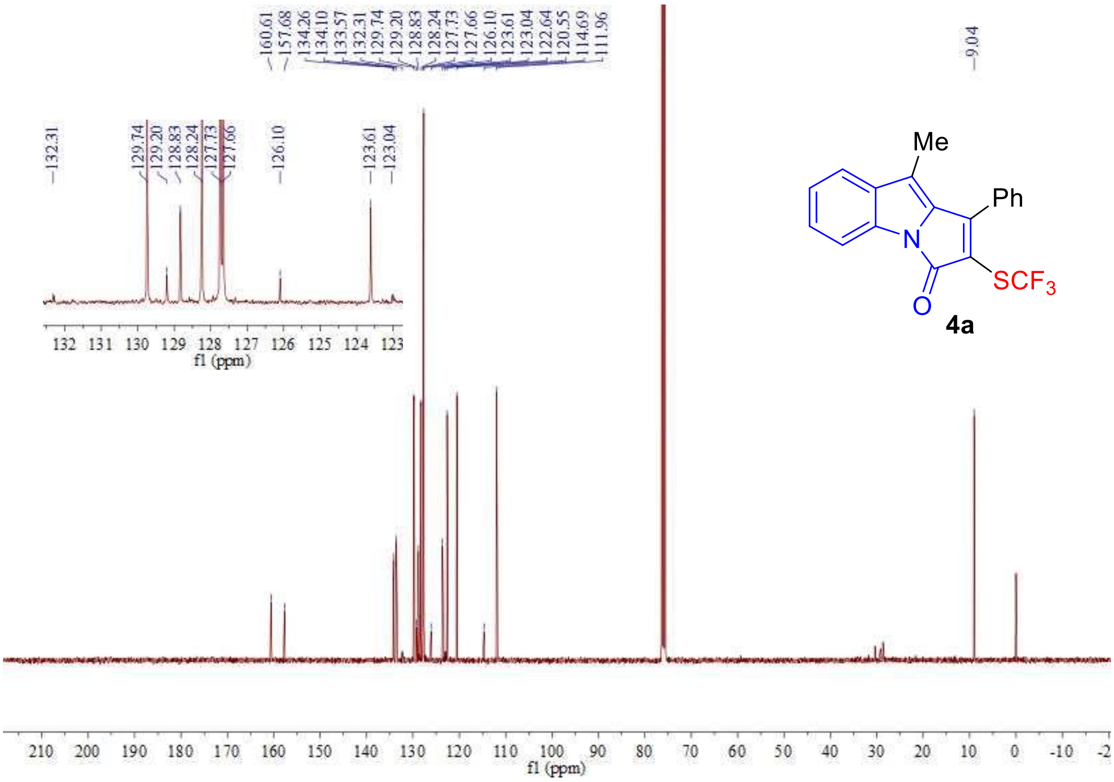

^{1}H NMR spectrum of **4b** in CDCl3

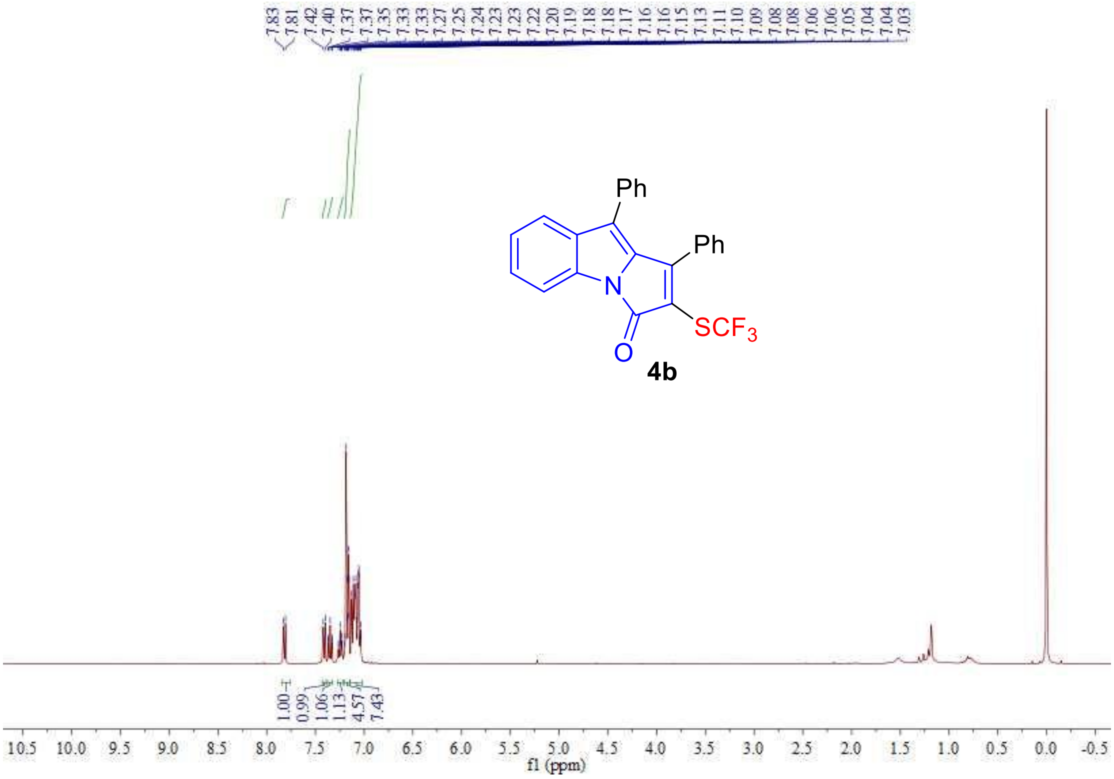

^{19}F **NMR** spectrum of **4b** in $CDCl_3$

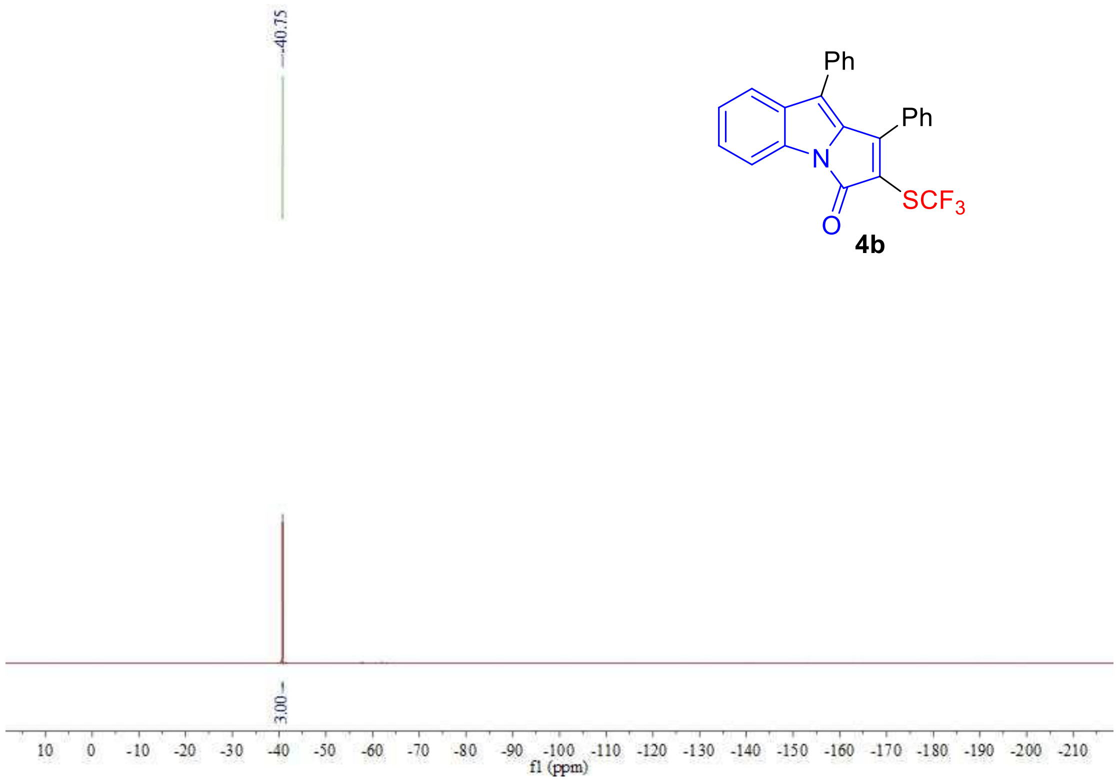

^{13}C **NMR** spectrum of **4b** in $CDCl_3$

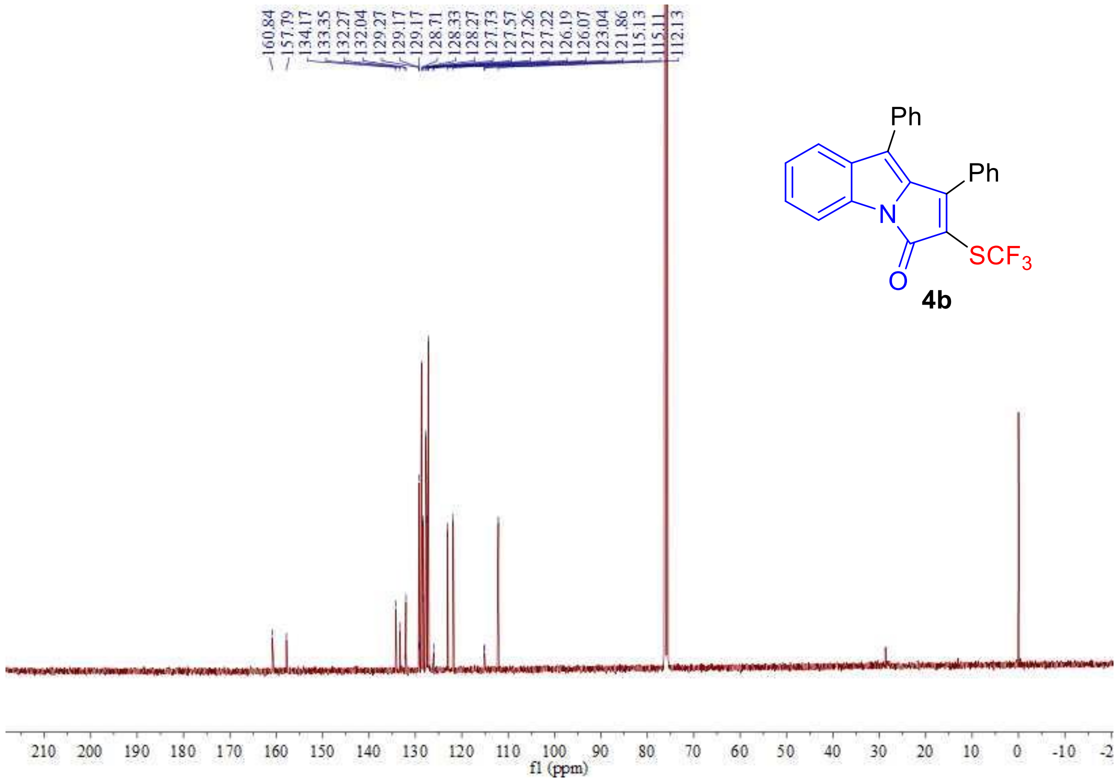

^{1}H NMR spectrum of **4c** in CDCl3

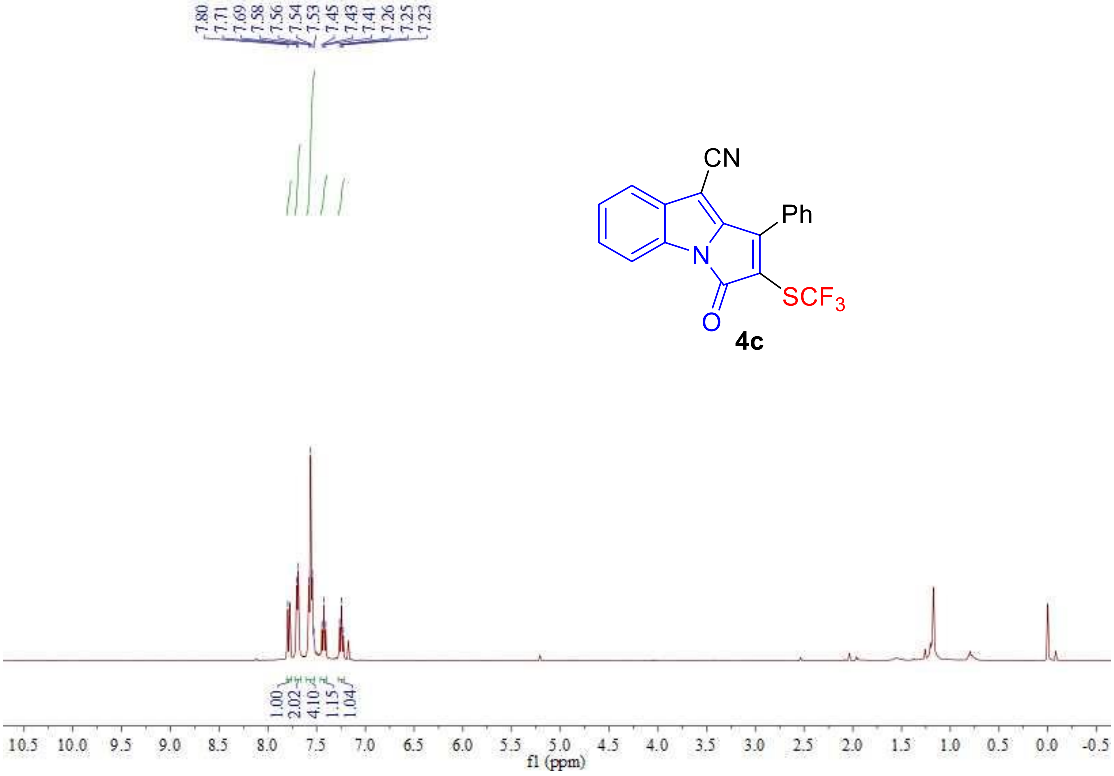

^{19}F NMR spectrum of **4c** in CDCl3

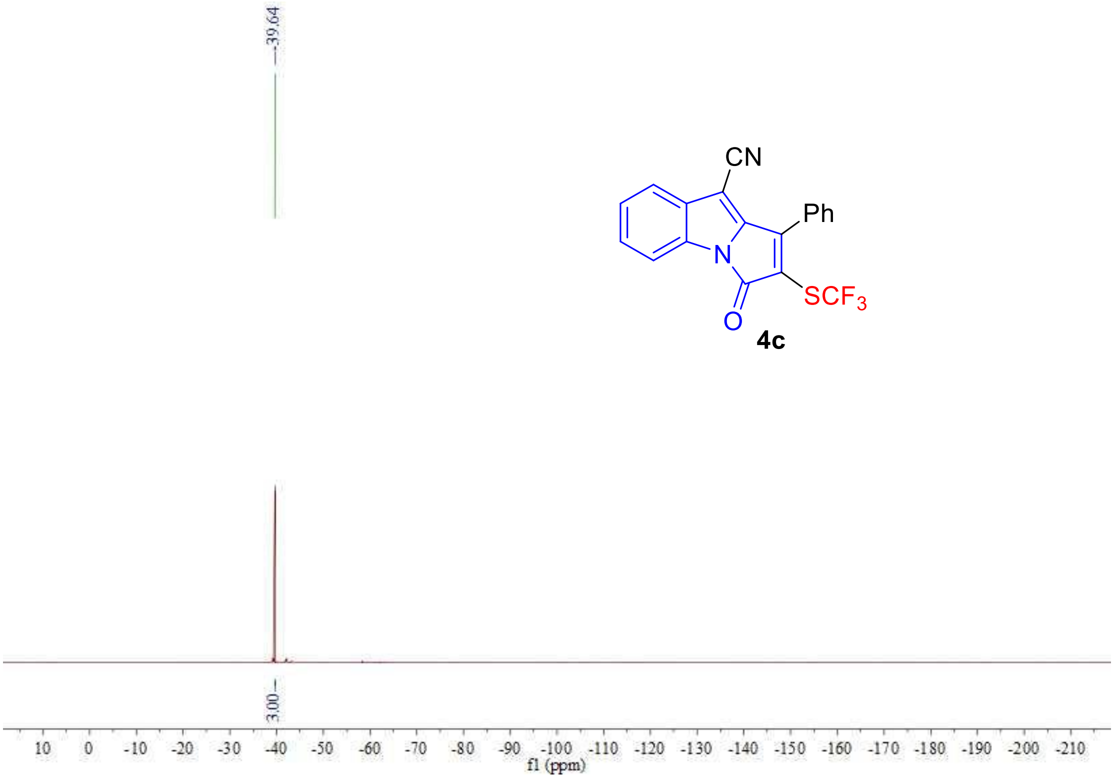

^{13}C NMR spectrum of **4c** in CDCl3

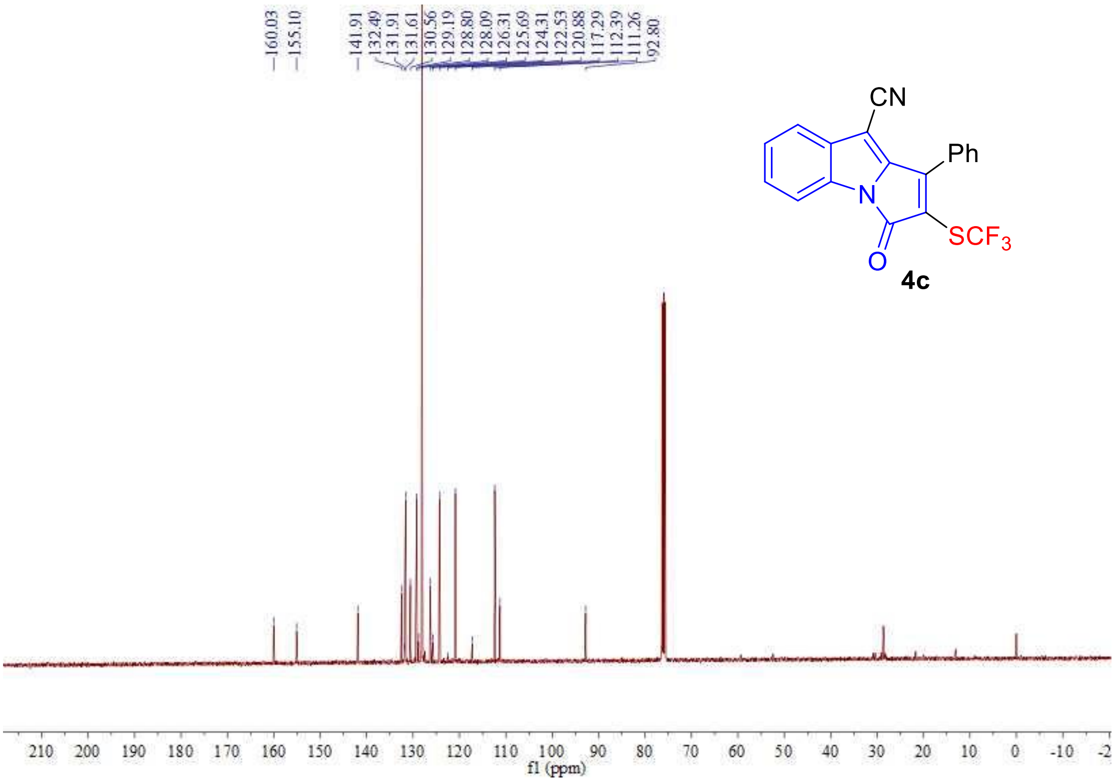

^{1}H NMR spectrum of **4d** in CDCl3

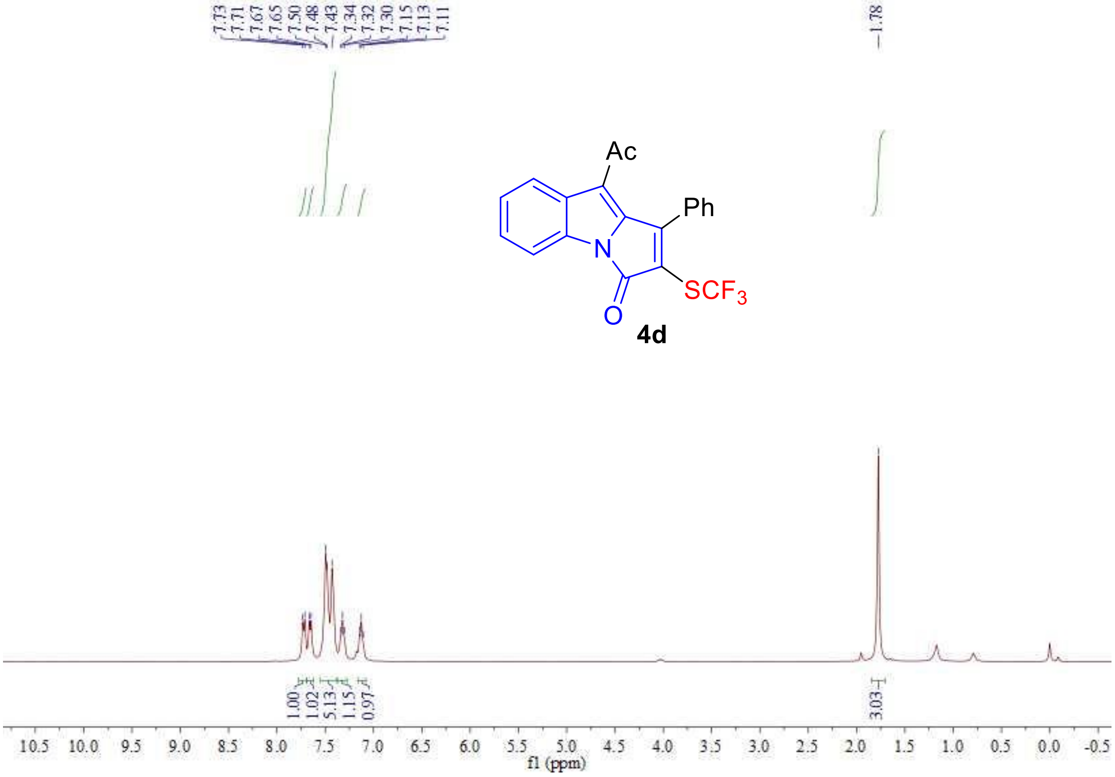

^{19}**F NMR** spectrum of **4d** in $CDCl_3$

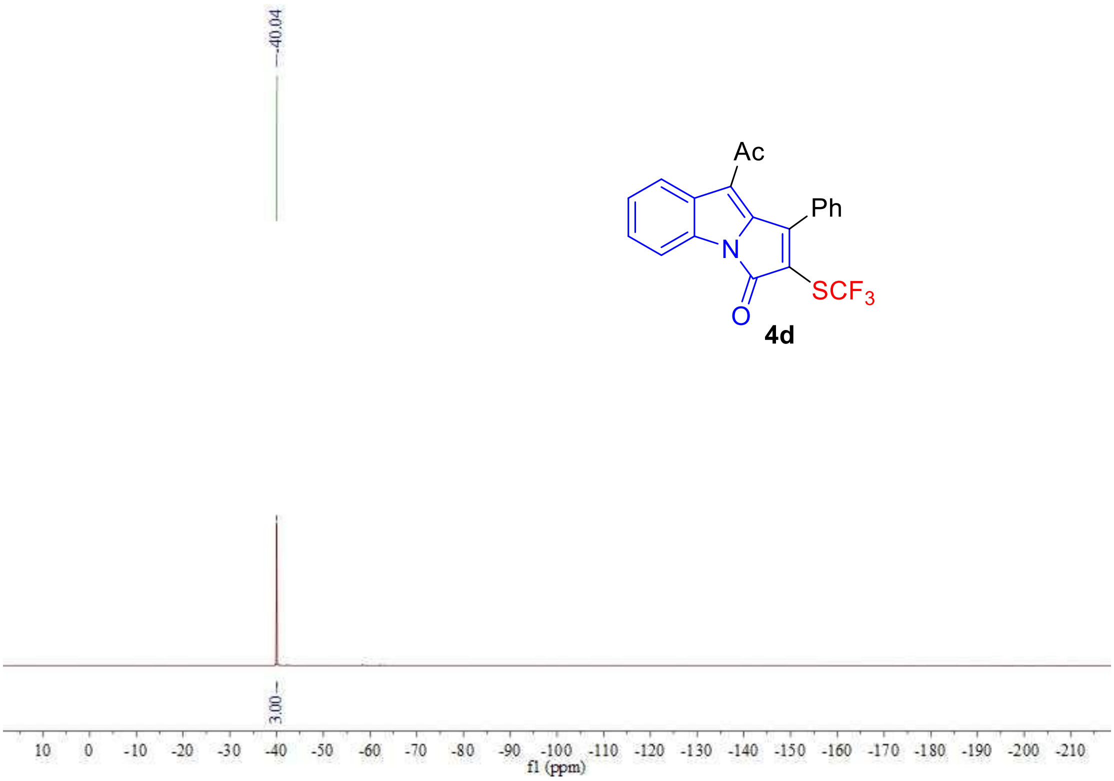

^{13}**C NMR** spectrum of **4d** in $CDCl_3$

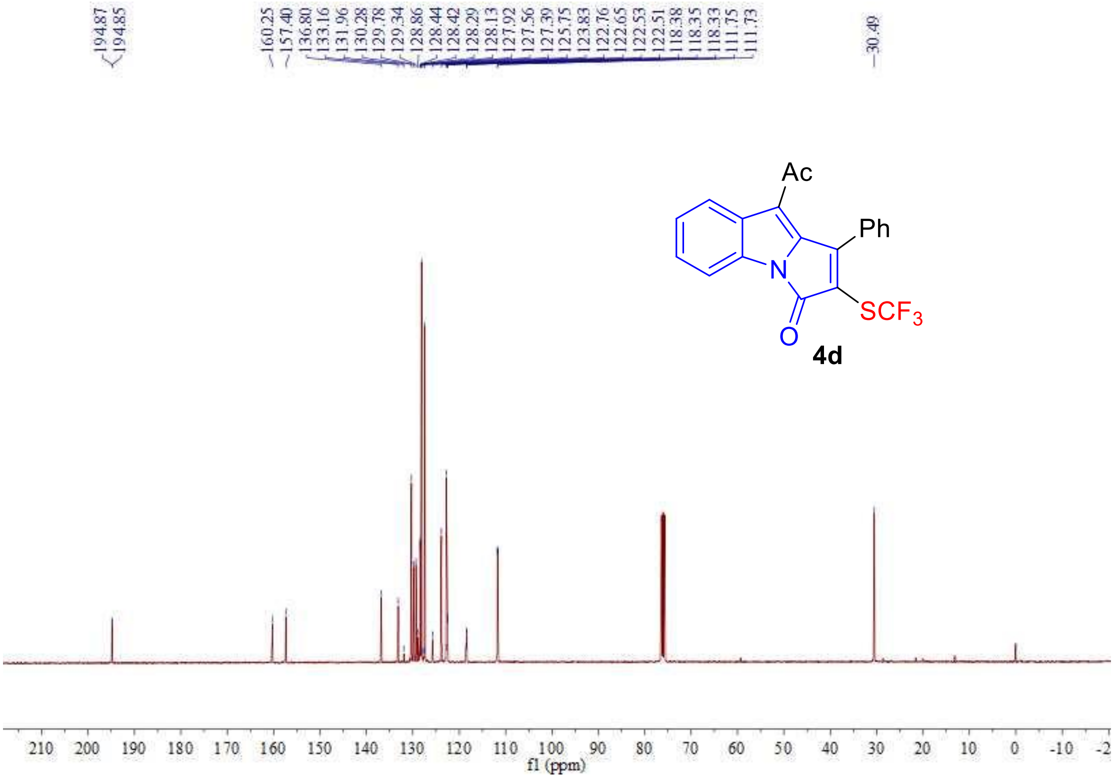

Supplement: File 1 — Experimental procedures, spectroscopic and X-ray data (CCDC 1968129 for compound 2a) and copies of NMR spectra. [file Beilstein_J_Org_Chem-16-657-s001.pdf]
